# ASCT2 Regulates Fatty Acid Metabolism to Trigger Glutamine Addiction in Basal-like Breast Cancer

**DOI:** 10.3390/cancers16173028

**Published:** 2024-08-30

**Authors:** Jia Wang, Qian Zhang, Huaizi Fu, Yi Han, Xue Li, Qianlin Zou, Shengtao Yuan, Li Sun

**Affiliations:** New Drug Screening and Pharmacodynamics Evaluation Center, China Pharmaceutical University, Nanjing 210009, China; 3123074292@stu.cpu.edu.cn (J.W.); 3219071101@stu.cpu.edu.cn (Q.Z.); fuhuaizi@163.com (H.F.); 3321071765@stu.cpu.edu.cn (Y.H.); 3221071632@stu.cpu.edu.cn (X.L.); 3222071660@stu.cpu.edu.cn (Q.Z.)

**Keywords:** glutamine, ASCT2, lipid metabolism, basal-like breast cancer

## Abstract

**Simple Summary:**

In recent years, cancer has gradually become a terrible killer of human health. Due to various factors such as tumor heterogeneity and the complexity of the microenvironment, completely conquering cancer has always been a major problem in the scientific research community. Therefore, more detailed research and individualized treatment are essential. Everyone knows that tumor cells in the process of rapid proliferation need replenishing nutrients to sustain their energy supply. Intervention of tumor cell metabolism is gradually becoming a way to treat cancer. As an important transporter of glutamine, ASCT2 can uptake glutamine from the tumor microenvironment to support the growth and metabolism of cancer cells. Deficiency of ASCT2 can significantly inhibit tumor progression. However, not all patients benefit from this treatment. Our study aims to explore the metabolic heterogeneity of breast cancer and its related mechanisms, as well as to find metabolic-sensitive populations for further precision treatment.

**Abstract:**

As a crucial amino acid, glutamine can provide the nitrogen and carbon sources needed to support cancer cell proliferation, invasion, and metastasis. Interestingly, different types of breast cancer have different dependences on glutamine. This research shows that basal-like breast cancer depends on glutamine, while the other types of breast cancer may be more dependent on glucose. Glutamine transporter ASCT2 is highly expressed in various cancers and significantly promotes the growth of breast cancer. However, the key regulatory mechanism of ASCT2 in promoting basal-like breast cancer progression remains unclear. Our research demonstrates the significant change in fatty acid levels caused by ASCT2, which may be a key factor in glutamine sensitivity. This phenomenon results from the mutual activation between ASCT2-mediated glutamine transport and lipid metabolism via the nuclear receptor PPARα. ASCT2 cooperatively promoted PPARα expression, leading to the upregulation of lipid metabolism. Moreover, we also found that C118P could inhibit lipid metabolism by targeting ASCT2. More importantly, this research identifies a potential avenue of evidence for the prevention and early intervention of basal-like breast cancer by blocking the glutamine–lipid feedback loop.

## 1. Introduction

Amino acids are essential nutrients for viable cells, and tumor cells have a higher requirement than normal cells [1]. Glutamine, an abundant amino acid, provides the nitrogen and carbon sources needed to support a series of reactions such as cancer cell proliferation, invasion, and metastasis [2]. Glutamine can generate metabolic intermediates with the help of glutaminase (GLS) and glutamate dehydrogenase1(GLUD1) entry into the tricarboxylic acid (TCA) cycle [3]. The TCA cycle is an essential hub for energy generation and the interconversion of metabolites, which are constantly renewed in rapidly proliferating cancer cells. In addition, glutamine can provide the precursors of other amino acids, including alanine, aspartate, serine, valine, leucine, and tyrosine.

Cancer cells alter their dependencies on different metabolic pathways to increase energy demands to maintain tumorigenicity and survive in the constantly changing microenvironment [4]. Many studies have shown that heterogeneity is presented in glutamine metabolism in breast cancer, and different molecular subtypes have significant discrepancies in glutamine dependence [5,6,7]. Breast cancer has five common molecular types: Luminal A, Luminal B, HER2^+^, normal-like, and basal-like [8]. It is well known that basal-like breast cancer (BLBC) is characterized by high heterogeneity, poor prognosis, and susceptibility to recurrence and metastasis and is mainly treated through chemotherapy [9]. Moreover, it has some unique metabolic features, such as increased aerobic glycolysis, increased fatty acid synthesis, and fatty acid oxidation, and exhibits a glutamine dependence [10]. There are also reports that glutamine addiction increases TCA cycle fluxes and replenishes TCA cycle intermediates to promote glucose oxidation in triple-negative breast cancer. Accordingly, it is urgent to explore the metabolic characteristics of breast cancer and find effective metabolic targets for treatment.

Alanine/serine/cysteine Transporter 2(ASCT2) can transport threonine, alanine, serine, asparagine, cysteine, glutamine, etc. It is the main extracellular-to-intracellular transporter of glutamine and can provide an important substrate for rapidly growing tumor cells [11]. Therefore, it has been found that ASCT2 is highly expressed in various cancers to maintain high nutrient requirements for cancer cells [12,13,14,15] (e.g., melanoma, prostate cancer, gastric cancer, non-small-cell lung cancer, etc.). Deletion of the ASCT2 inhibits the growth of breast cells, which may make it a potential drug target for tumor treatment [16]. As a consequence, the development of ASCT2 inhibitors has broad prospects, such as V9302 [17], GPNA [18], ASCT2 monoclonal antibody [19,20], etc. Currently, the discovery of selective and potent inhibitors of ASCT2 and glutamine uptake is evolving slowly. Furthermore, the compensatory mechanisms of ASCT2 inhibition remain to be clarified in the future [21]. Therefore, the application of these inhibitors is limited and not therapeutic for all tumor types, which makes finding specific cancer groups an urgent scientific problem.

Lipid metabolism is an important cellular process that converts nutrients into metabolic intermediates for membrane biosynthesis, energy storage, and the production of signaling molecule uptake [22]. When the intracellular lipid content is excessive, they will be converted into glyceryl triesters and cholesteryl esters, forming lipid droplets to store energy in response to the dynamic cellular energy metabolism demand [23]. Additionally, fatty acids can also be converted to acylcarnitine by carnitine palmitoyltransferase 1 (CPT1) and then shuttled into mitochondria for β-oxidation and energy generation [24]. The fatty acid oxidation (FAO) reaction is an indispensable source of ATP, NADH, and NADPH which provides a survival advantage for the growth of breast tumor cells [25]. The cells generally acquire lipids through two mechanisms, de novo synthesis and uptake [26,27]. In terms of fatty acid synthesis, glucose is the main raw material for de novo fatty acid synthesis in oxygenated normal cancer cells. Glucose is converted to pyruvate by glycolysis, which enters the mitochondria to form citrate and is finally released into the cytoplasm as a precursor for fatty acid and cholesterol synthesis [28,29,30,31]. While under conditions of hypoxia, glutamine-derived α-ketoglutarate is converted to citrate, favoring de novo synthesis of lipids [32,33,34]. It was found that a small-molecule inhibitor of ASCT2 can inhibit the fatty acid metabolism pathway in prostate cancer [35]. This finding is of great interest given the prominent link between fatty acid synthesis and glutamine.

At present, the key target genes and signaling pathways downstream of ASCT2 promoting BLBC progress remain unclear. A better understanding of the molecular basis of the link between fatty acid metabolism and ASCT2 is of great clinical significance. Our research provides new insights into ASCT2 as a regulator of lipid metabolism, which has important implications for BLBC therapeutic targeting.

## 2. Materials and Methods

### 2.1. Antibodies

Anti-CPT1B(A6796) and anti-PPARα(A18252) were purchased from ABclonal Technology (Wuhan, China). Anti-ASCT2(ab187692) was purchased from Abcam (Cambridge, UK). Anti-β-actin was purchased from Cell Signaling Technology (CST, Danvers, MA, USA).

### 2.2. Inhibitors

GW6471 (HY-15372) was purchased from MedChem Express (Monmouth Junction, NJ, USA). C118P (C18H17N2Na2O7P, 450.29, powder injection) was provided by Nanjing Shenghe Pharmaceutical Co., Ltd. (Nanjing, China) TAX (national character standard H10980069) was purchased from Beijing Union Pharmaceutical Factory (Beijing, China).

### 2.3. Cell Culture

HCC1806, MDAMB-436, KPL4, HCC1937, BT549, MDA-MB-453, T47D, and BT474 cells were maintained in Roswell Park Memorial Institute 1640 medium (RPMI 1640; GIBCO, Carlsbad, CA, USA) supplemented with 10% Fetal Bovine Serum (FBS). MDA-MB-231, MDA-MB-468, MCF-7, and SKBR3 were maintained in Dulbecco’s modified Eagle’s medium (DMEM; GIBCO, Carlsbad, CA, USA) supplemented with 10% FBS. The passage number of the cell lines was P3–P15. All cells were incubated at 37 °C under 5% CO_2_ in a humidified atmosphere. All cell lines were obtained from the cell bank of the Committee on Type Culture Collection of the Chinese Academy of Sciences (Shanghai, China) ATCC.

### 2.4. Plasmids

SLC1A5 were subcloned into PCDNA3.1-tagged expression vectors. Transfection was performed with Lipofectamine 3000 (Invitrogen, Carlsbad, CA, USA) for 48 h.

### 2.5. Small Interfering RNA and Short Hairpin RNA

siSLC1A5 and non-silencing control siRNA were purchased from GenePharma. The targeted sequences were as follows:

siSLC1A5#1 5′-GCCUUGGCAAGUACAUUCUTT-3′;

siSLC1A5#2 5′-GUCGACCAUAUCUCCUUGATT-3′;

Negative control 5′-UUCUUCGAACGUGUCACGUTT-3′.

Transfection was performed with Lipofectamine 3000 (Invitrogen, Carlsbad, CA, USA) for 8 h.

### 2.6. LDH Measurement

Cells were seeded at a density of 3000 cells/well in a 96-well plate. After 24 h, cells were incubated with 200 μL glutamine-free DMEM/1640 medium with 10% dialyzed FBS (Medium:Serum = 9:1). After 96 h of treatment, the assay was performed with the LDH detection kit (C0018S, Beyotime, Shanghai, China) according to the manufacturer’s instructions.

### 2.7. ATP Measurement

Cells were seeded at a density of 3000 cells/well in a 96-well plate. After 24 h, cells were incubated with 200 μL glutamine-free DMEM/1640 medium (meilunbio, Dalian, China) and supplemented with 10% dialyzed FBS. After 96 h of treatment, the assay was performed with the CellTiter-Glo Luminescent Cell Viability Kit (DD1101-01, Vazyme, Nanjing, China) according to the manufacturer’s instructions.

### 2.8. Lipid Droplet Staining

Cells were treated with siRNA or a drug and induced with 100 µM oleate-coupled BSA for 18 h. Cells were rinsed twice with PBS and postfixed with 4% paraformaldehyde for 20 min at room temperature. Cells were rinsed three times with PBS before and after staining with a freshly prepared Oil red O staining kit (Beyotime, China) for 30 min. The cells were infiltrated with 60% isopropanol for 10 s and washed 3 times with 1× PBS. Hematoxylin staining lasted 1 min, and 1× PBS washing was performed twice. All images from Oil red O staining were obtained by an inverted microscope (Olympus, Hachioji-shi, Japan) and were analyzed using Image J (https://ij.imjoy.io). Lipid droplets were quantified as follows: Relative Area = Area of lipid droplet/number of cells.

### 2.9. Western Blot

Western blot analysis was performed with standard methods. Briefly, cells were lysed in radioimmunoprecipitation assay (RIPA) buffer containing protease inhibitors (Beyotime, China) and phosphatase inhibitors (Beyotime, China). Then, 20 μg proteins were separated by 8–12% SDS-PAGE, blotted onto PVDF membranes (Millipore, St. Louis, MI, USA), and blocked with 5% BSA (BSA dilution in 1× TBST). Membranes were incubated with specific primary antibodies (1:1000 dilution) overnight at 4 °C and then incubated for 1 h with HRP-coupled secondary mouse or rabbit antibodies (Abcam, Cambridge, UK), which were generated by Chemiluminescent HRP Substrate (Millipore, USA). The membrane was exposed in chemiluminescence imaging.

### 2.10. RNA Isolation and RT-qPCR

Total RNA was isolated using the TRIzol Reagent (Vazyme, Nanjing, China), and cDNA was generated from 1 μg total RNA per sample using HiScript qRT SuperMix (Vazyme, China) and miRNA 1st Strand cDNA Synthesis Kit (Vazyme, China), respectively, for mRNA and mature miRNA. RT-qPCR was performed by using the SYBR Green master mix (Vazyme, China) and commercially available primers.

### 2.11. Fatty Acid Oxidation (FAO) Assay

FAO measurement was performed following the manufacturer’s instructions using Fatty Acid Oxidation Assay Kits from Abcam (ab217602). Briefly, 3 × 10^4^ cells were seeded per well in 96-well plates and cultured overnight. Then, the cells were rinsed twice with 100 μL pre-warmed FA-Free medium, followed by adding 90 μL pre-warmed FA measurement medium. The wells without cells were used as signal controls. A total of 85 μL of FA-free measurement medium was added to the wells, and 5 μL of BSA control was included as the FA-free control. All wells except the blank control had 10 μL extracellular O_2_ consumption reagent added. The FAO activator FCCP (0.625 μM) and inhibitor Etomoxir (40 μM) were added as the positive and negative controls. Then, the wells were sealed with 100 μL pre-warmed mineral oil, and the FAO was measured using the condition of extracellular oxygen consumption. The results were normalized by the protein concentration with the cells in each sample under the BCA assay.

### 2.12. In Vivo Tumorigenesis Study

All work performed with animals was approved by the Institutional Animal Care Committee of China Pharmaceutical University. The study was performed using five- to six-week-old BALB/C nude mice (Nanjing University, Nanjing, China). A total of 4 × 10^6^ MDA-MB-231 cells were subcutaneously injected into the flanks of the mice. When the tumor volume reached about 100 mm^3^, the mice were randomly divided into groups of six individuals each, and 50 mg/kg and 100 mg/kg of C118P were injected 5 times a week. As a positive control, 10 mg/kg of TAX was injected 2 times a week. During this period, the tumor volume and the body weight of mice were recorded once every two days. After 21 days, the mice were sacrificed and the tumor tissues were photographed and extracted. Tumor volume (TV) was calculated as follows: TV = 1/2 × A × B^2^, where A and B represent the length and width of the tumor tissue, respectively (unit: mm). Subsequently, TV was plotted as a function of time.

## 3. Results

### 3.1. Glutamine Addiction in Basal-like Breast Cancer

**Figure 1:**
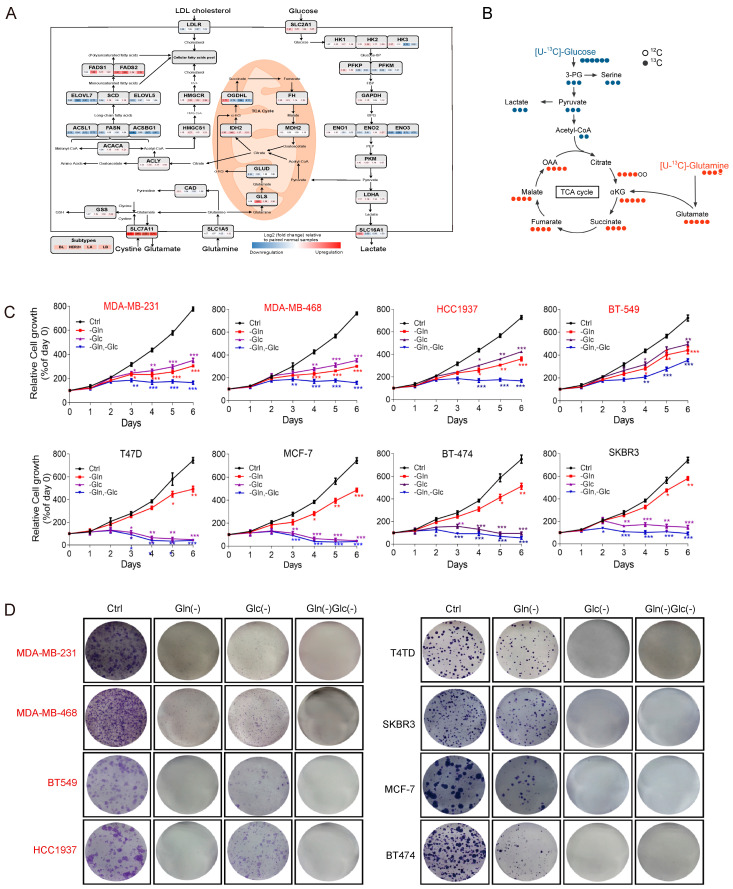
Glutamine addiction in basal-like breast cancer. (**A**) The key enzymes’ expressions of glutamine metabolism, glucose metabolism, TCA cycle, and fatty acid metabolism in clinical breast cancer issues were integrated from the TCGA database. (**B**) The tracer scheme of mass spectrometry analysis illustrates the flux of [^13^C_6_]-glucose (blue) and [^13^C_5_/^15^N_2_]-glutamine (red) for 24 h. (**C**) The effects of glutamine and glucose deprivation on the proliferation of BLBC cell lines (MDA-MB-231/MDA-MB-468/HCC1937/BT-549) and non-BLBC cell lines (SKBR3/BT-474/MCF-7/T47D). (**D**) The cell clonal formation under different conditions of nutrient deprivation in multiple breast cancer cell lines. Bars and error flags represent the mean ± SD of at least three independent experiments. Statistically significant according to Student’s *t* test: * *p* < 0.05; ** *p* < 0.01; *** *p* < 0.001.

Basal-like breast cancer (BLBC) has extensive heterogeneity and a lack of biomarkers. Therefore, it has stronger invasiveness and a higher risk of local recurrence or a poorer outcome than other breast cancer subtypes [36]. Metabolic reprogramming is an emerging hallmark of cancer cells. Cancer cells often exhibit distinct metabolic phenotypes to promote their proliferation and progression [37]. To explore the metabolic heterogeneity of breast cancer, we used TCGA database statistical analysis. It was found that there were significant differences in the expression of metabolic enzyme genes in clinical samples of different types of breast cancer (Figure 1A). Therefore, the metabolism of [^13^C_6_]-glucose or [^13^C_5_/^15^N_2_]-glutamine was tracked using the isotope tracer method. It was found that there was increased glutamine metabolic flux in basal-like breast cancer cells (Figure 1B and Supplement Figure S1A). Additionally, specific media were used to observe the changes in breast cancer cell growth after nutrient deprivation. These results showed that the growth of basal-like breast cancer cells (MDA-MB-231/MDA-MB-468/HCC1937/BT-549) depended on glutamine metabolism, while that of non-BLBC cells (SKBR3/BT-474/MCF-7/T47D) was more dependent on glucose metabolism (Figure 1C,D and Supplement Figure S1B). Similar appearances were obtained with glutamine metabolism inhibitors (DON) and glucose metabolism inhibitors (2-DG) (Supplementary Figure S1C). The results showed that metabolism differences existed among different subtypes of breast cancer and that the growth of BLBC cells was dependent on glutamine metabolism.

### 3.2. Glutamine Deficiency Limits LIPID Metabolism in Basal-like Breast Cancer

**Figure 2:**
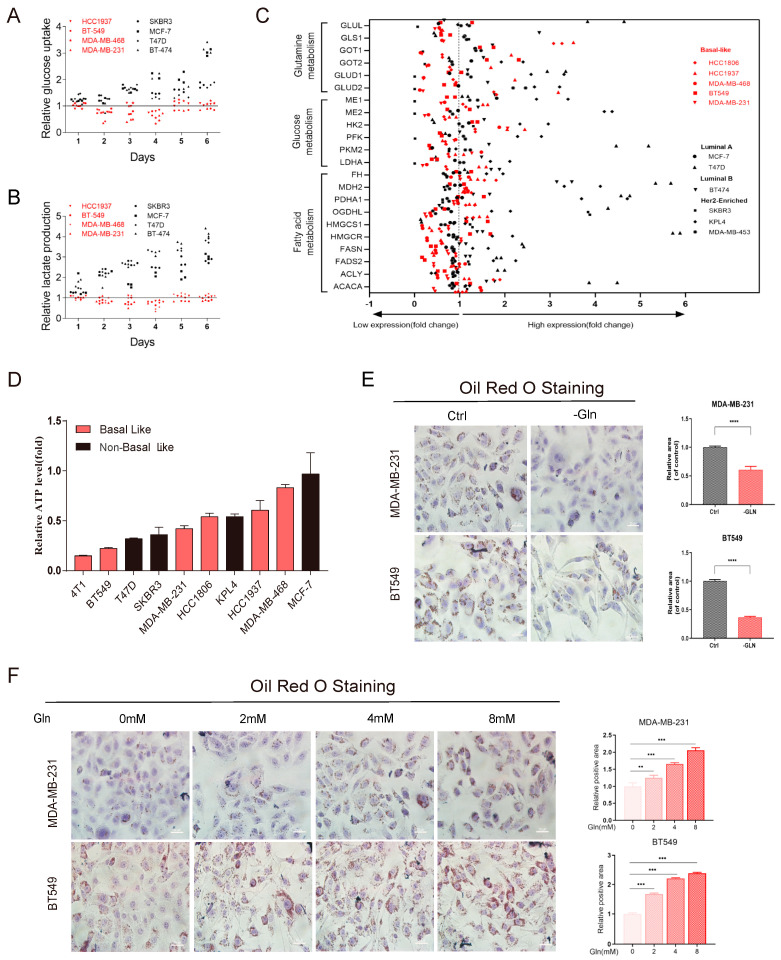
Glutamine deficiency limits lipid metabolism in basal-like breast cancer. (**A**,**B**) Effects of glutamine deprivation on the (**A**) glucose uptake and (**B**) lactate production of human breast cancer cell lines between molecular subtypes. (**C**) RT-qPCR was performed to verify the change in the mRNA expression of key enzymes of glutamine metabolism, glucose metabolism, and fatty acid metabolism in human breast cancer cell lines. (**D**) The cell metabolism level of ATP production of BLBC cell lines after glutamine deprivation for four days was detected. (**E**) Oil Red O staining was performed to verify the lipid droplet formation of glutamine-dependent breast cancer cells (MDA-MB-231 and BT549) after glutamine deprivation for 48 h. (**F**) Oil Red O staining was performed after adding glutamine (0, 2, 4, 8 mM) to glutamine-deprivation media for 48 h. Then, cells were treated with 100 μM OA for 18 h. Lipid droplets, scale bars, 20 µM. Bars and error flags represent the mean ± SD of at least three independent experiments. Statistically significant according to Student’s *t* test: ** *p* < 0.01; *** *p* < 0.001; **** *p* < 0.001.

To identify the glutamine sensitivity of different breast cancer cells, the glutamine-free medium was used to observe the metabolism of breast cancer cells with different molecular subtypes (Figure 2A,B). The results showed that glucose uptake and lactate secretion were not significantly affected for BLBC cells, but significantly increased for non-BLBC cells in glutamine-deficient conditions. This suggested that inadequate glutamine supply activated non-BLBC cell glucose metabolism. The metabolic alternations of BLBC cells in glutamine deficiency are worthy of further investigation. Hence, we further detected the mRNA expression of metabolic enzymes including glucose metabolism, glutamine metabolism, and fatty acid metabolism in breast cancer cells after glutamine deprivation. The result showed that the key enzymes of the fatty acid synthesis pathway were significantly downregulated in BLBC cells (Figure 2C).

To further clarify the role of glutamine, we detected the levels of ATP production in different molecular types of breast cancer cells after glutamine deprivation for 4 days. The result showed that inadequate glutamine could restrict energy supply in basal-like breast cancer (Figure 2D). It was rational to infer that glutamine may affect the fatty acid metabolism pathway to interfere with cell growth to a certain extent in glutamine-dependent breast cancer cells. Oil red O staining was further used to investigate the effect of glutamine on lipid levels in BLBC. We found that glutamine promotes lipid droplet formation in a concentration-dependent manner in basal-like breast cancer cells (MDA-MB-231 and BT549 cells) (Figure 2E,F). The above results indicate that glutamine can significantly affect fatty acid metabolism in basal-like breast cancer cells, and a lack of glutamine is an obstacle to fatty acid synthesis.

### 3.3. ASCT2-Mediated Glutamine Metabolism Promotes Fatty Acid Synthesis

**Figure 3:**
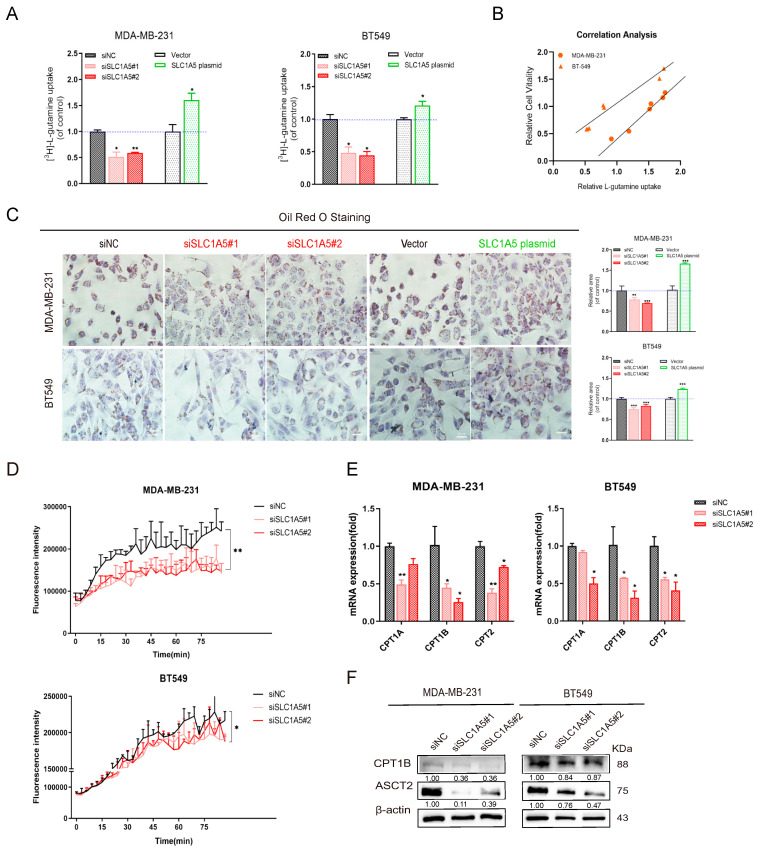
ASCT2-mediated glutamine metabolism promotes fatty acid synthesis. (**A**) Relative glutamine uptake was detected after deficiency and overexpression of SLC1A5/ASCT2. (**B**) Correlation analysis between cell growth and glutamine uptake in MDA-MB-231(R = 0.9943) and BT549 (R = 0.9835). The cell growth was detected by MTT. (**C**) Oil Red O staining was performed to verify the lipid droplet formation after deficiency and overexpression of ASCT2. (**D**) FAO rate was assessed in ASCT2- deficient MDA-MB-231 and BT549 breast cancer cells. (**E**) RT-qPCR was performed to verify the expression of genes concerning fatty acid oxidation. (**F**) Western blot analysis verified the level of protein expression of ASCT2, CPT1B, and CPT2 after deficiency and overexpression of ASCT2. Bars and error flags represent the mean ± SD of at least three independent experiments. Statistically significant according to Student’s *t* test: *n* = 3; * *p* < 0.05; ** *p* < 0.01; *** *p* < 0.001. Original western blots are presented in File S1.

ASCT2 (encoded by SLC1A5) is one of the important glutamine transporters which has been reported to transport glutamine and promote cancer cell proliferation [17]. We found that deficiency of ASCT2 inhibited glutamine uptake, and overexpression of ASCT2 promoted glutamine uptake. ASCT2 regulated the extracellular glutamine transport to affect the growth of tumor cells (Figure 3A,B and Supplement Figure S2A,B). Therefore, we used Oil red O staining to explore fatty acid metabolism level. The result show that the lipid droplets were fewer than the control in ASCT2-deficient BLBC cells, while there were more lipid droplets in ASCT2-proficient BLBC cells (Figure 3C). A similar phenomenon was found in Bodipy staining (Supplement Figure S2C). The above results indicate that lipid synthesis could be the key factor of glutamine sensibility, and ASCT2-mediated glutamine metabolism plays an indispensable role in BLBC cells.

To clarify the function of ASCT2 in regulating lipid metabolism, we further detected multiple gene expressions concerning lipid synthesis, and it can be seen that some metabolic enzymes (ACLY, ACACA, HMGCR, etc.) were downregulated in ASCT2-deficient BLBC cells. These genes were significantly upregulated after overexpression of ASCT2 (Supplement Figure S2D). Subsequently, we observed that the fatty acid oxidation rate decreased after the deficiency of ASCT2 in BLBC cells (Figure 3D). The fatty acid can be transported into mitochondria through carnitine palmityl transferase (CPT) and produce energy by oxidation. Next, it was found that ASCT2 deficiency significantly downregulated CPT1B and CPT2 mRNA and protein expression (Figure 3E,F). Moreover, our results show that supplementation of glutamine downstream products (α-KG and NAC) can salvage the decreased CPT1B expression in ASCT2-deficient conditions (Supplement Figure S2E). This research provides evidence of a correlation between glutamine metabolism and lipid synthesis.

### 3.4. ASCT2 Regulates PPARα to Promote Fatty Acid Synthesis

**Figure 4:**
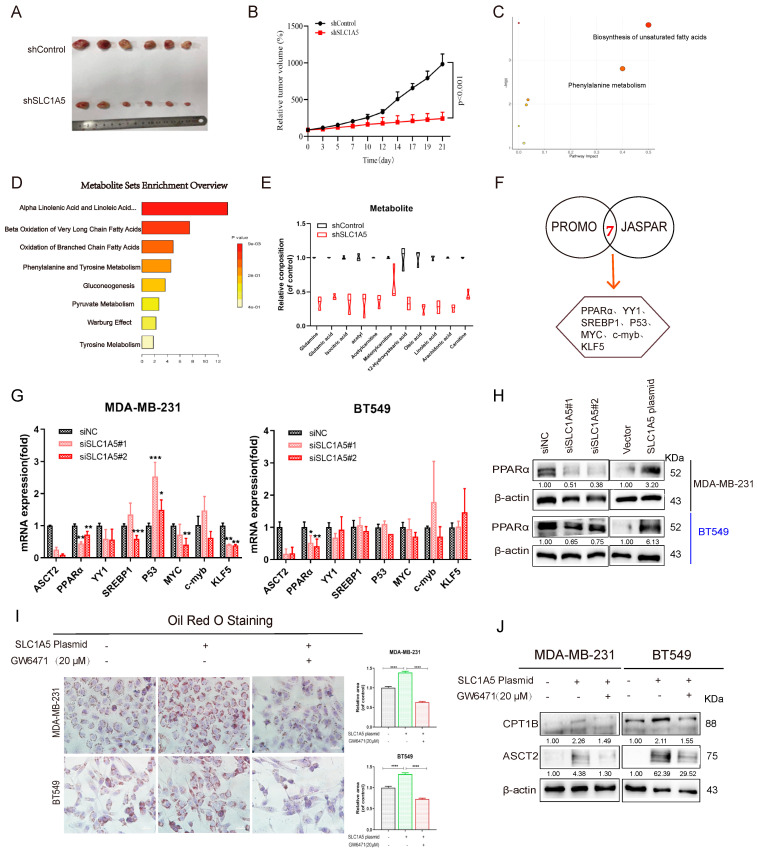
ASCT2 regulates PPARα to promote fatty acid synthesis. (**A**,**B**) The effects of ASCT2 depletion on the tumor volumes of MDA-MB-231 xenografts in nude mice. (**C**,**D**) Pathway enrichment analysis using pathway-associated metabolite sets (SMPDB) was shown to predict potential pathways. (**E**) Metabolomics measured levels of fatty acid-associated metabolites from tumor tissue. (**F**) Bioinformatic prediction tools, including PROMO and JASPAR, were used to predict the transcription factors that regulate lipid metabolism. (**G**) RT-qPCR was performed to verify the change in mRNA expression of seven transcription factors in ASCT2-deficient MDA-MB-231 and BT549 cells. (**H**) Western blot was performed to verify the expression levels of PPARα. (**I**) MDA-MB-231 and BT549 cells with overexpression of ASCT2 were exposed to GW6471 for 24 h. Oil Red O staining was then detected. Scale bars, 20 µm. (**J**) Western blot was performed to verify the fatty acid oxidation-related protein expression of CPT1B. Bars and error flags represent the mean ± SD of at least three independent experiments. Statistically significant according to Student’s *t* test: *n* = 3; * *p* < 0.05; ** *p* < 0.01; *** *p* < 0.001; **** *p* < 0.01. Original western blots are presented in File S1.

To further confirm the important role of ASCT2 in tumor metabolism, we constructed a stable deficiency of ASCT2 in MDA-MB-231 cells. These cells were subcutaneously injected into the flanks of mice for 21 days. It can be seen that the tumor growth of the shSLC1A5 group was inhibited compared to the control group (Figure 4A,B). Tumor tissue samples in the control group and ASCT2-deficient group were analyzed using the metabolomic method. The main differential metabolic pathways that had the higher rankings included the biosynthesis of unsaturated fatty acid and beta-oxidation of very-long-chain fatty acid pathways (Figure 4C,D). Additionally, the different potential metabolites showed that fatty acids and intermediate products were significantly decreased in the ASCT2-deficient group(Figure 4E). At the same time, we also detected a significant decrease in fatty acid-related proteins after knocking down ASCT2 (Supplement Figure S2F,G). The results also suggest that ASCT2 may regulate fatty acid synthesis pathways to affect tumor growth in vivo.

To determine the mechanism of ASCT2 regulating lipid metabolism in basal-like breast cancer, the UCSC database (http://genome.ucsc.edu/, accessed on 3 February 2023) was utilized to determine the promoter sequence of enzymes (ACLY, HMGCR, CPT1B), and then the putative TFs binding to them were predicted by PROMO and JASPAR. Among the TFs predicted by both databases, seven transcription factors, including PPARα, YY1, SREBP1, p53, MYC, c-myb, and KLF5, were selected through reports (Figure 4F). To discover which TF was mainly exerted downstream of ASCT2, RT-qPCR was performed to detect the change in mRNA expression. Interestingly, the results showed that only PPARα was downregulated after deleting ASCT2, while PPARα was upregulated after ASCT2 overexpression (Figure 4G,H). To assess PPARα as a mediator regulating lipid metabolism, it can be seen that the PPARα antagonist GW6471 reversed the increase in lipid droplets induced by overexpression of ASCT2 (Figure 4I). Additionally, GW6471 also inhibited the upregulation of CPT1B protein expression in BLBC cells when ASCT2 was overexpressed (Figure 4J). These results imply that ASCT2 mediates the transcription factor PPARα to regulate lipid metabolism in BLBC cells.

### 3.5. C118P Regulates Lipid Metabolism Targeting ASCT2

**Figure 5:**
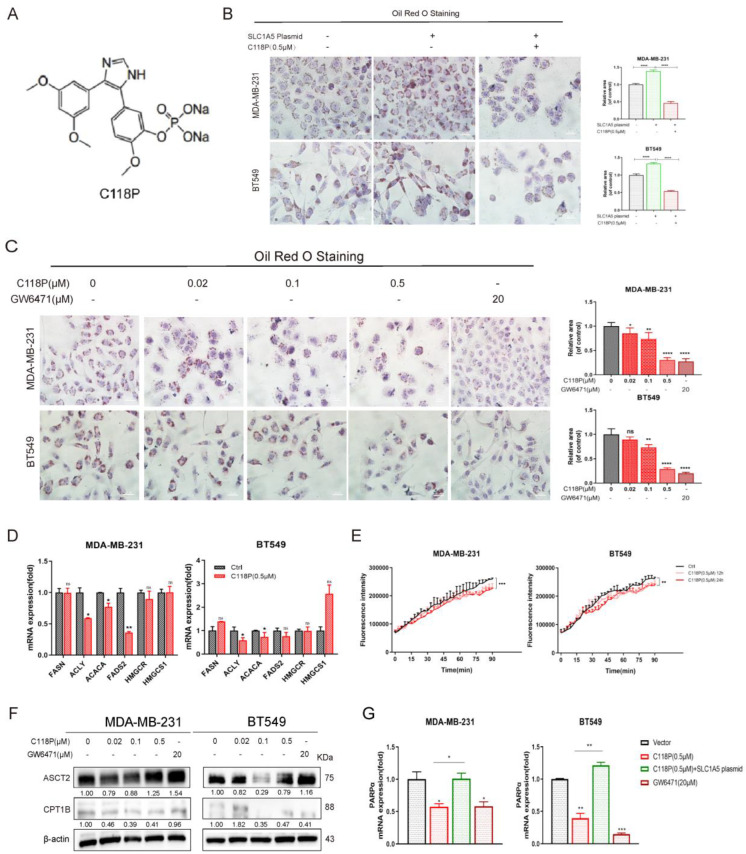
C118P regulates lipid metabolism by targeting ASCT2. (**A**) The chemical construction of C118P. (**B**) MDA-MB-231 and BT549 cells with ASCT2 overexpression were exposed to C118P for 24 h, and Oil Red O staining was then detected. Lipid droplets. Scale bars, 20 µm. (**C**) Oil Red O staining was performed to verify the lipid droplet formation treated with C118P for 24 h, with GW6471 as a positive control. Lipid droplets. Scale bars, 20 µm. (**D**) The mRNA expression of FA synthesis key enzymes was performed in MDA-MB-231 and BT549 treated with C118P and GW6471 for 24 h. (**E**) FAO activity assay was detected in BLBC cells treated with C118P for 12 h and 24 h. (**F**) Western blot verified the FAO-associated protein expression after treatment with C118P and GW6471 for 48 h. (**G**) RT-qPCR was then performed to assess the change in mRNA expression of PPARα. Bars and error flags represent the mean ± SD of at least three independent experiments. Statistically significant according to Student’s *t* test: *n* = 3; * *p* < 0.05; ** *p* < 0.01; *** *p* < 0.001; **** *p* < 0.0001. Original western blots are presented in File S1.

Previous findings of the research group showed that C118P has excellent anti-breast cancer effects and can directly bind to the protein to promote the degradation of ASCT2 [38]. However, the potential anti-breast-cancer mechanisms of C118P (Figure 5A) targeting ASCT2 remain unknown. We found that C118P inhibited lipid droplet formation, targeting ASCT2 (Figure 5B,C), and downregulated key enzymes of FA synthesis, including ACACA, ACLY, and FADS2 (Figure 5D). This suggests that C118P can significantly inhibit fatty acid synthesis in BLBC cells by targeting ASCT2.

To further verify the effects of C118P on fatty acid metabolism, the variation in fatty acid oxidation rates at different times of drug action was also detected. The results showed that the FAO rate remarkably decreased when treated with C118P for 12 h or 24 h in BLBC cells (Figure 5E). And C118P inhibited fatty acid-associated protein expression of CPT1B (Figure 5F). Furthermore, C118P downregulated mRNA expression of PPARα, while overexpression of ASCT2 reversed this phenomenon (Figure 5G). Above all, the small-molecule inhibitor C118P could target ASCT2 to restrain lipid metabolism in basal-like breast cancer cells.

### 3.6. C118P Inhibits Basal-like Breast Cancer Progress In Vivo

**Figure 6:**
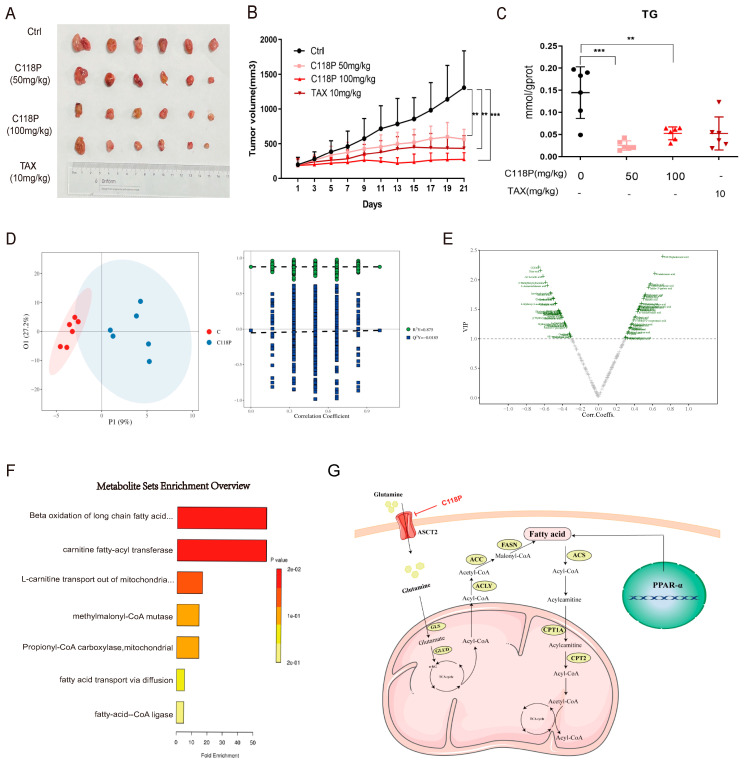
C118P inhibits basal-like breast cancer progress in vivo. (**A**) The mice were sacrificed and the tumor tissues were photographed after the 21st day of treatment. (**B**) The tumor volumes were recorded during the 21 days. (**C**) Triglyceride assay kit was utilized to detect triglycerides in tumor tissues. (**D**) The OPLS-DA model was employed to perform metabolic profiles of the individuals from the two predefined groups (R^2^Y = 0.875, Q^2^Y = 0.0185). (**E**) Volcano plot analyzing the differential metabolites from the control group and C118P group in vivo is shown. (**F**) Pathway enrichment analysis using pathway-associated metabolite sets (SMPDB) was shown to predict potential pathways. (**G**) Schematic representation of ASCT2-mediated lipid metabolism mechanisms in basal-like breast cancer. Data are presented as the mean ± SD; statistical significance was assessed using the unpaired *t*-test. ** *p* < 0.01; *** *p* < 0.001. *n* = 6.

To further assess the inhibitory effect of C118P on BLBC growth in vivo, a nude mouse breast cancer xenograft tumor model was established by inoculating MDA-MB-231 cells. When the tumor volume grew to 100 mm^3^, C118P was injected into the tail vein at concentrations of 50 mg/kg and 100 mg/kg. It can be seen that the tumor’s growth was inhibited when treated with C118P (Figure 6A,B). And the inhibitory effect of C118P on tumor proliferation was comparable to paclitaxel (10 mg/kg). Next, we detected the triglyceride (TG) in tumor tissues. The result showed that C118P can inhibit lipid synthesis in vivo (Figure 6C). The full-targeted metabolomic method analyzed the metabolic characteristics of the control group and the C118P administration group. The main differential metabolic pathways with the highest ranking were the long-chain fatty acid oxidation pathway and fatty acid transport (Figure 6D–F). These results further confirmed that C118P can affect lipid metabolism to inhibit BLBC growth in vivo.

## 4. Discussion

There are significant differences in glutamine dependence among different molecular types of breast cancer. Glutamine provides the nitrogen and carbon sources needed to support cancer cell proliferation and metastasis. Many studies have shown that ER^+^ breast cancer cell lines are less dependent on glutamine than triple-negative breast cancer [5,6,7]. In luminal breast cancer cells, the transcription factor GATA3 activates the expression of glutamine synthase (GLUL) to facilitate glutamine production, thereby reducing the dependence on extracellular glutamine [25]. In addition, some oncogenes, such as MYC and KRAS, have also been shown to have the ability to alter glutamine metabolism [39]. However, the reasons for the heterogeneity of glutamine metabolism are still inconclusive. Our results showed that basal-like breast cancer growth is more dependent on glutamine compared to other subtypes of breast cancer. The reason is that the change in glutamine affects the lipid metabolism. As cell metabolism is relatively complex, the effect of glutamine on fatty acid synthesis may be only one part. The impact of the transformation between the two needs to be further explored in the future.

Recent studies have shown that PPARα is an important sensor and regulator of lipids and plays an important role in regulating the expression of various genes related to glucose and lipid metabolism homeostasis, adipogenesis, and inflammation [40,41]. PPARα is known to regulate cellular metabolism. However, the researchers noted that cellular metabolism also affects PPARα activity [42,43]. In this study, we found that ASCT2-mediated upregulation of lipid synthesis may be related to the regulation of PPARα. In addition, CPT1B, as a target gene regulated downstream of PPARα, plays an important role in regulating the rate of FAO [44,45]. Our results show that the addition of a PPARα antagonist can reverse the upregulation of CPT1B caused by ASCT2 overexpression, further regulating the lipid metabolism pathway. Our study demonstrated that ASCT2 regulation of PPARα affects fatty acid metabolism. This research will open new avenues for the study of tumor metabolism. However, whether glutamine plays a direct role remains to be explored.

Our previous findings have verified that C118P can bind to ASCT2 and inhibit the expression of protein [38]. Further research has shown that it can inhibit lipid synthesis and metabolism by targeting ASCT2 in basal-like breast cancer. In addition, C118P can also significantly downregulate the gene expression of the transcription factor PPARα. These results indicate that C118P regulates ASCT2 to affect PPARα and downregulate the lipid metabolism pathway. However, the effect of C118P in inhibiting the lipid metabolism pathway may also be exerted through other pathways or targets. The mechanism of ASCT2-mediated C118P regulating the lipid metabolism pathway in breast cancer cells has not been thoroughly studied and needs to be further explored.

## 5. Conclusions

In summary, we found that basal-like breast cancer is more dependent on glutamine metabolism, and differences in lipid synthesis capacity may be one of the key factors in glutamine addiction. PPARα mediates the regulation of the downstream lipid metabolism pathway via ASCT2 to affect breast cancer growth (Figure 6G). Additionally, C118P can regulate the expression of PPARα to inhibit the lipid metabolism pathway and the growth of basal-like breast cancer by targeting ASCT2. This study provides new insights into the metabolic heterogeneity of breast cancer and the mechanism of ASCT2 in promoting tumor proliferation. Also, it provides an important theoretical basis for the development of ASCT2 inhibitors.

## Data Availability

All relevant data are within the paper and its Supplementary Materials. The data that support the findings of this study are available from the corresponding author upon reasonable request.

## Supplementary Materials

The following supporting information can be downloaded at: https://www.mdpi.com/article/10.3390/cancers16173028/s1, Supplement Figure S1. Glutamine dependence analysis in different breast cancer cells (A) The heatmap depicted representative isotope-labeled metabolites. Different metabolites are in rows and cells in columns (*n* = 3). (B) The statistical chart of cell clonal formation under different conditions of nutrient deprivation. (C) The effect of glutamine metabolism inhibitors (DON) and glucose metabolism inhibitors (2-DG) on the proliferation of breast cancer cell lines. Bars and error flags represent the mean ± SD of at least three independent experiments; statistically significant by Student *t* test; * *p* < 0.05; ** *p* < 0.01; *** *p* < 0.001; **** *p* < 0.0001. Supplement Figure S2. Effects of ASCT2 on the fatty acid metabolism in BLBC (A,B) The cell growth curve after knockdown and overexpression ASCT2 in MDA-MB-231 and BT549 was detected by MTT. (C) Bodipy staining (Scale bars, 50 µm) was performed to verify the lipid level of MDA-MB-231 and BT549 after deficiency and overexpression of SLC1A5. Blue represents DAPI staining, and green represents Bodipy staining. (D) RT-qPCR was performed on the mRNA expression of FA synthesis key enzymes after deficiency and overexpression of ASCT2. (E) The protein expression of supplementation of α-KG(α-ketoglutarate) and NAC(Acetylcysteine) in ASCT2-deficient conditions. (F) The tumor growth of ASCT2 depletion on MDA-MB-231 xenografts in nude mice. (G) The tumor tissues were further detected by hematoxylin and eosin (H&E) staining and ASCT2 expression was detected in tumors. Scale bars, 50 µM. Bars and error flags represent the mean ± SD of at least three independent experiments; statistically significant by Student *t* test; * *p* < 0.05; ** *p* < 0.01; *** *p* < 0.001. File S1: Original western blots.
