# Supplementary material for: ASCT2 Regulates Fatty Acid Metabolism to Trigger Glutamine Addiction in Basal-like Breast Cancer
Source: Cancers (Basel). 2024 Aug 30;16(17):3028. doi: 10.3390/cancers16173028 (PMC11394221; doi:10.3390/cancers16173028)
Supplement: Supplementary file 1 [file cancers-16-03028-s001.zip › cancers-3181661-supplementary.pdf]

# Supplementary Materials:

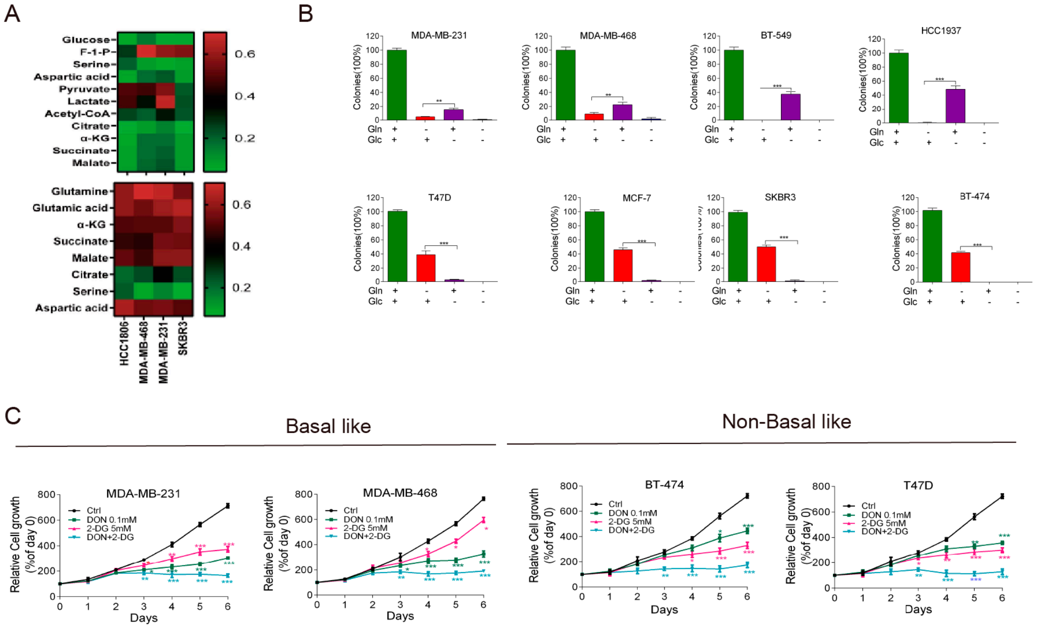


**Supplement Figure S1 Glutamine dependence analysis in different breast cancer cells**

(A)The heatmap depicted representative isotope-labeled metabolites. Different metabolites are in rows and cells in columns (n=3). (B)The statistical chart of cell clonal formation under different conditions of nutrient deprivation. (C)The effect of glutamine metabolism inhibitors (DON) and glucose metabolism inhibitors (2-DG) on the proliferation of breast cancer cell lines. Bars and error flags represent the mean ± SD of at least three independent experiments; statistically significant by Student t test; $*P<0.05$; $**P<0.01$; $***P<0.001$; $****P<0.0001$.

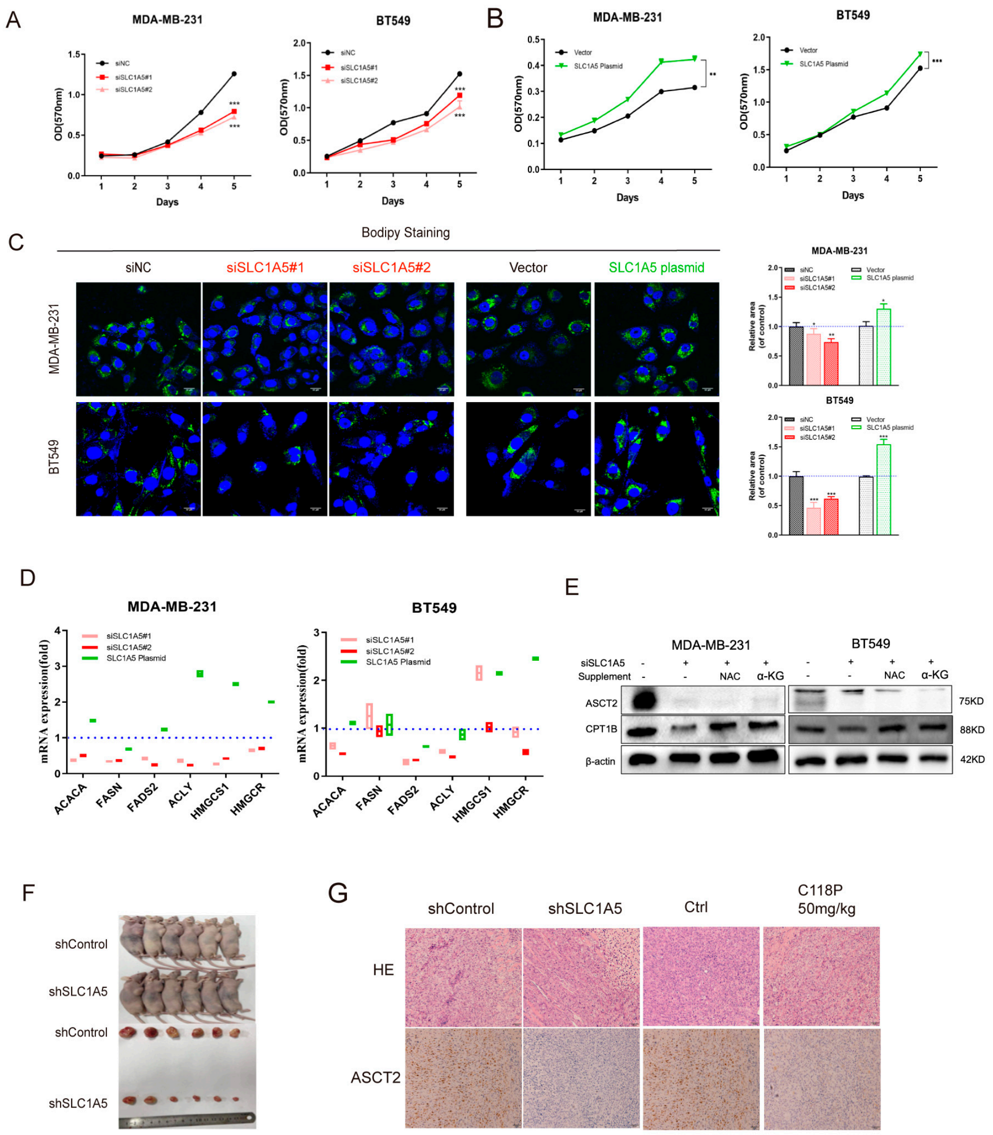


**Supplement Figure S2 Effects of ASCT2 on the fatty acid metabolism in BLBC**

(A-B) The cell growth curve after knockdown and overexpression ASCT2 in MDA-MB-231 and BT549 was detected by MTT. (C) Bodipy staining (Scale bars, 50 μm) was performed to verify the lipid level of MDA-MB-231 and BT549 after deficiency and overexpression of SLC1A5. Blue represents DAPI staining, and green represents Bodipy staining. (D) RT-qPCR was performed on the mRNA expression of FA synthesis key enzymes after deficiency and overexpression of ASCT2. (E) The protein expression of supplementation of α-KG(α-ketoglutarate) and NAC(Acetylcysteine) in ASCT2-deficient conditions. (F) The tumor growth of ASCT2 depletion on MDA-MB-231 xenografts in nude mice. (G) The tumor tissues were further detected by hematoxylin and eosin (H&E) staining and ASCT2 expression was detected in tumors. Scale bars, 50 μM. Bars and error flags represent the mean ± SD of at least three independent experiments; statistically significant by Student t test; $*P<0.05$; $**P<0.01$; $***P<0.001$.
