# Supplementary figures and images for: ASCT2 Regulates Fatty Acid Metabolism to Trigger Glutamine Addiction in Basal-like Breast Cancer
Source: Cancers (Basel). 2024 Aug 30;16(17):3028. doi: 10.3390/cancers16173028 (PMC11394221; doi:10.3390/cancers16173028)

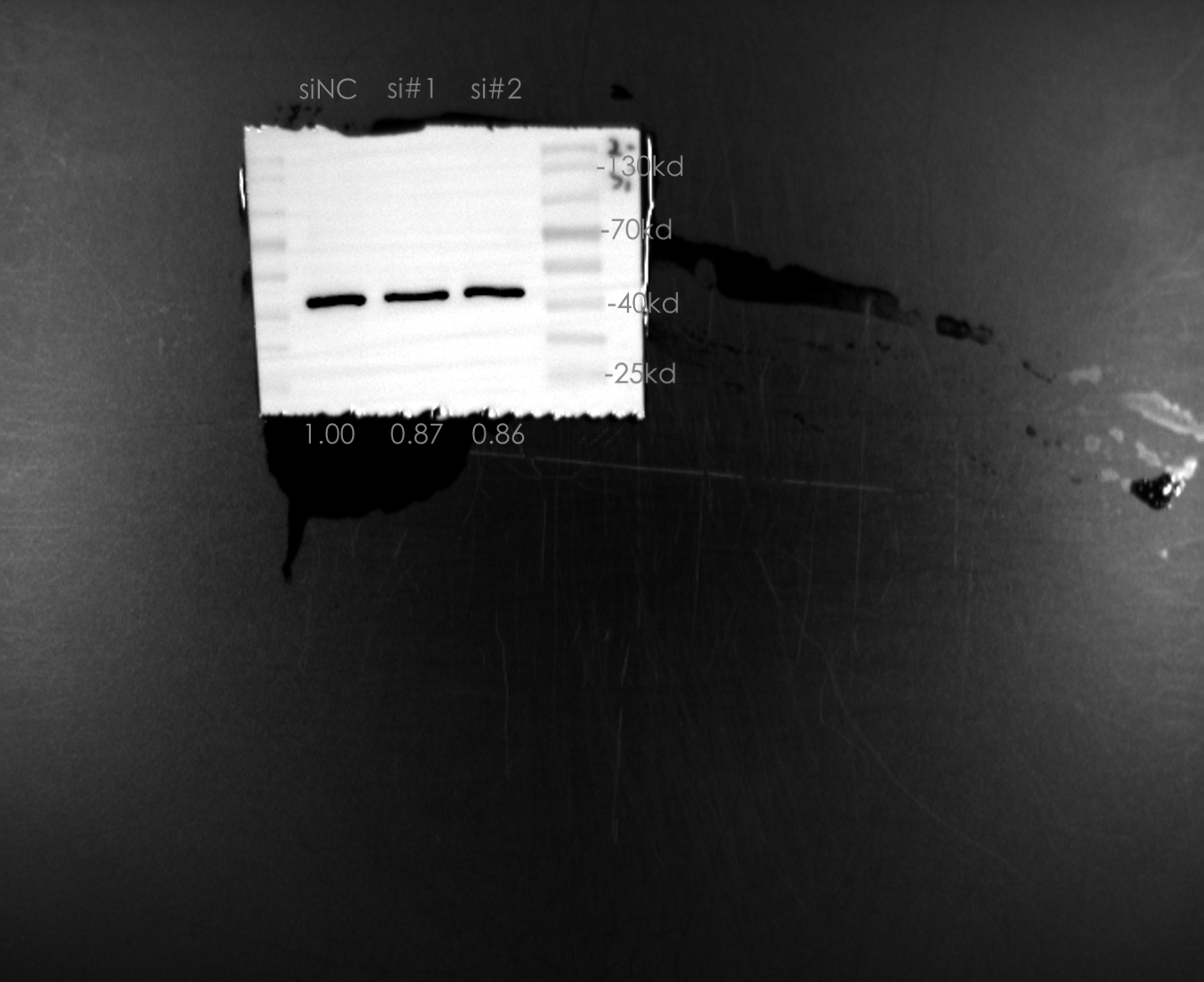

Supplement: Supplementary file 1 [file cancers-16-03028-s001.zip › File S1/For Figure 3/231-si-ACTIN-M.Tif]

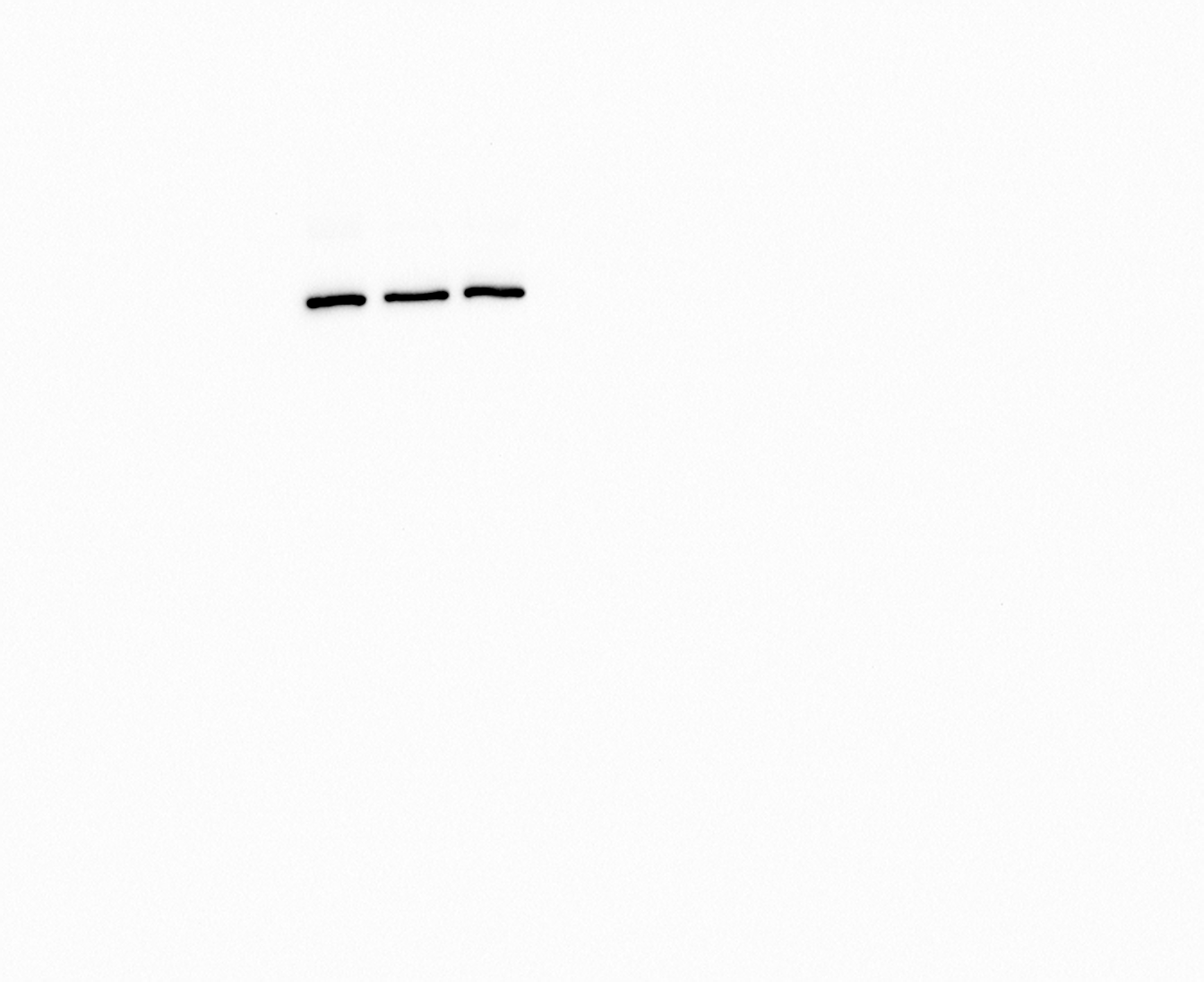

Supplement: Supplementary file 1 [file cancers-16-03028-s001.zip › File S1/For Figure 3/231-si-ACTIN.Tif]

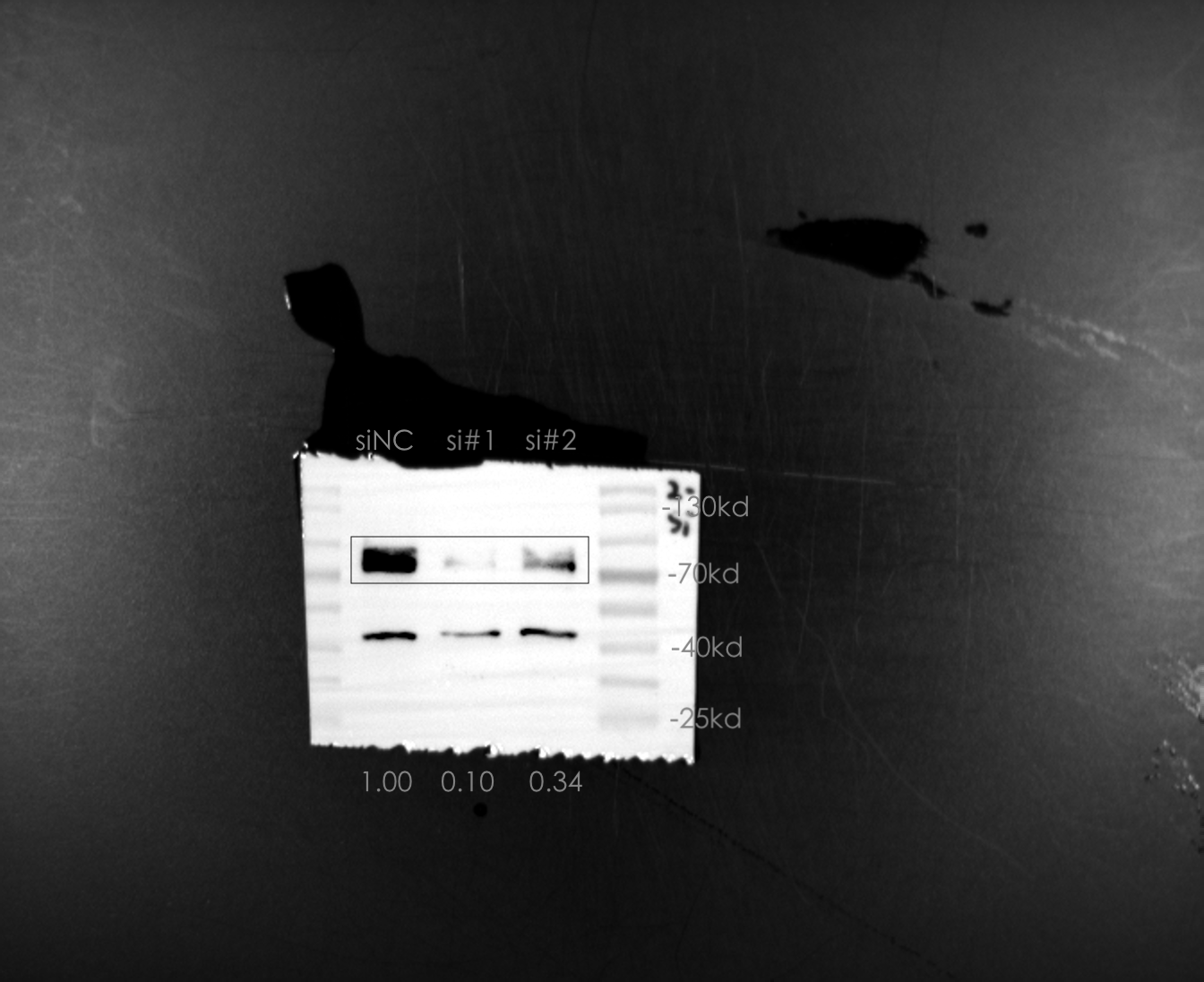

Supplement: Supplementary file 1 [file cancers-16-03028-s001.zip › File S1/For Figure 3/231-si-ASCT2-M.Tif]

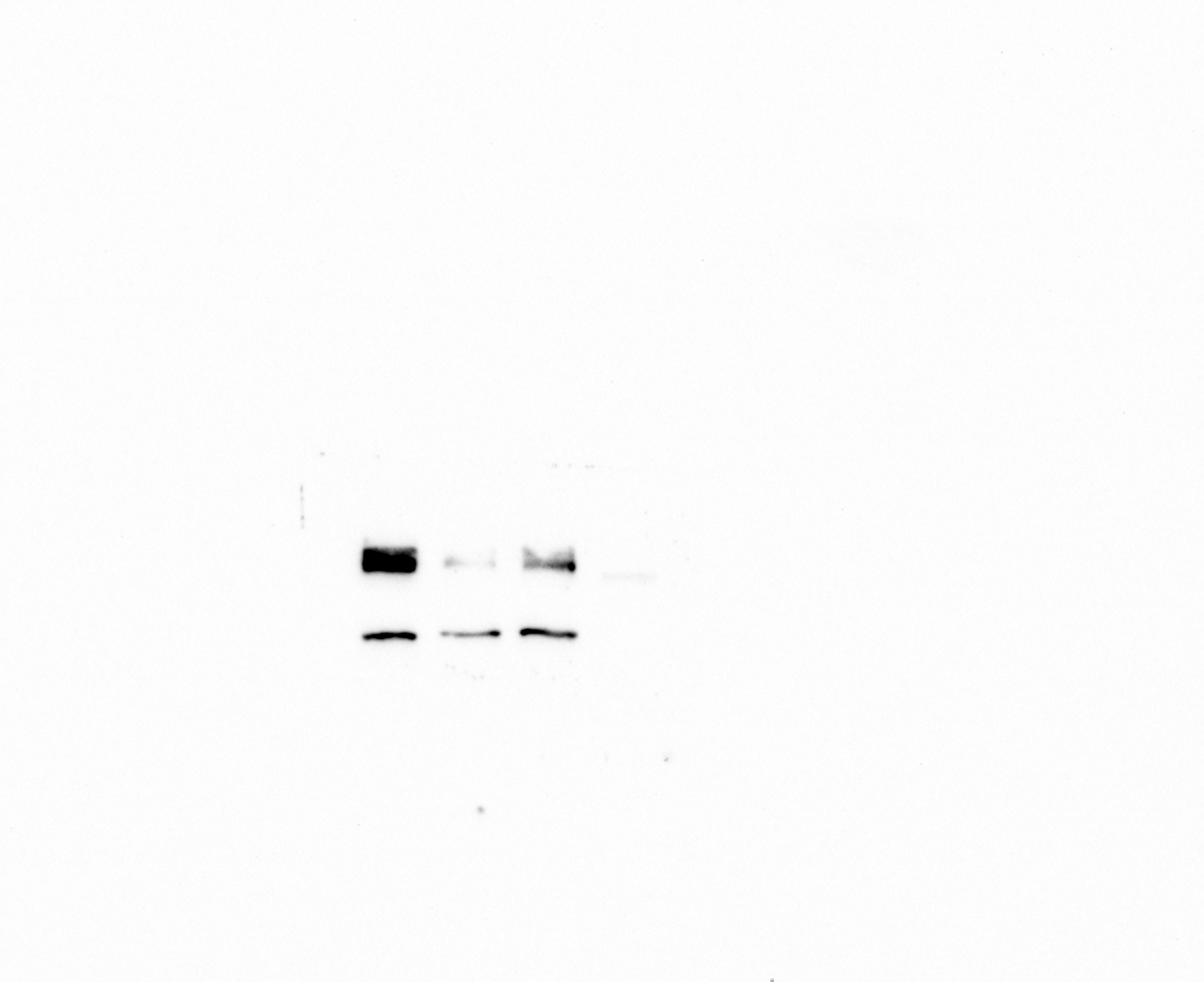

Supplement: Supplementary file 1 [file cancers-16-03028-s001.zip › File S1/For Figure 3/231-si-ASCT2.Tif]

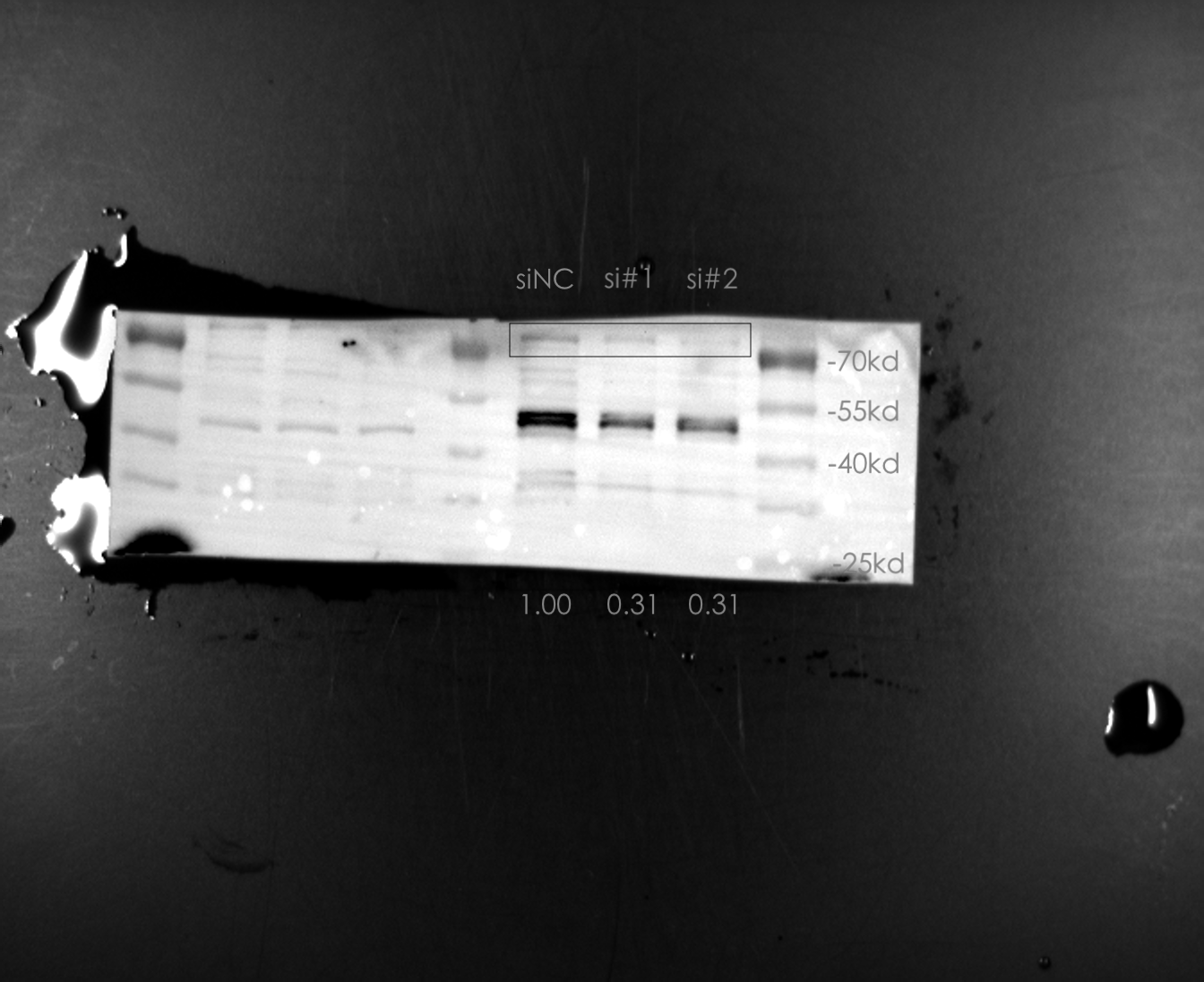

Supplement: Supplementary file 1 [file cancers-16-03028-s001.zip › File S1/For Figure 3/231-si-CPT1B-M.Tif]

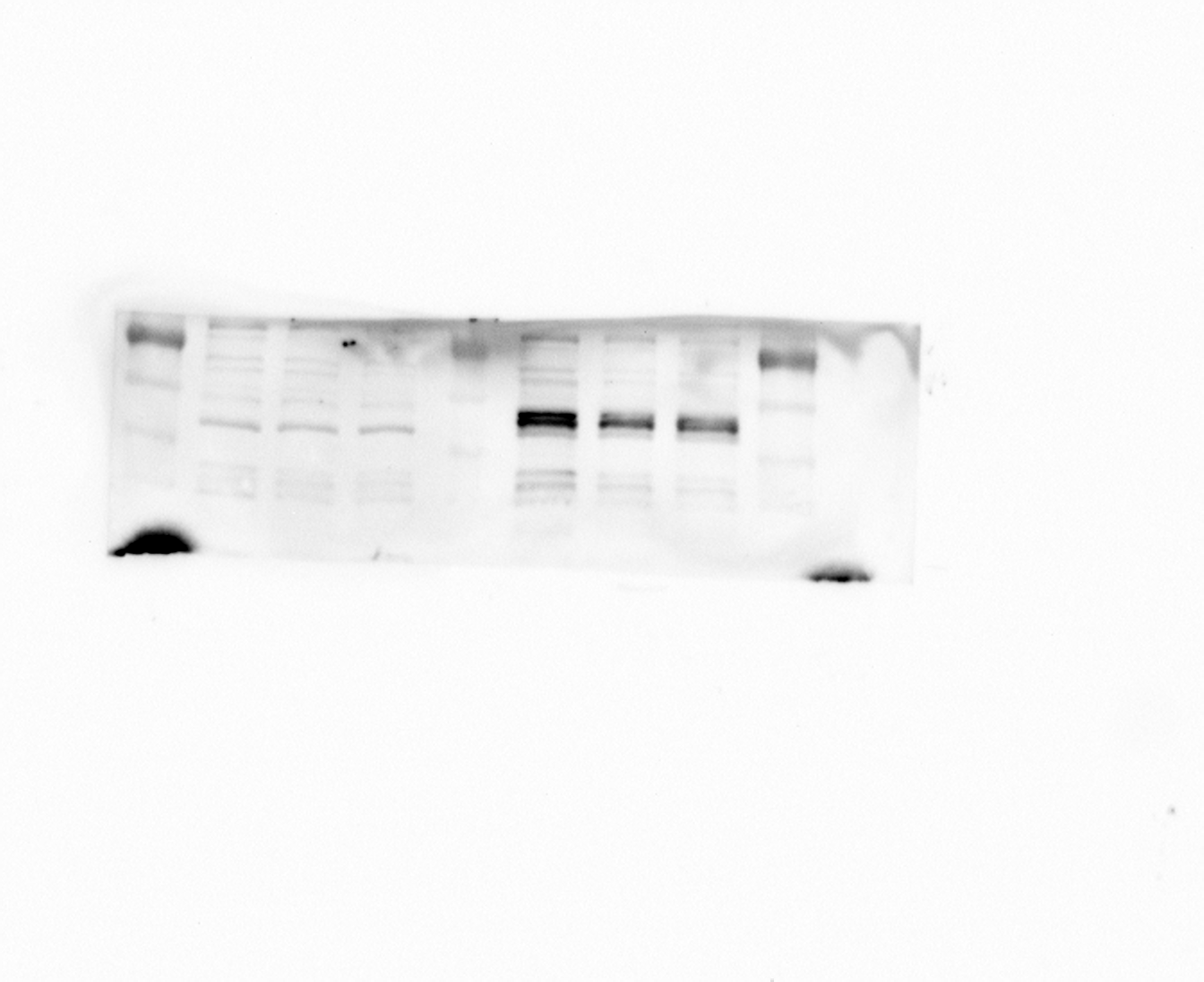

Supplement: Supplementary file 1 [file cancers-16-03028-s001.zip › File S1/For Figure 3/231-si-CPT1B.Tif]

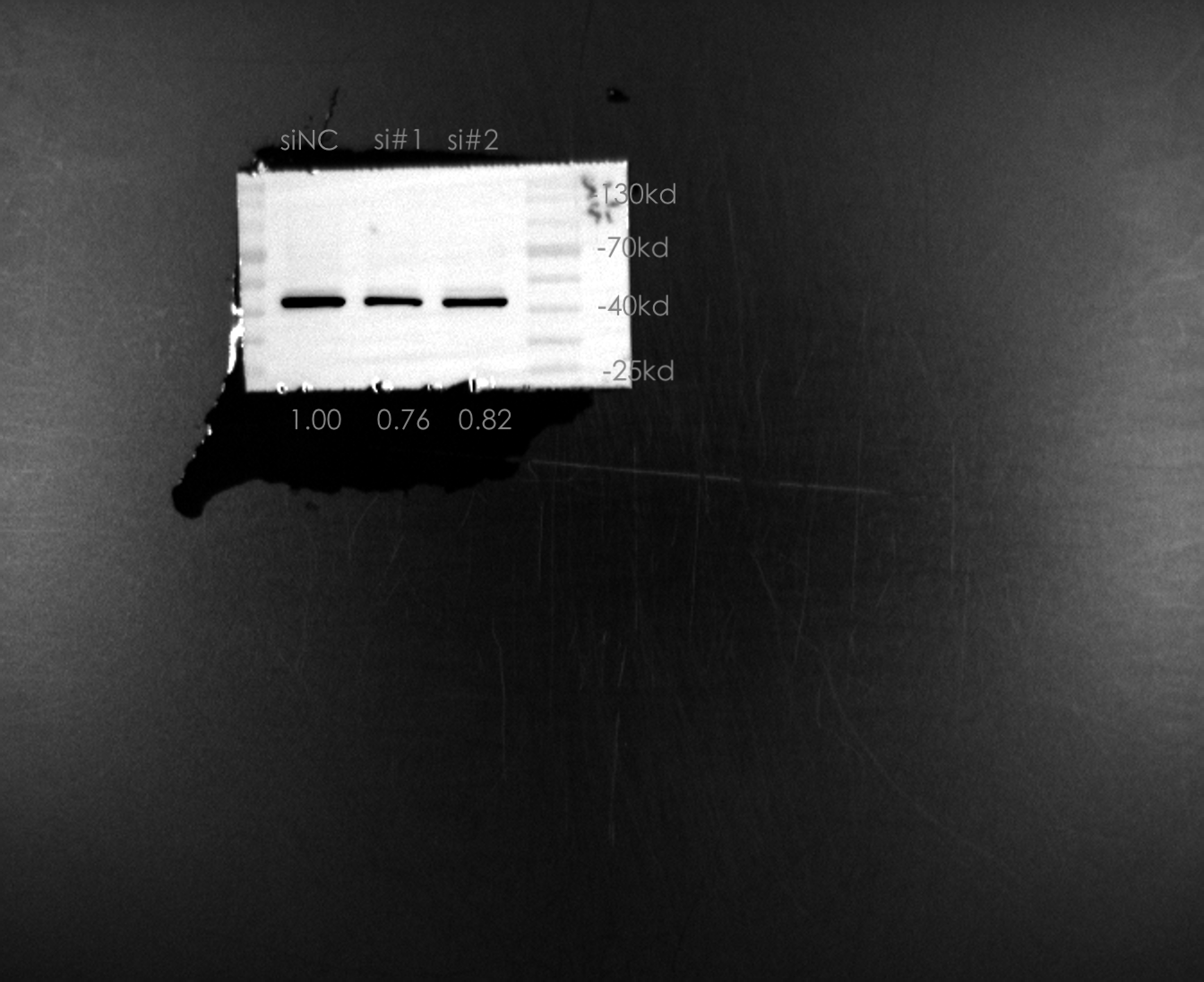

Supplement: Supplementary file 1 [file cancers-16-03028-s001.zip › File S1/For Figure 3/549-si-ACTIN-M.Tif]

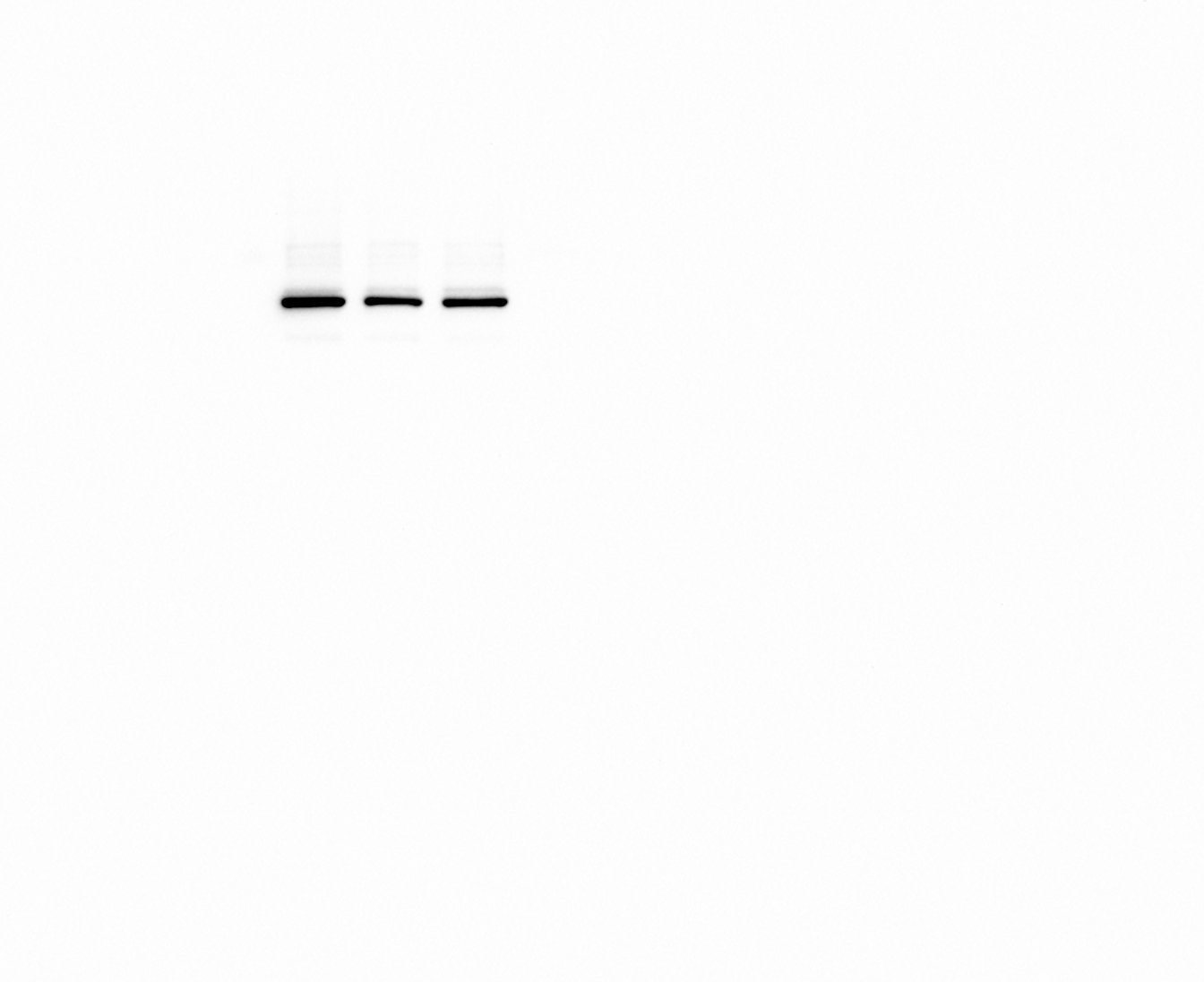

Supplement: Supplementary file 1 [file cancers-16-03028-s001.zip › File S1/For Figure 3/549-si-ACTIN.Tif]

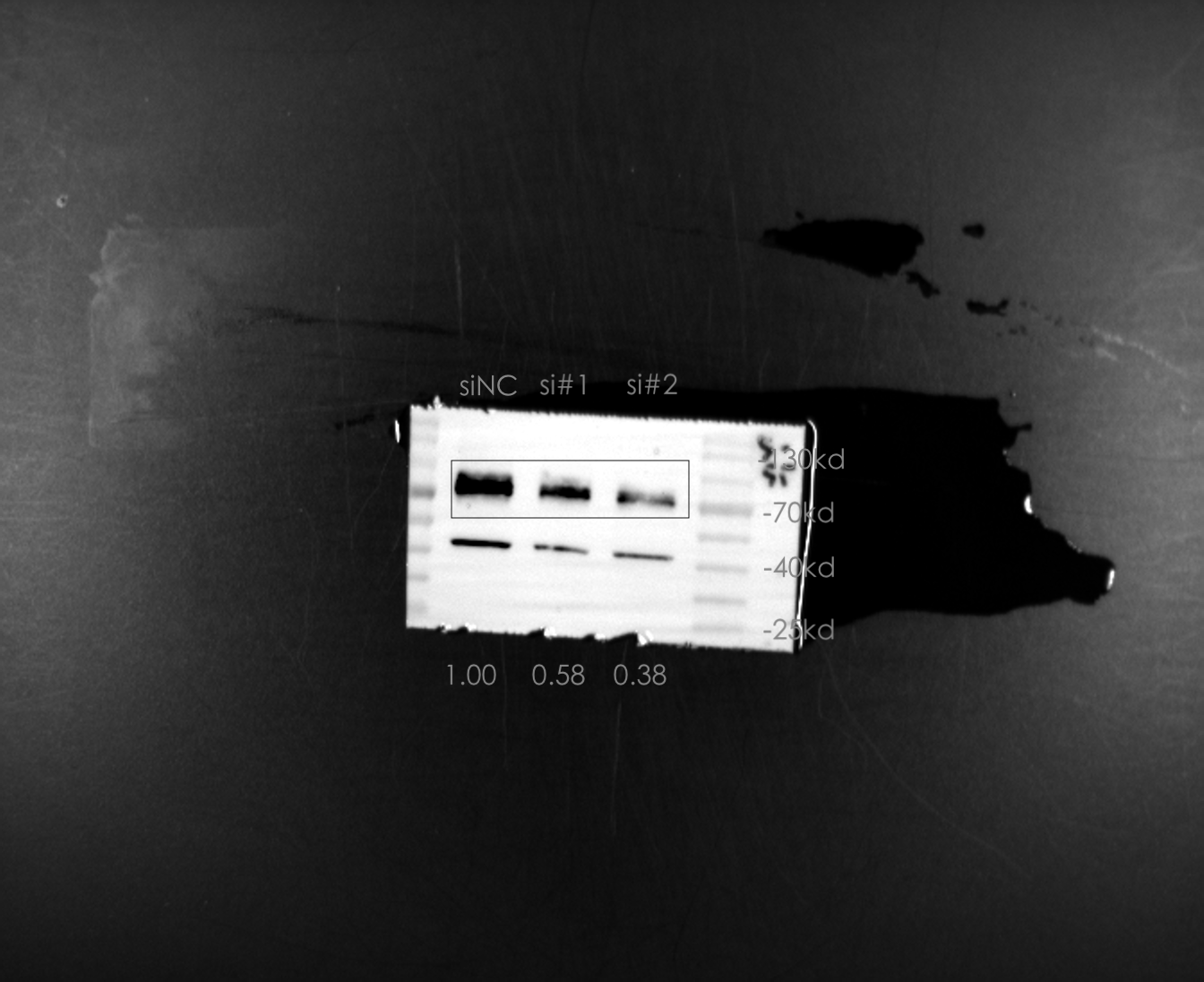

Supplement: Supplementary file 1 [file cancers-16-03028-s001.zip › File S1/For Figure 3/549-si-ASCT2-M.Tif]

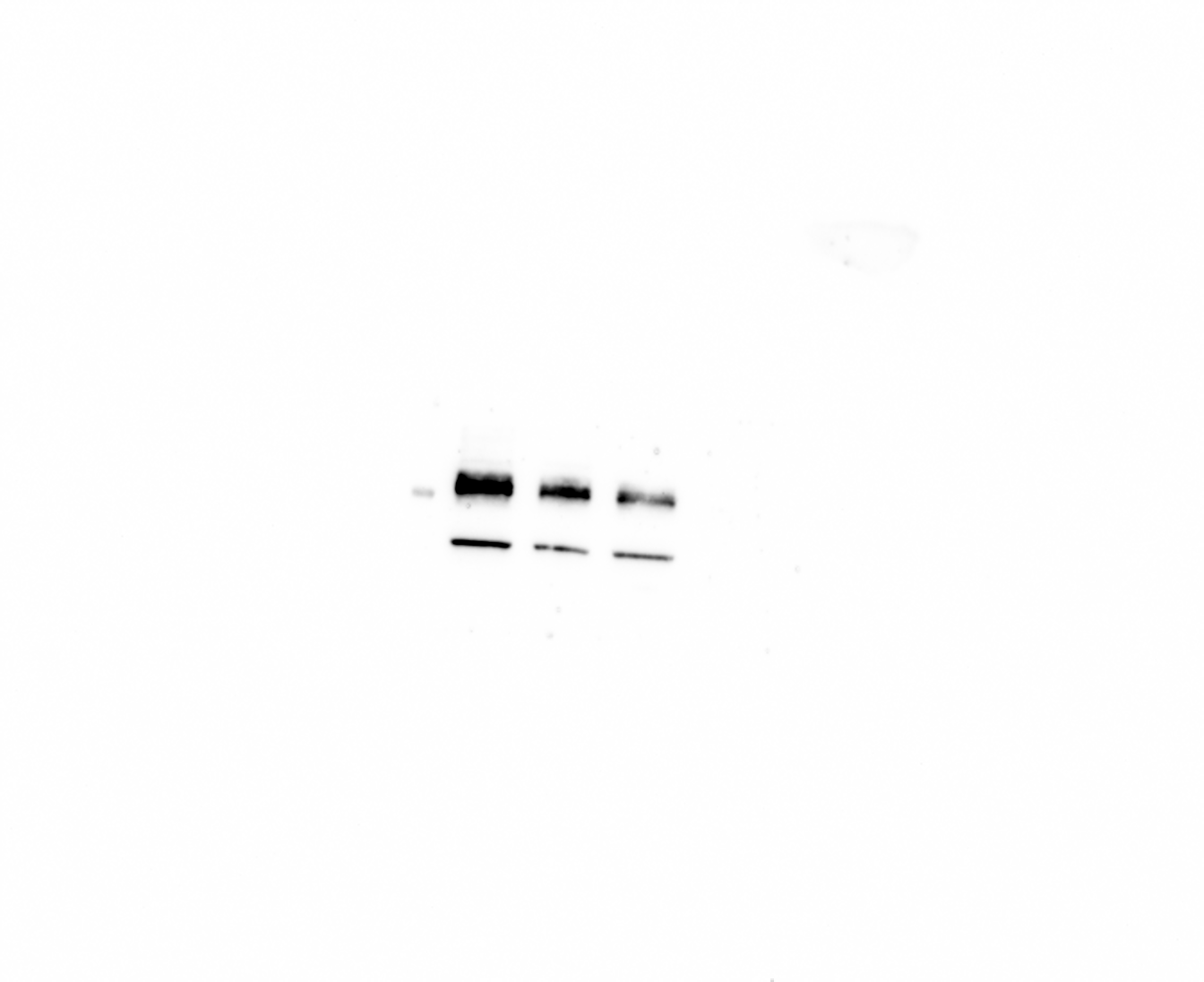

Supplement: Supplementary file 1 [file cancers-16-03028-s001.zip › File S1/For Figure 3/549-si-ASCT2.Tif]

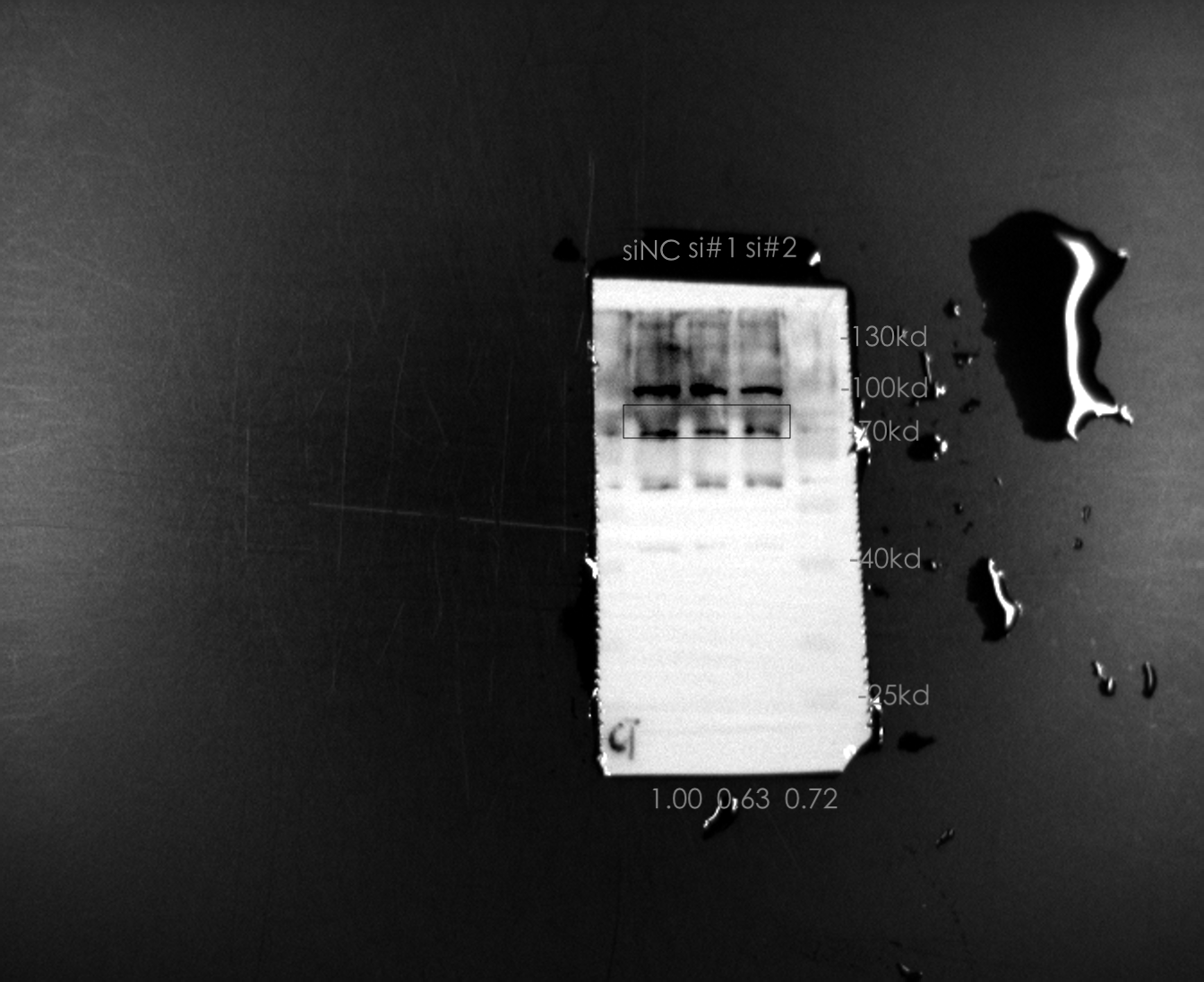

Supplement: Supplementary file 1 [file cancers-16-03028-s001.zip › File S1/For Figure 3/549-si-CBT1B-M.Tif]

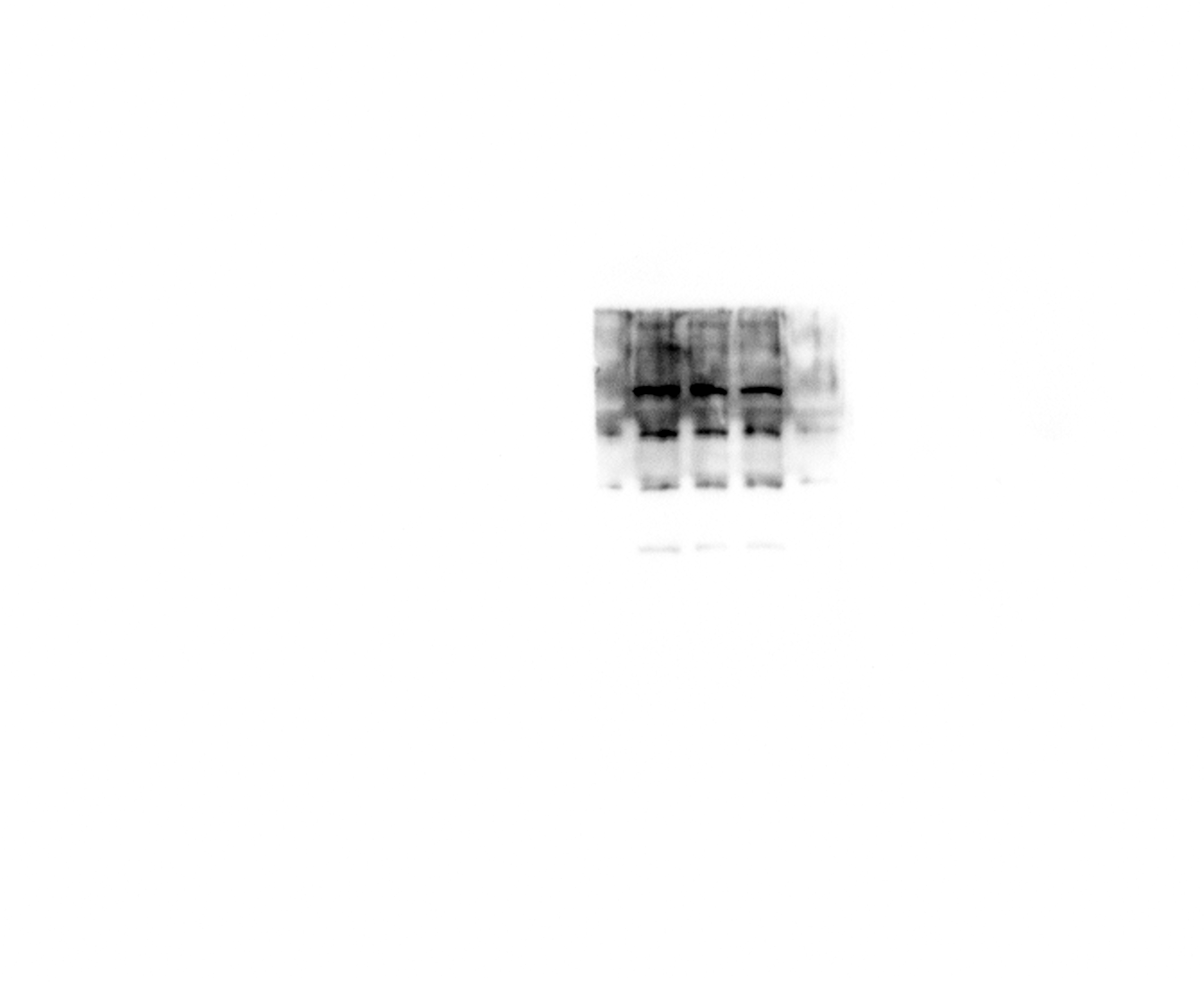

Supplement: Supplementary file 1 [file cancers-16-03028-s001.zip › File S1/For Figure 3/549-si-CBT1B.Tif]

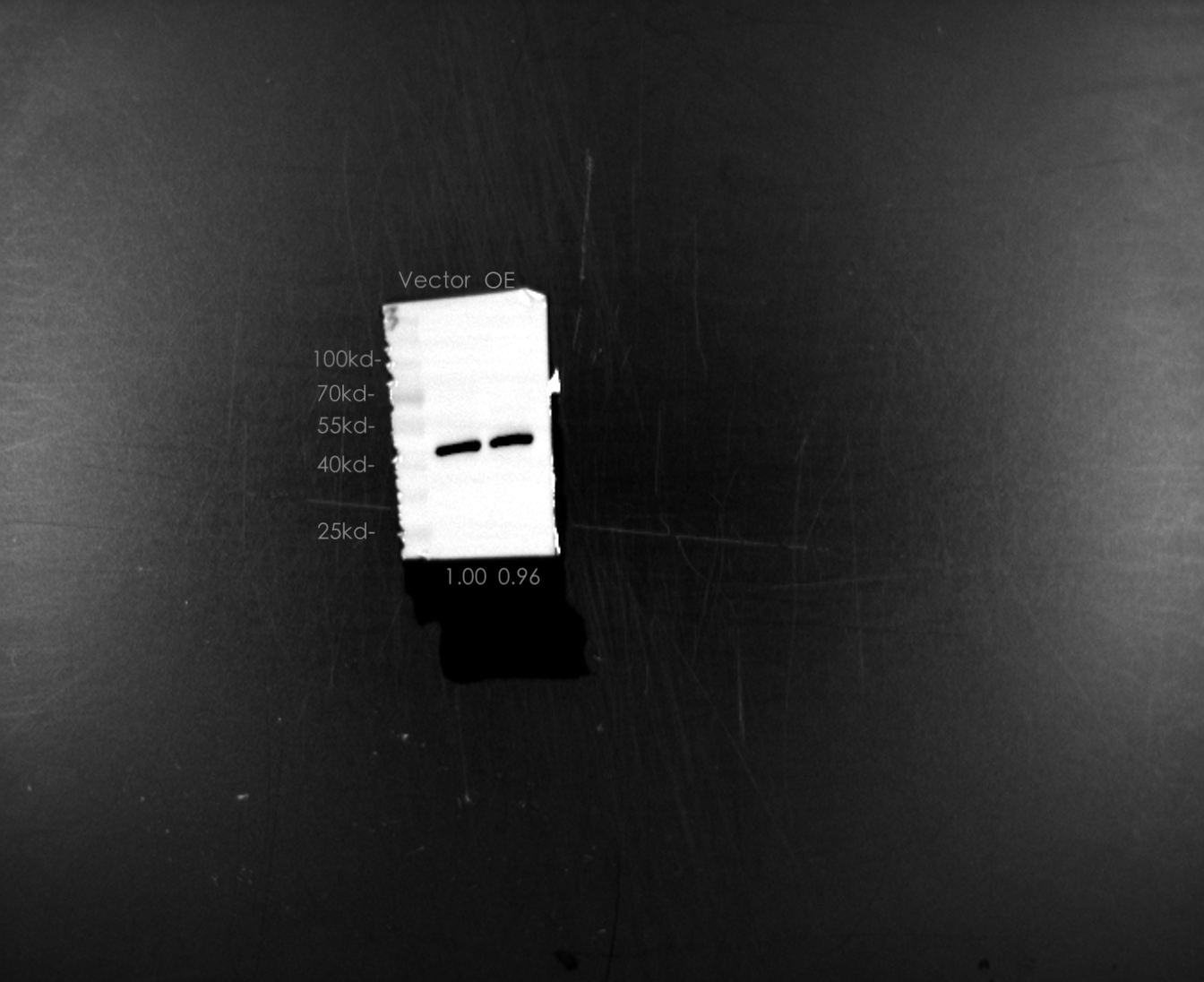

Supplement: Supplementary file 1 [file cancers-16-03028-s001.zip › File S1/For Figure 4/H-231-OE-ACTIN-M.Tif]

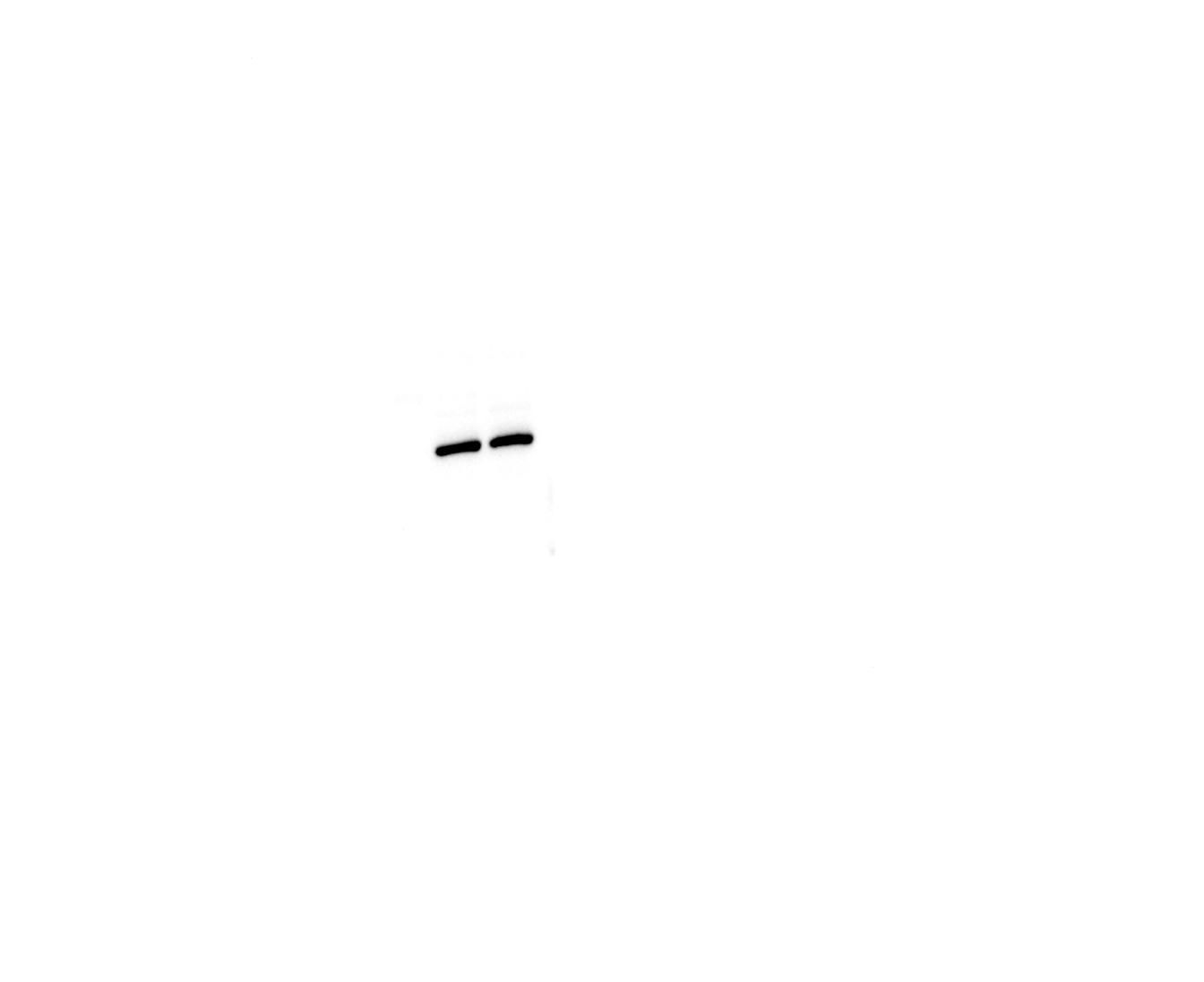

Supplement: Supplementary file 1 [file cancers-16-03028-s001.zip › File S1/For Figure 4/H-231-OE-ACTIN.Tif]

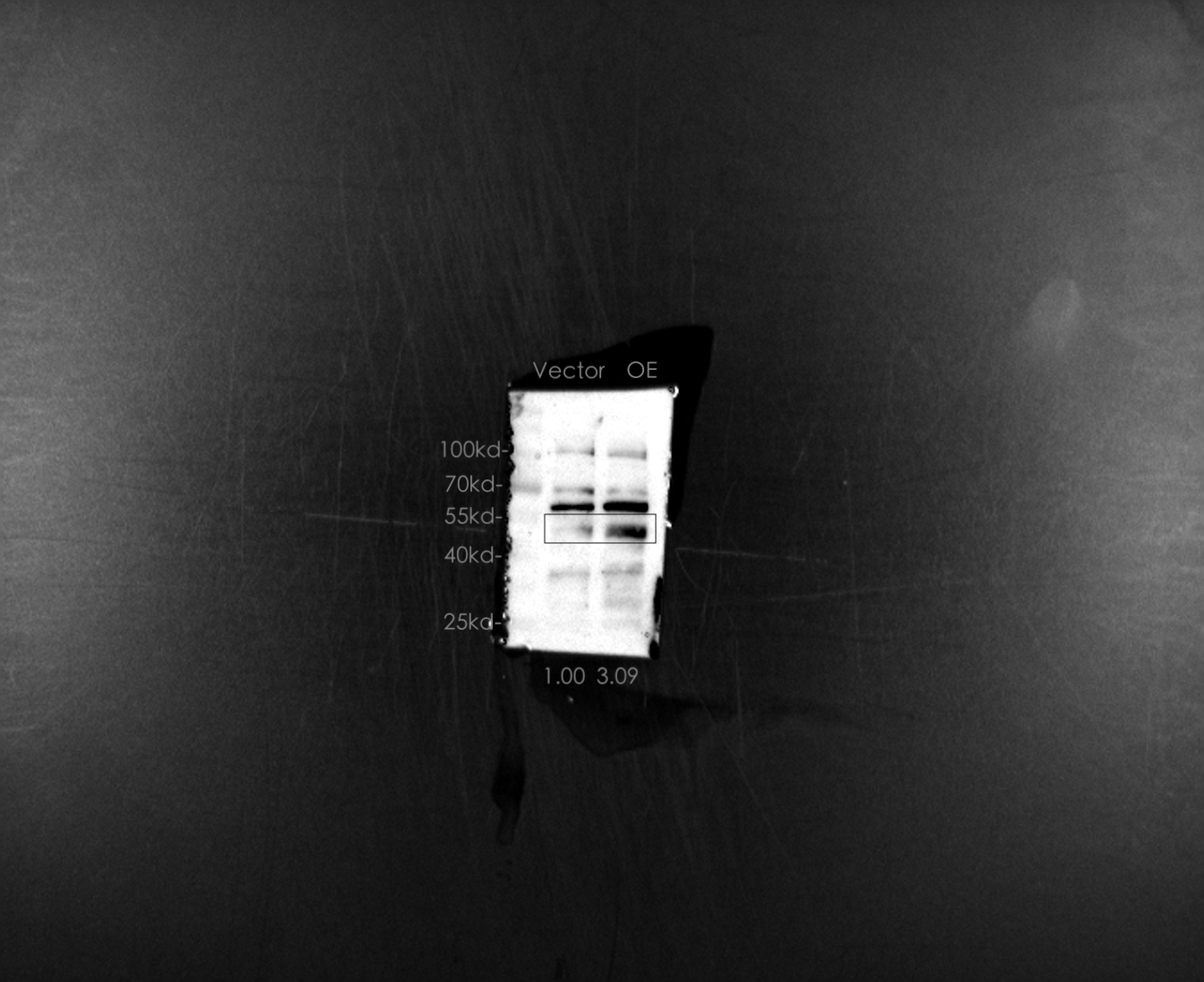

Supplement: Supplementary file 1 [file cancers-16-03028-s001.zip › File S1/For Figure 4/H-231-OE-PPAR-M.Tif]

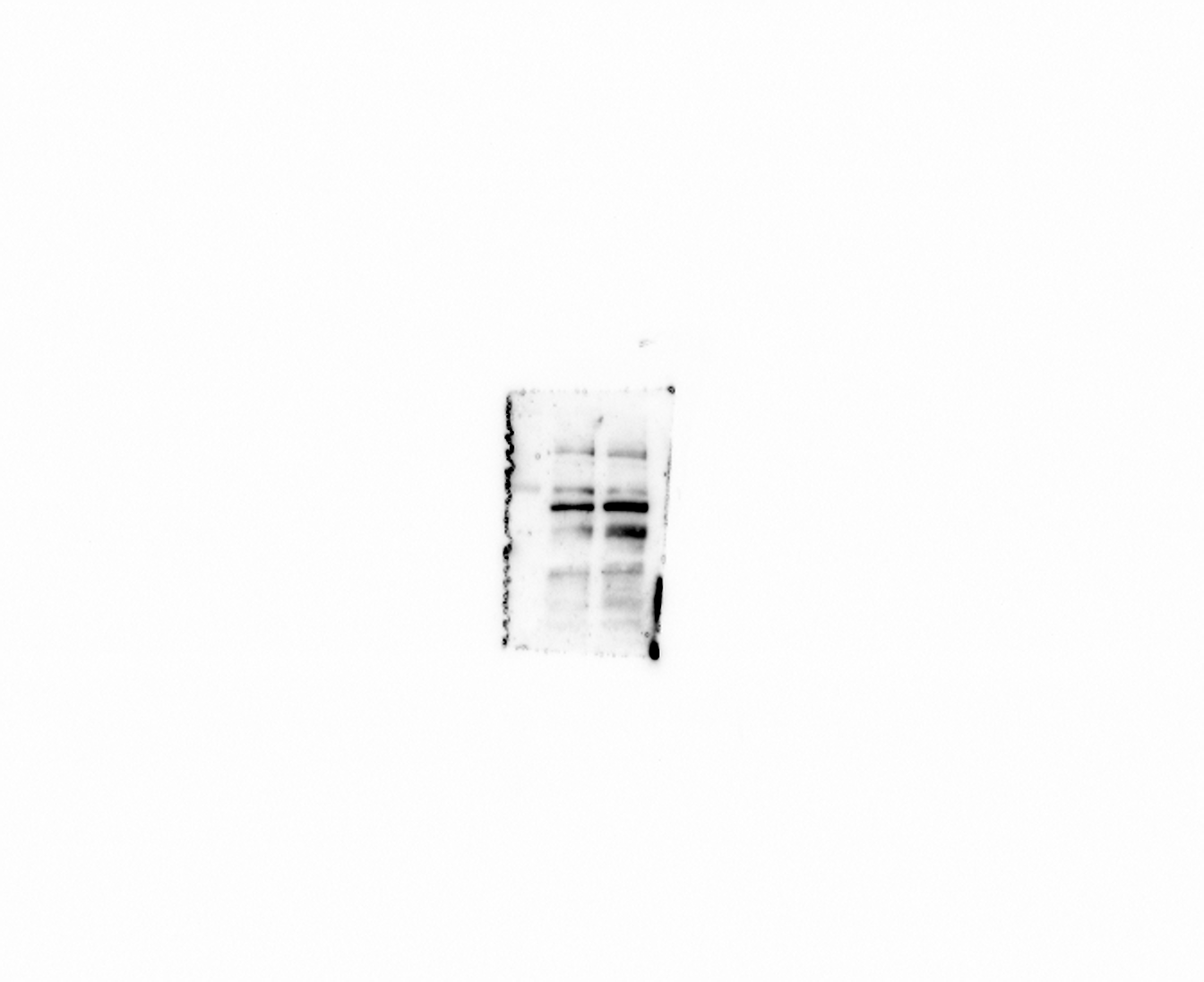

Supplement: Supplementary file 1 [file cancers-16-03028-s001.zip › File S1/For Figure 4/H-231-OE-PPAR.Tif]

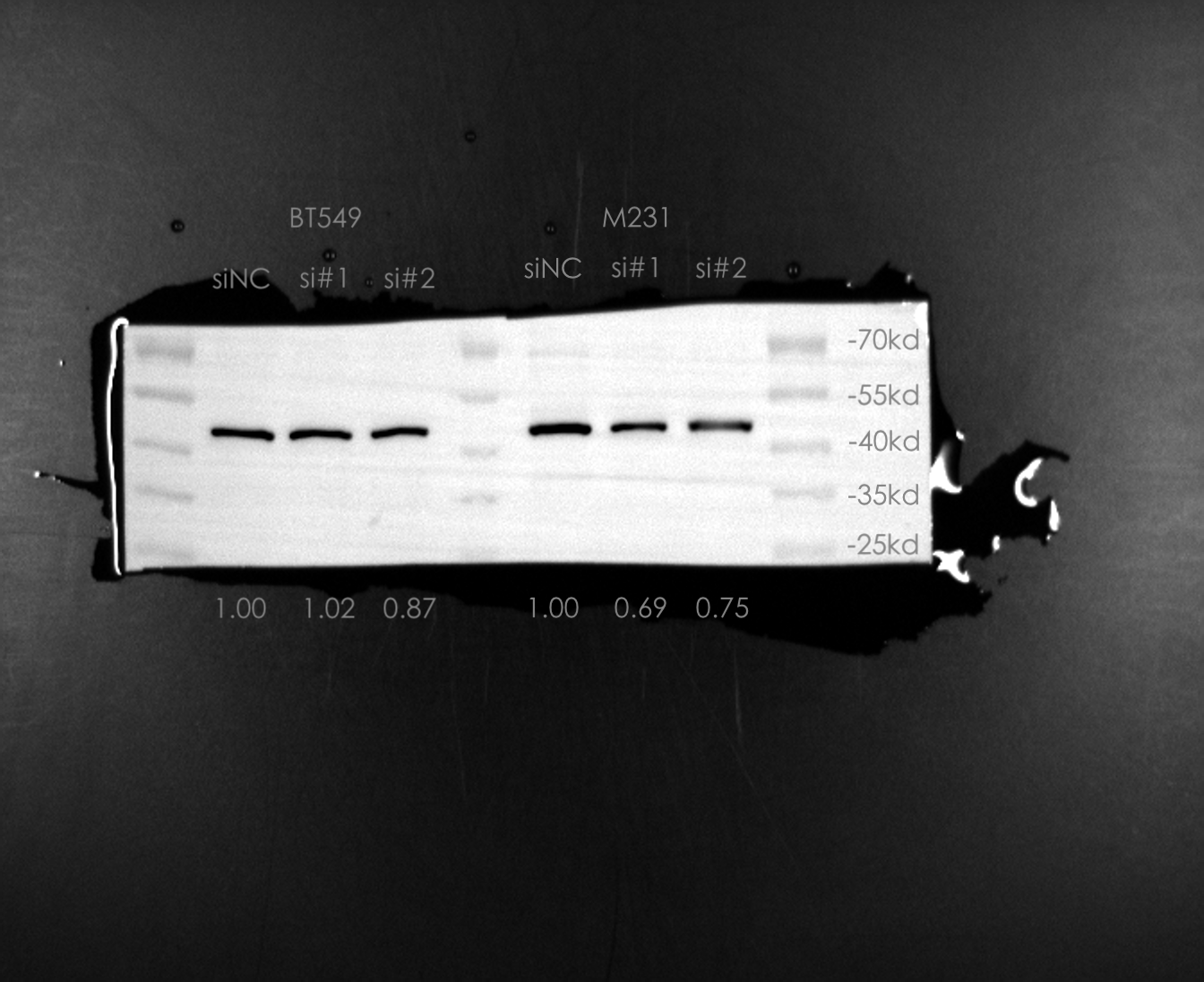

Supplement: Supplementary file 1 [file cancers-16-03028-s001.zip › File S1/For Figure 4/H-549-231-si-ACTIN-M.Tif]

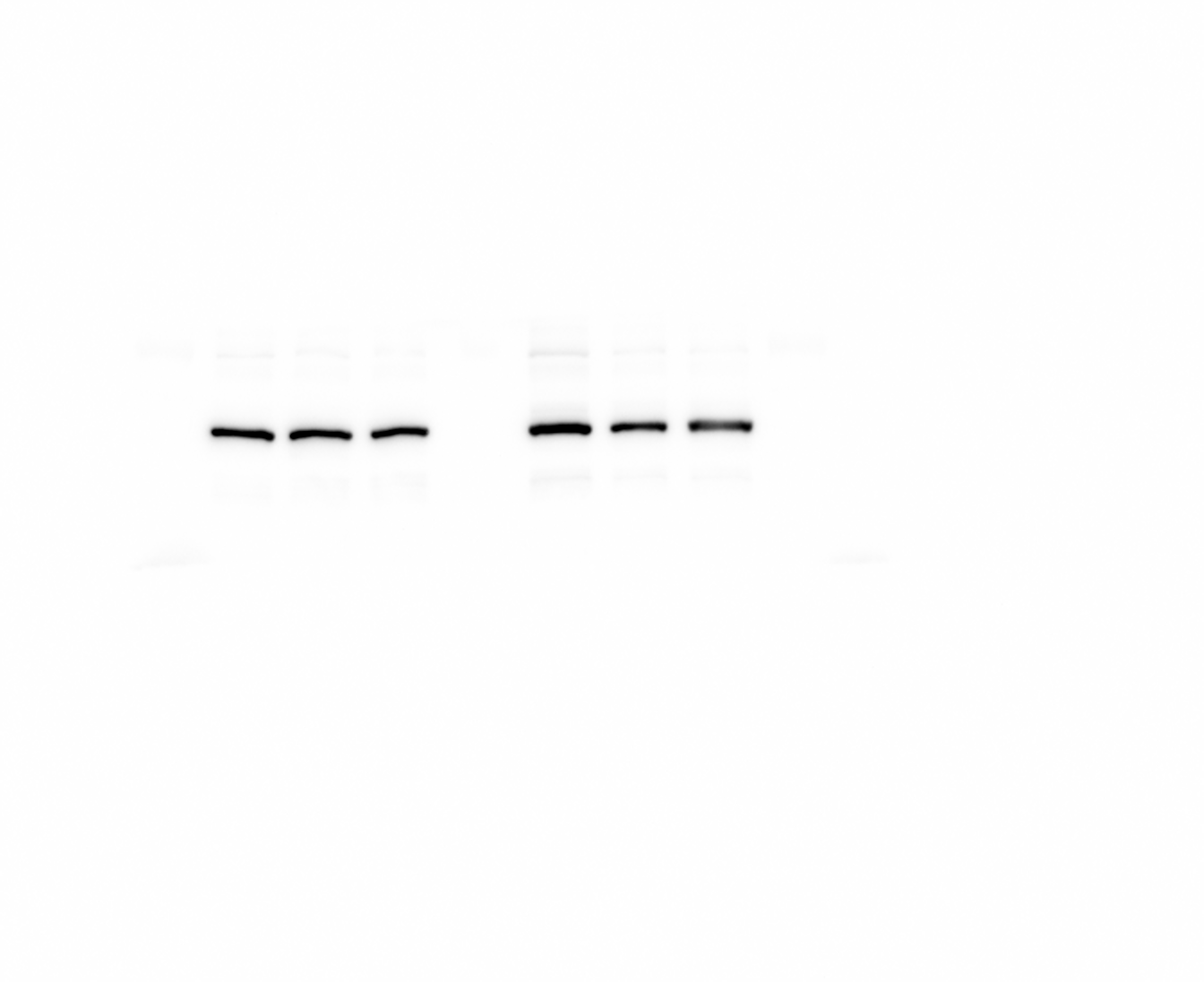

Supplement: Supplementary file 1 [file cancers-16-03028-s001.zip › File S1/For Figure 4/H-549-231-si-ACTIN.Tif]

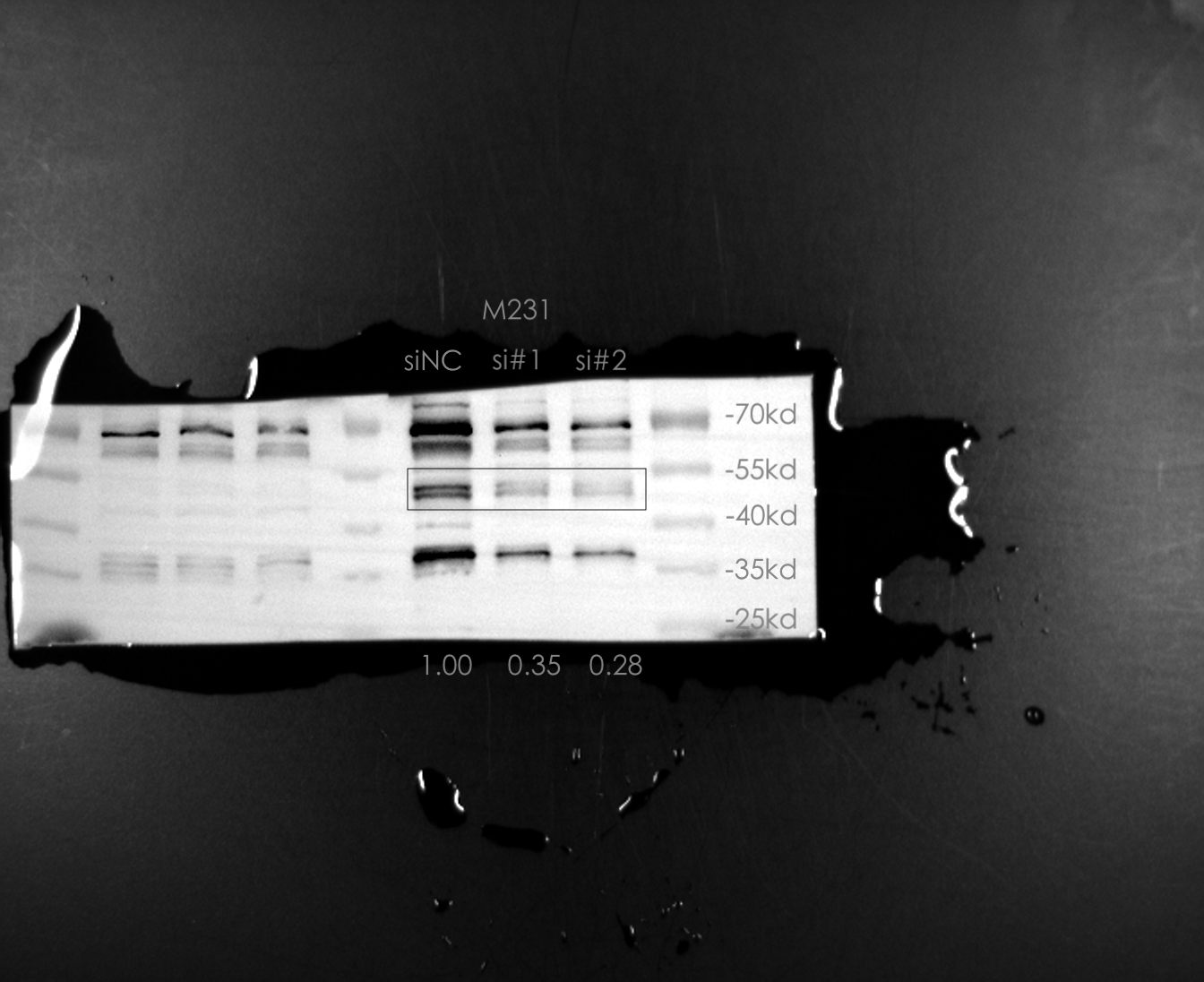

Supplement: Supplementary file 1 [file cancers-16-03028-s001.zip › File S1/For Figure 4/H-549-231-si-PPAR-M.Tif]

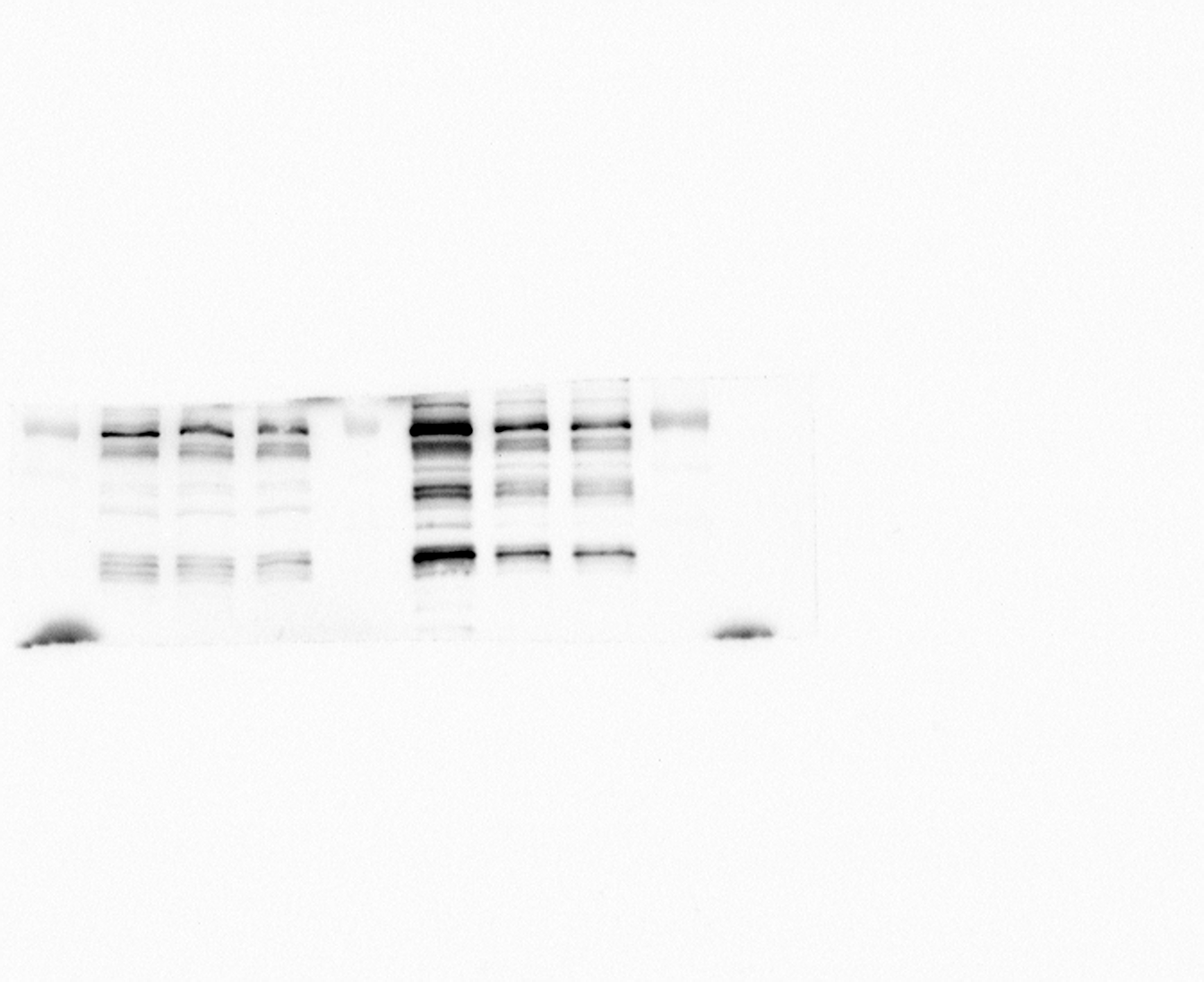

Supplement: Supplementary file 1 [file cancers-16-03028-s001.zip › File S1/For Figure 4/H-549-231-si-PPAR.Tif]

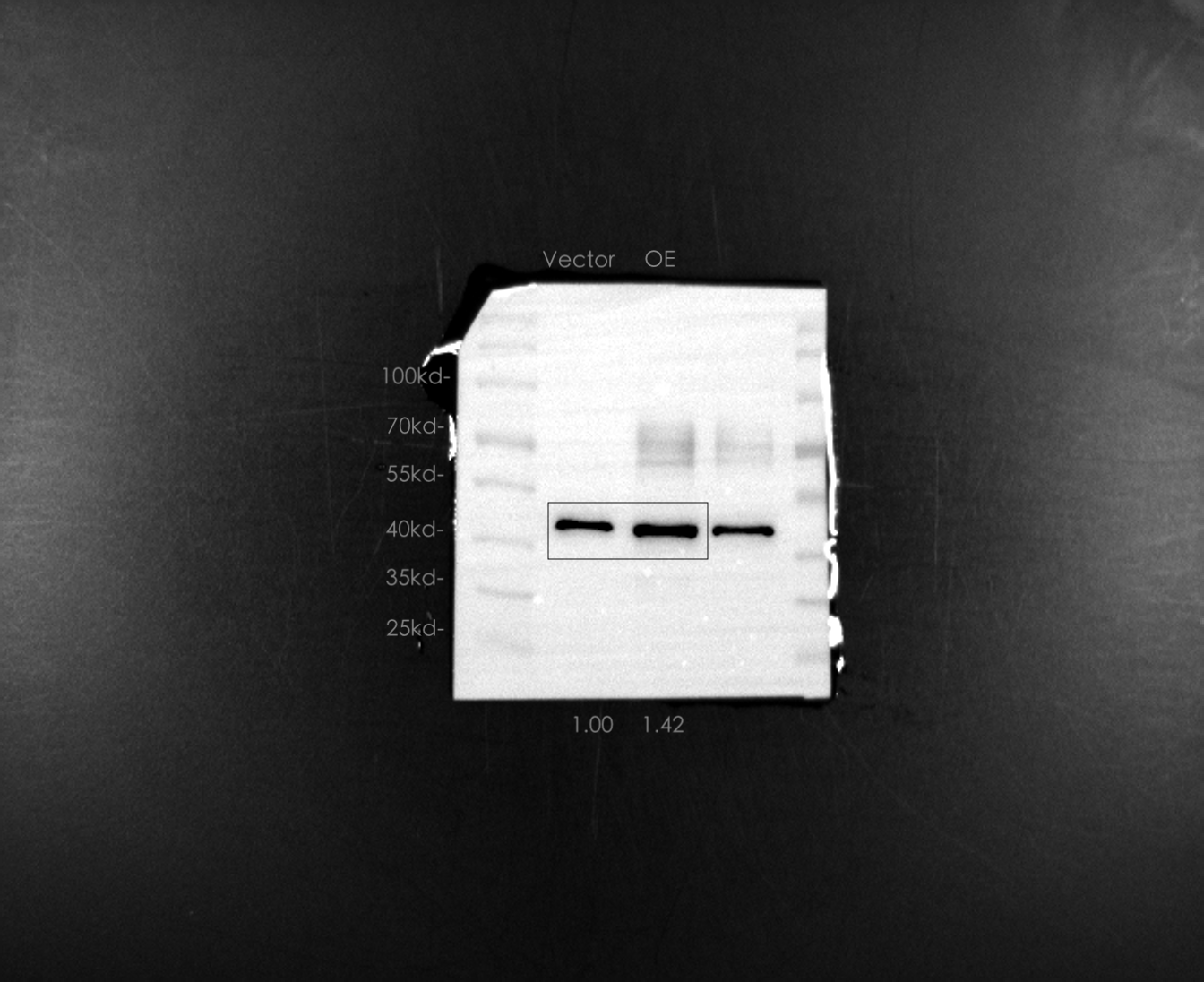

Supplement: Supplementary file 1 [file cancers-16-03028-s001.zip › File S1/For Figure 4/H-549-OE-ACTIN-M.Tif]

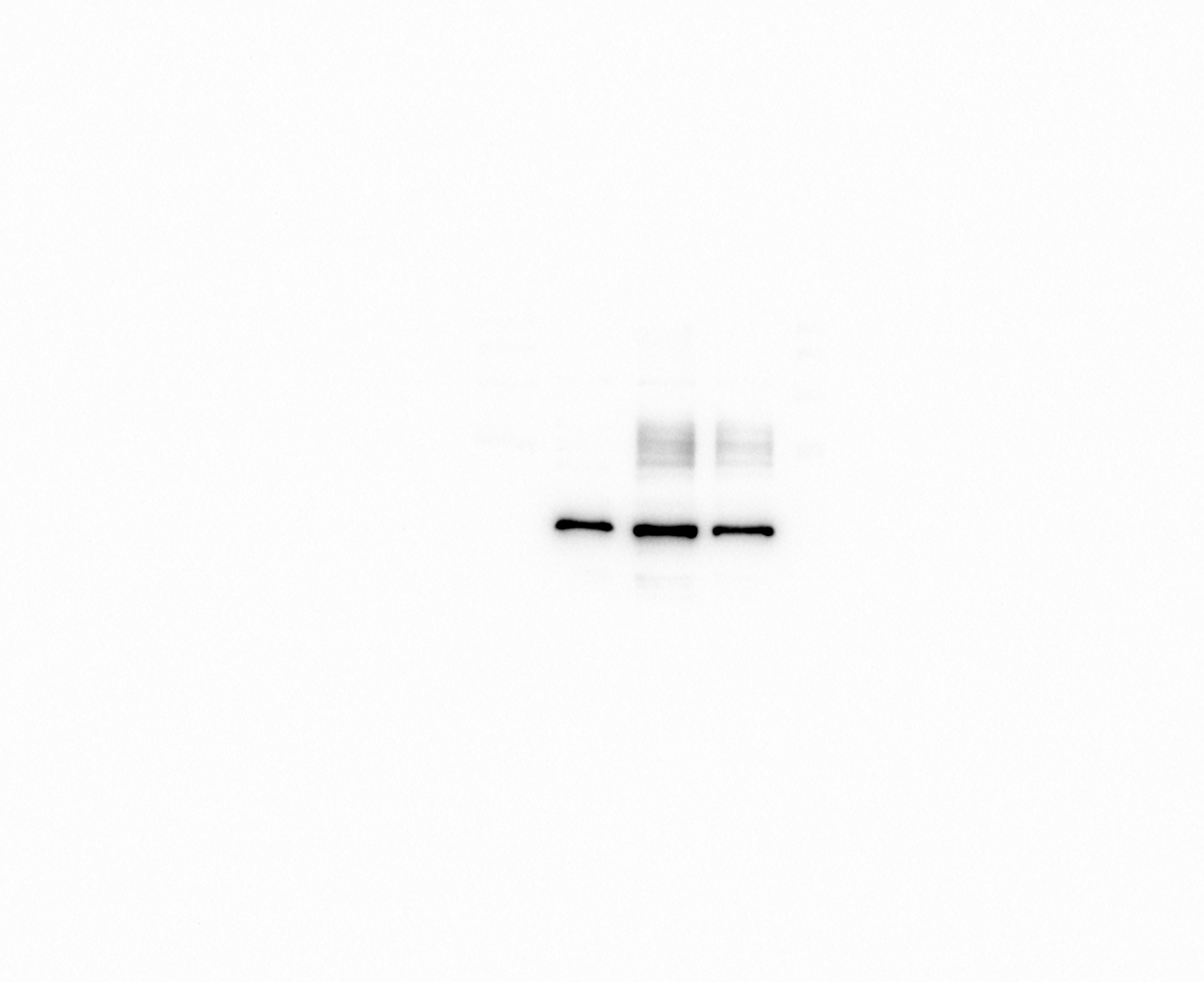

Supplement: Supplementary file 1 [file cancers-16-03028-s001.zip › File S1/For Figure 4/H-549-OE-ACTIN.Tif]

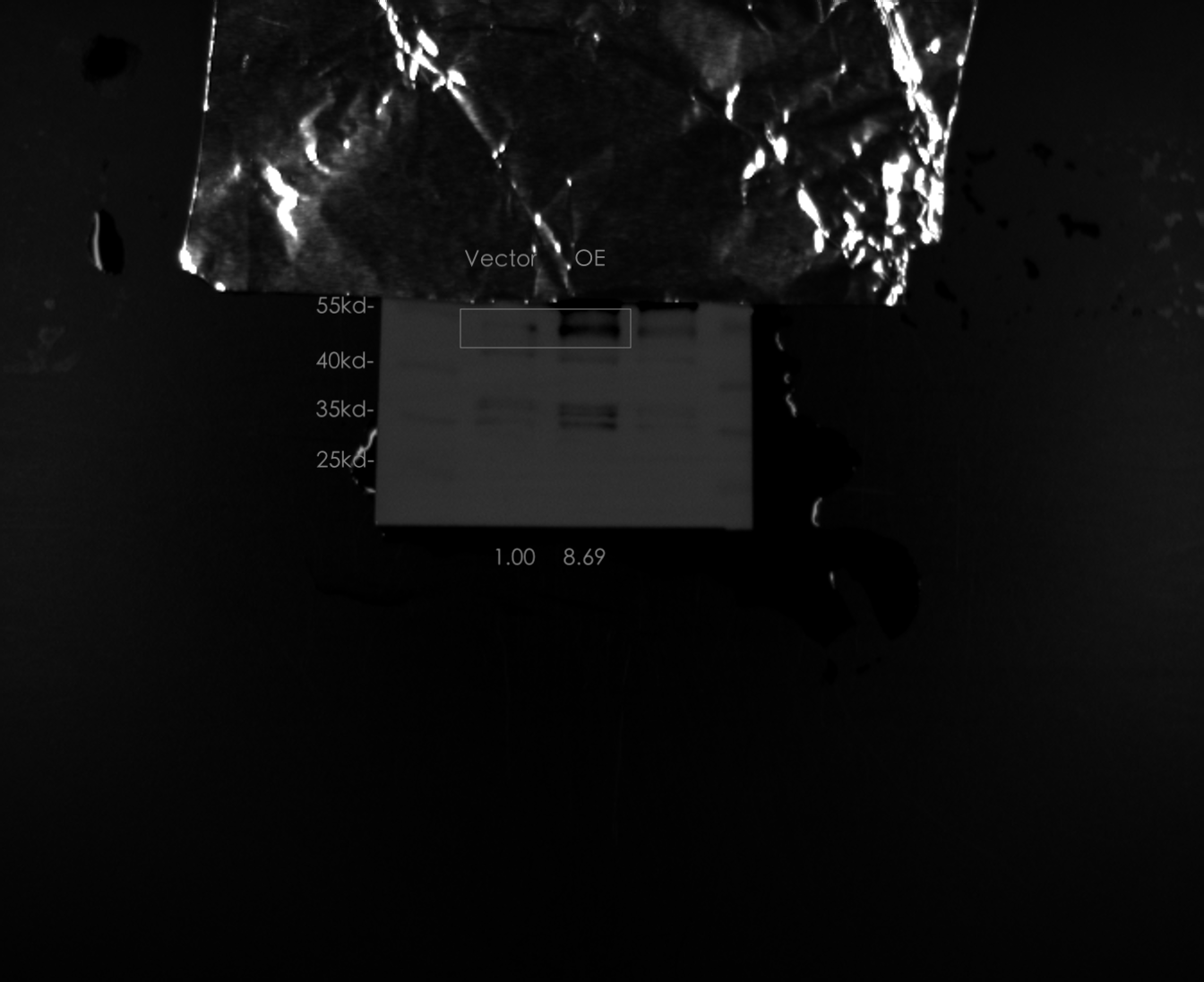

Supplement: Supplementary file 1 [file cancers-16-03028-s001.zip › File S1/For Figure 4/H-549-OE-PPAR-M.Tif]

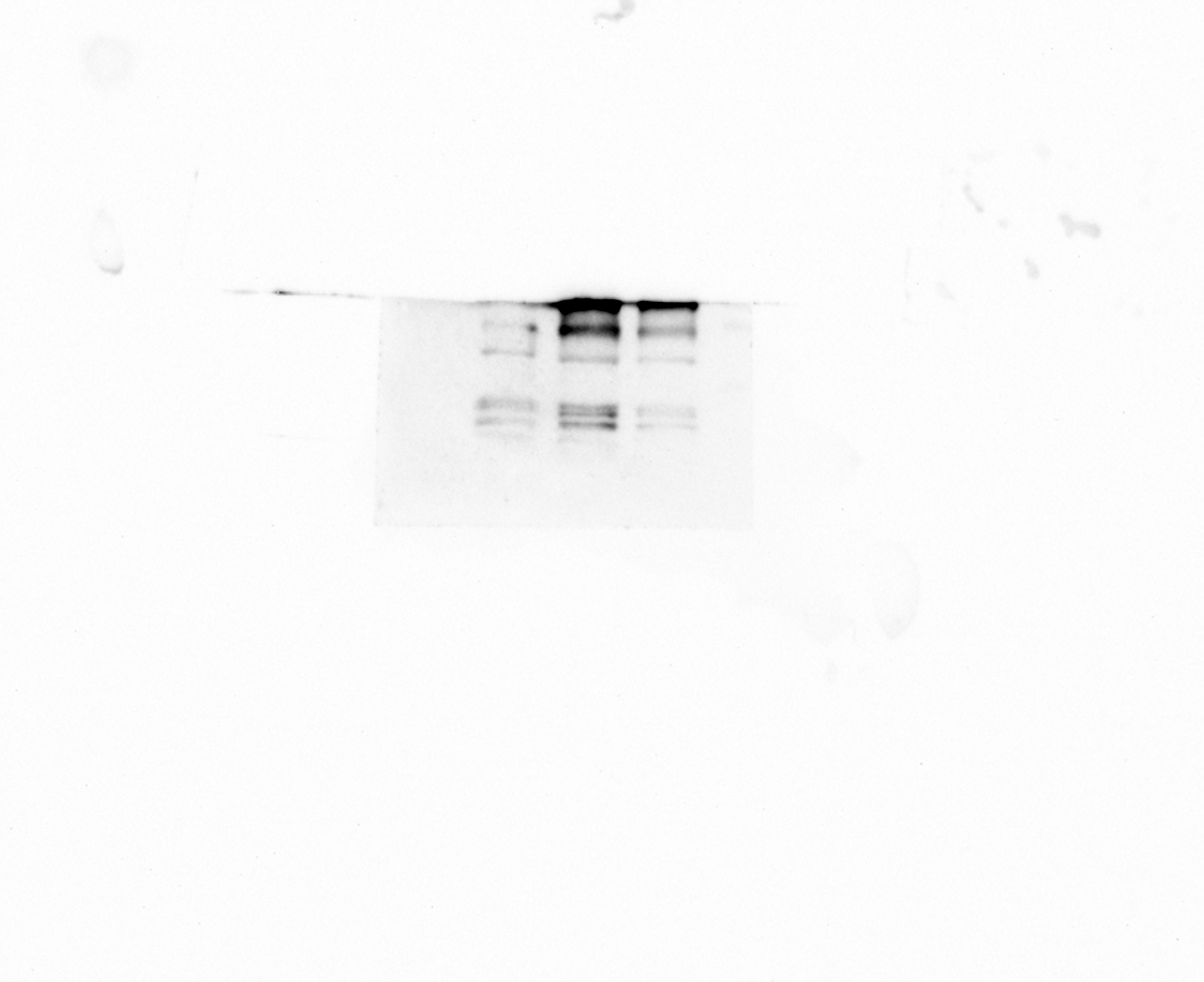

Supplement: Supplementary file 1 [file cancers-16-03028-s001.zip › File S1/For Figure 4/H-549-OE-PPAR.Tif]

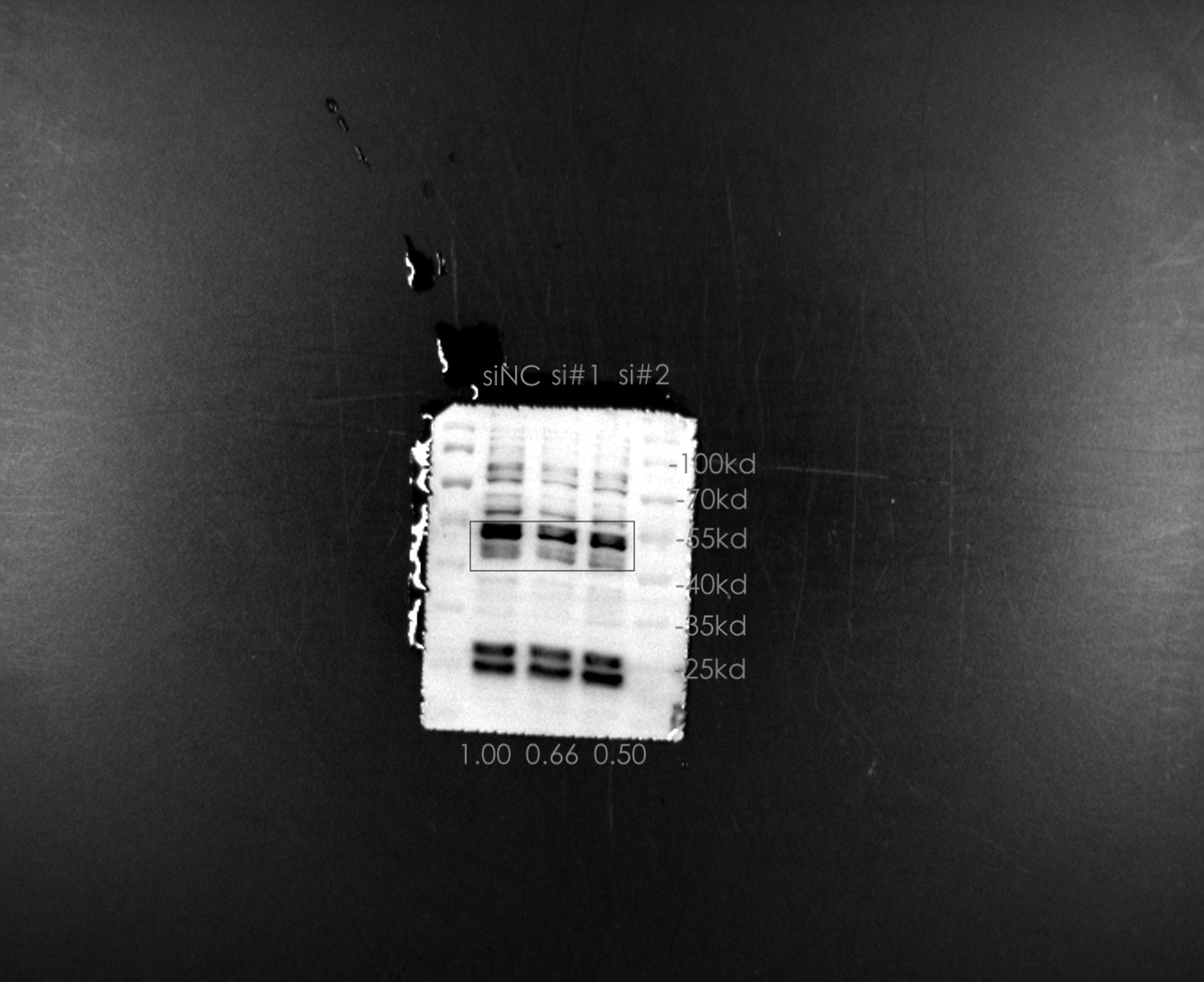

Supplement: Supplementary file 1 [file cancers-16-03028-s001.zip › File S1/For Figure 4/H-549-si-PPAR-M.Tif]

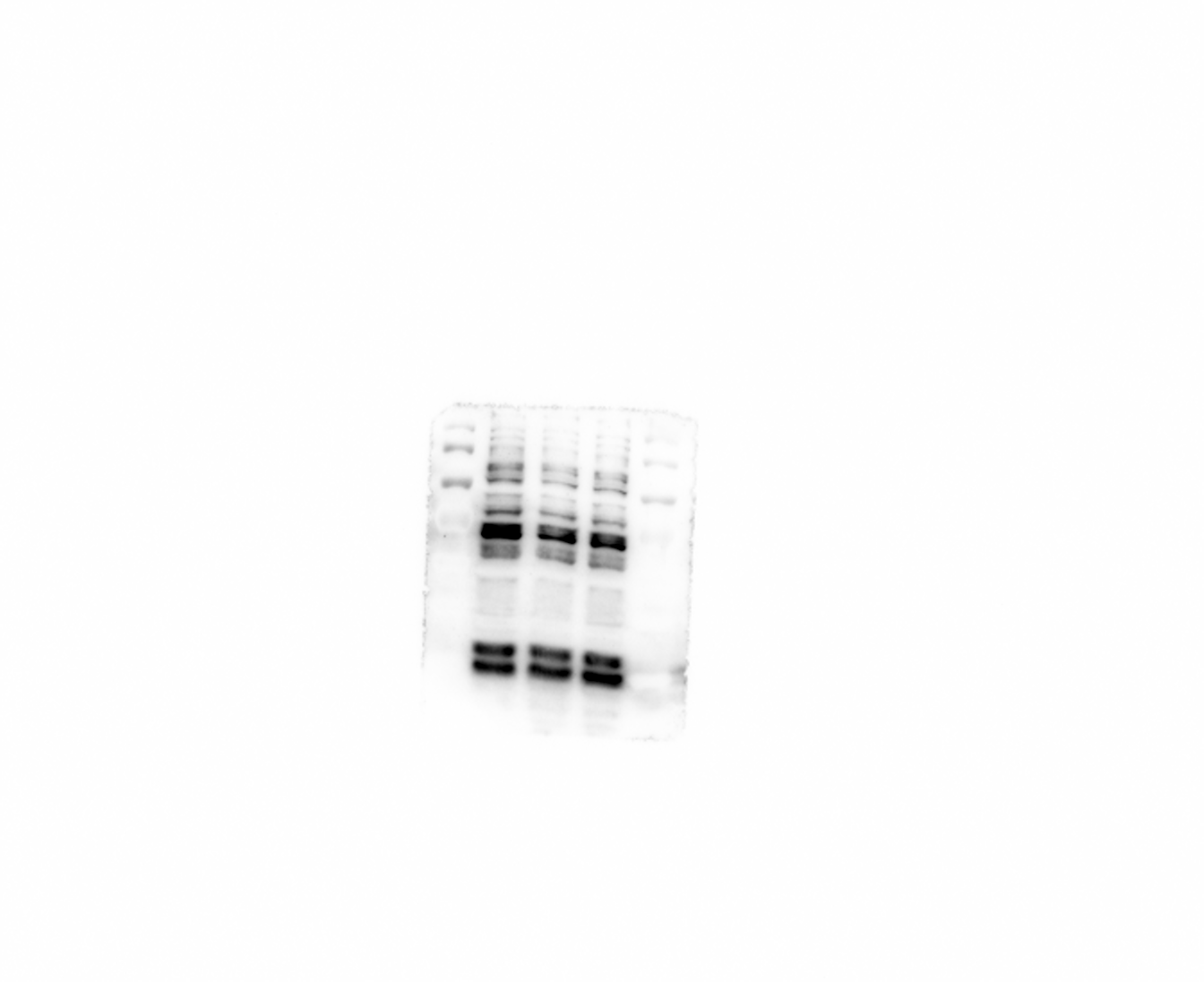

Supplement: Supplementary file 1 [file cancers-16-03028-s001.zip › File S1/For Figure 4/H-549-si-PPAR.Tif]

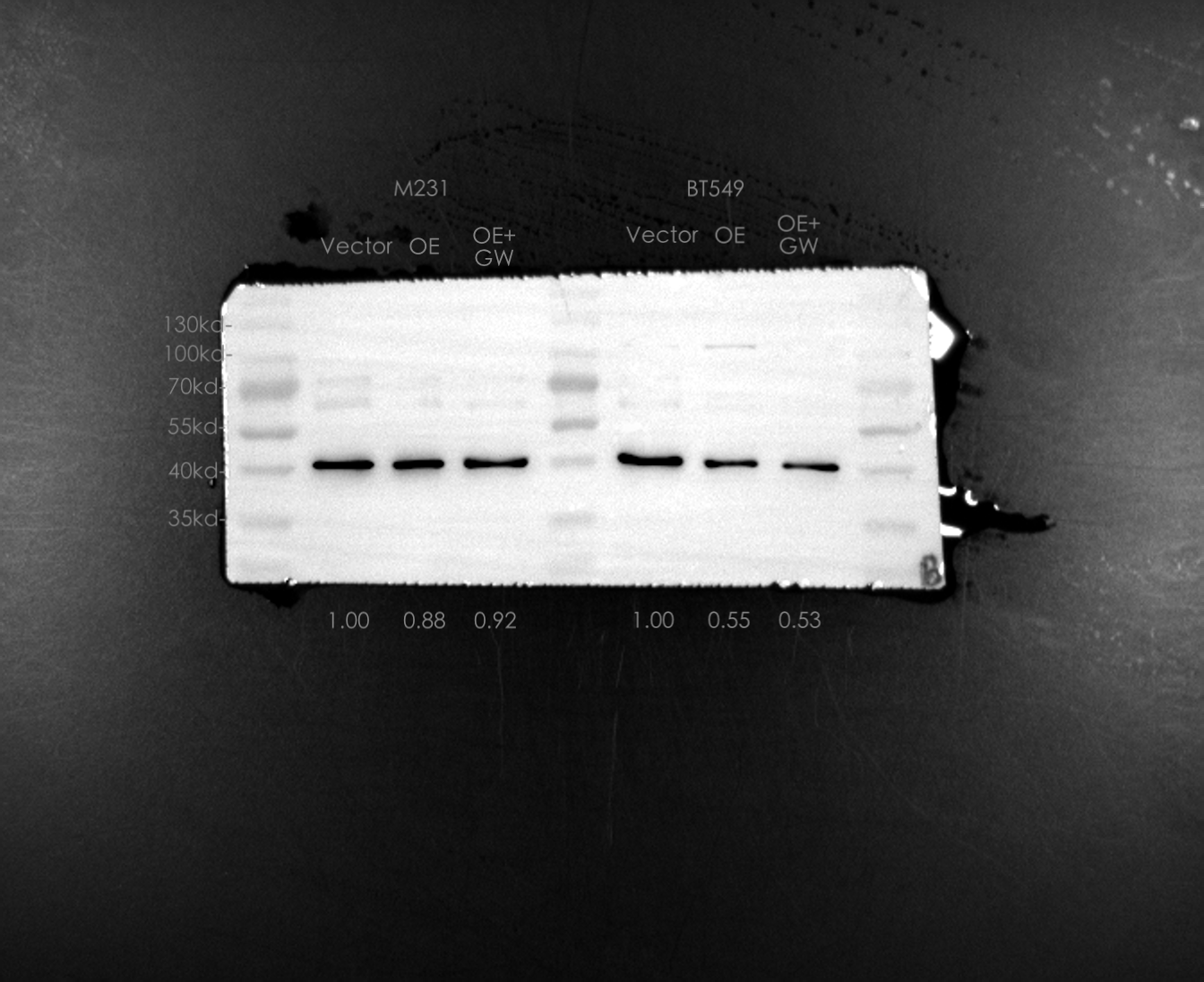

Supplement: Supplementary file 1 [file cancers-16-03028-s001.zip › File S1/For Figure 4/J-231-549-OE-actin-M.Tif]

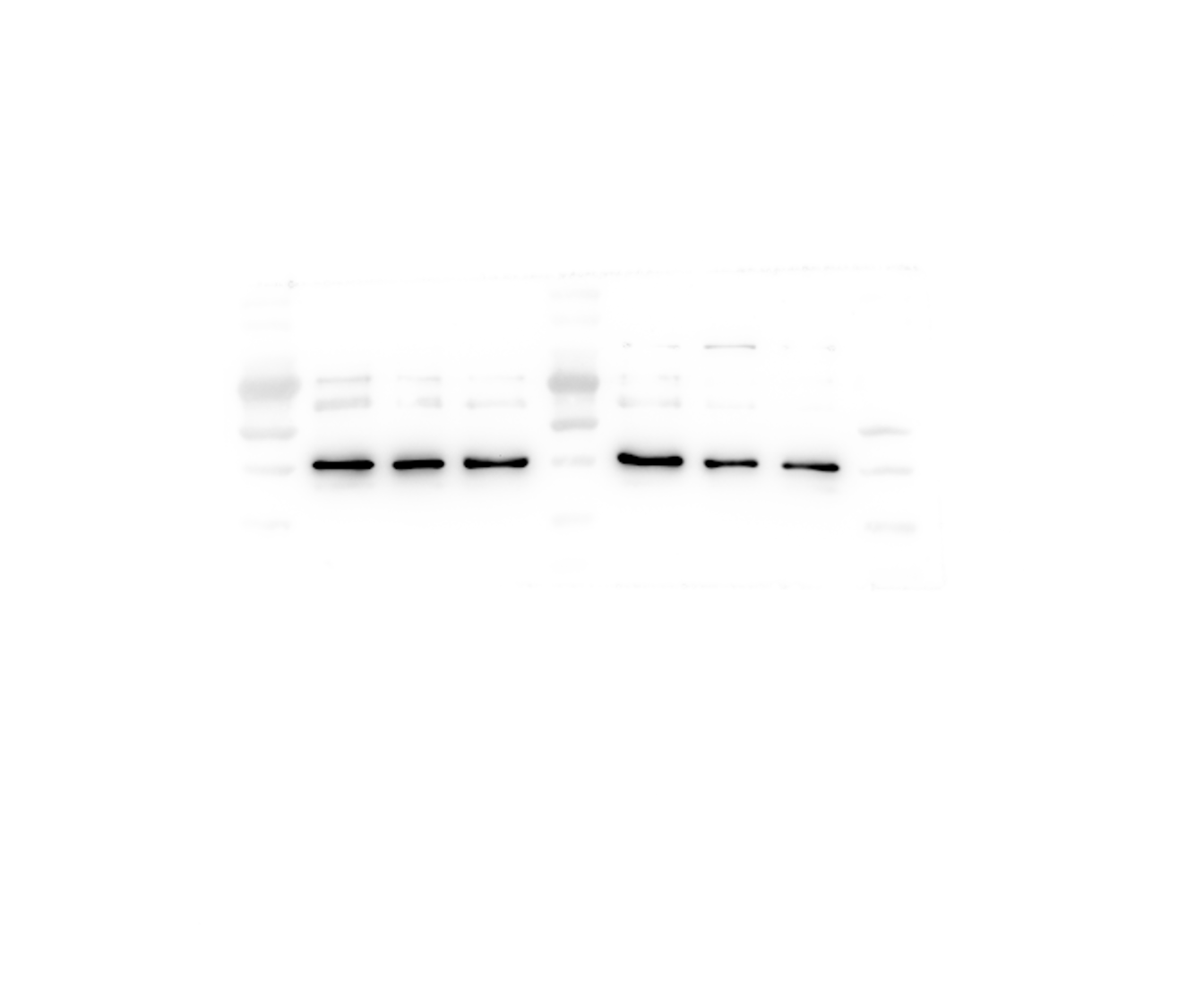

Supplement: Supplementary file 1 [file cancers-16-03028-s001.zip › File S1/For Figure 4/J-231-549-OE-actin.Tif]

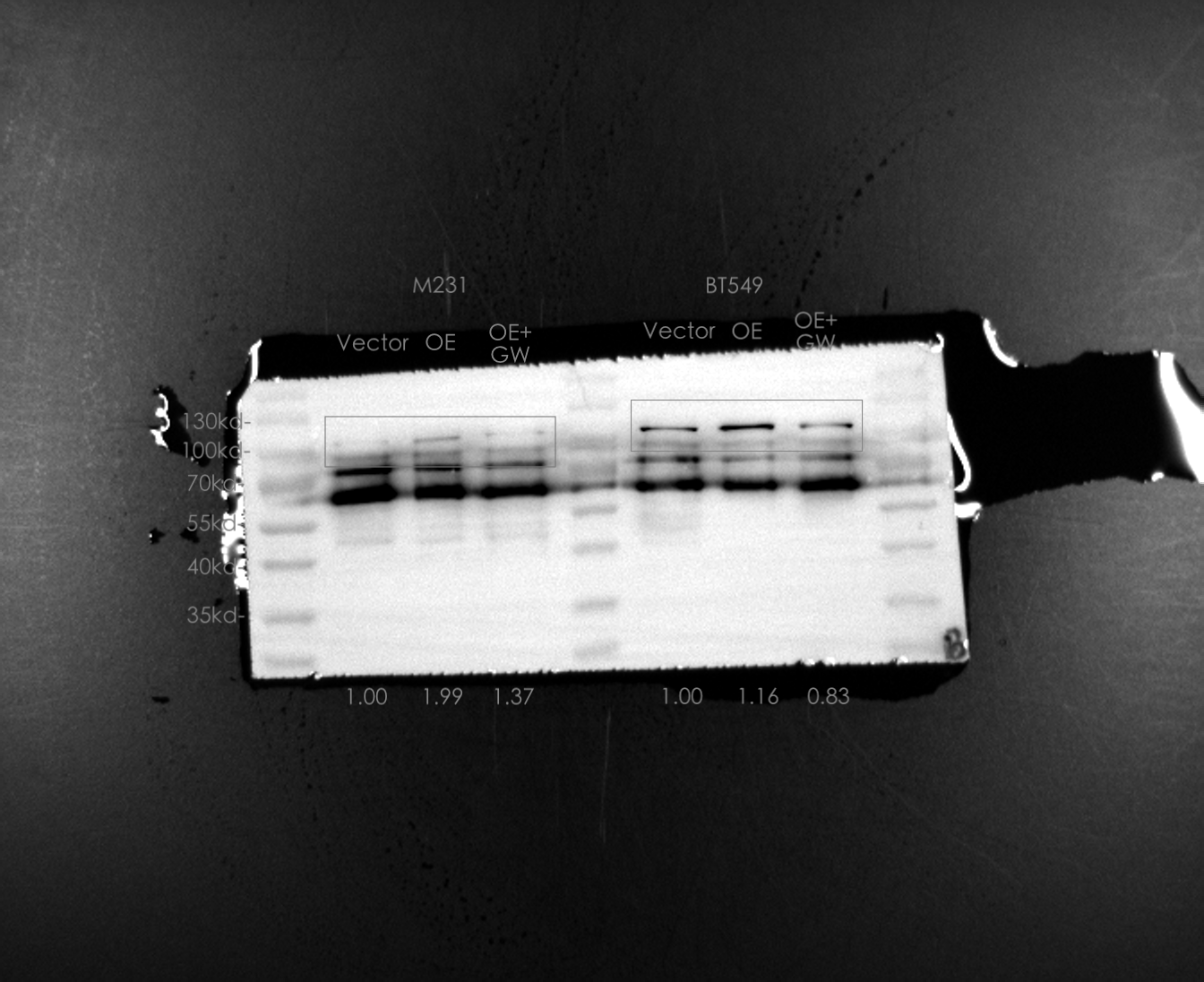

Supplement: Supplementary file 1 [file cancers-16-03028-s001.zip › File S1/For Figure 4/J-231-549-OE-CPT1B-M.Tif]

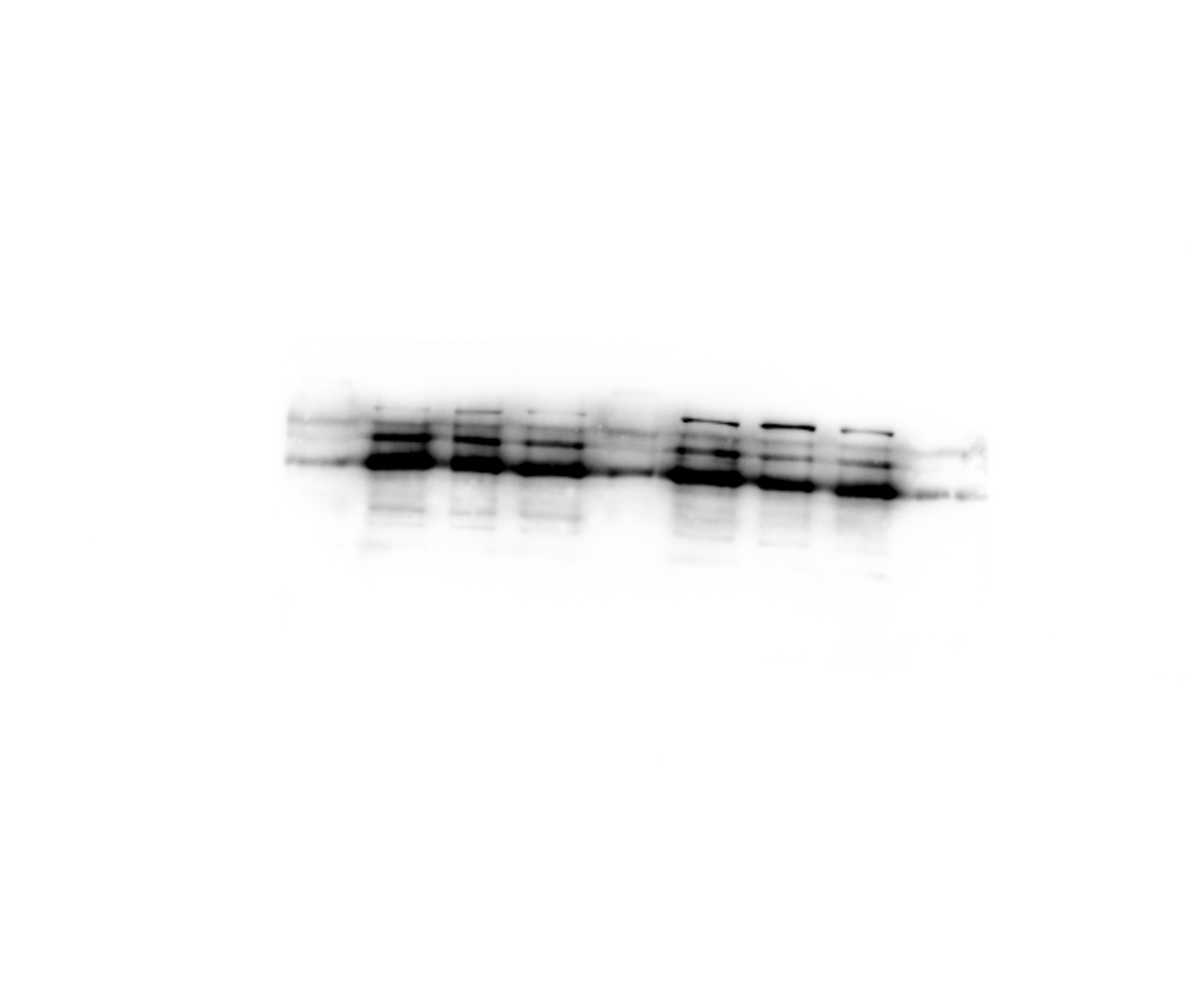

Supplement: Supplementary file 1 [file cancers-16-03028-s001.zip › File S1/For Figure 4/J-231-549-OE-CPT1B.Tif]

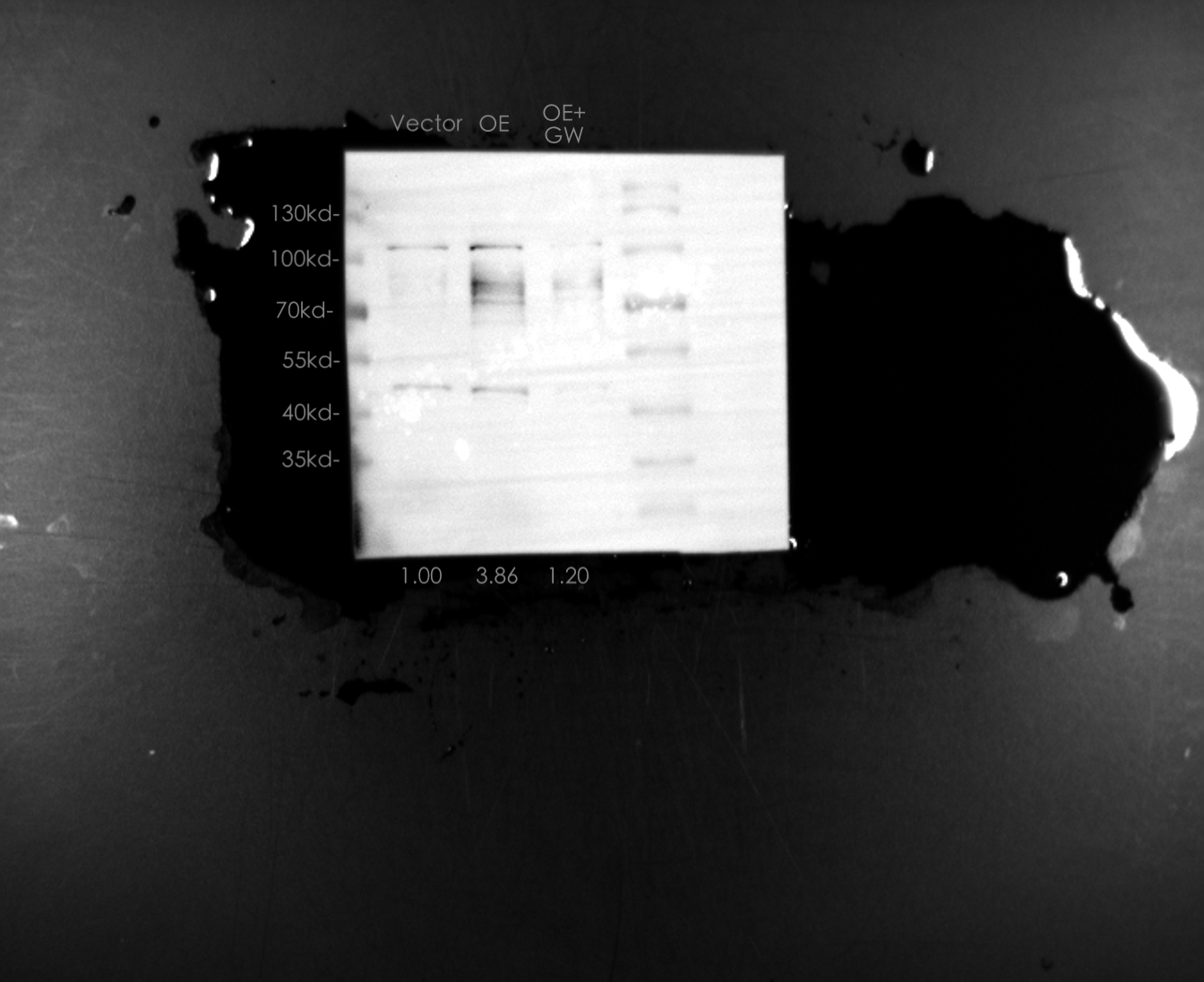

Supplement: Supplementary file 1 [file cancers-16-03028-s001.zip › File S1/For Figure 4/J-231-OE-ASCT2-M.Tif]

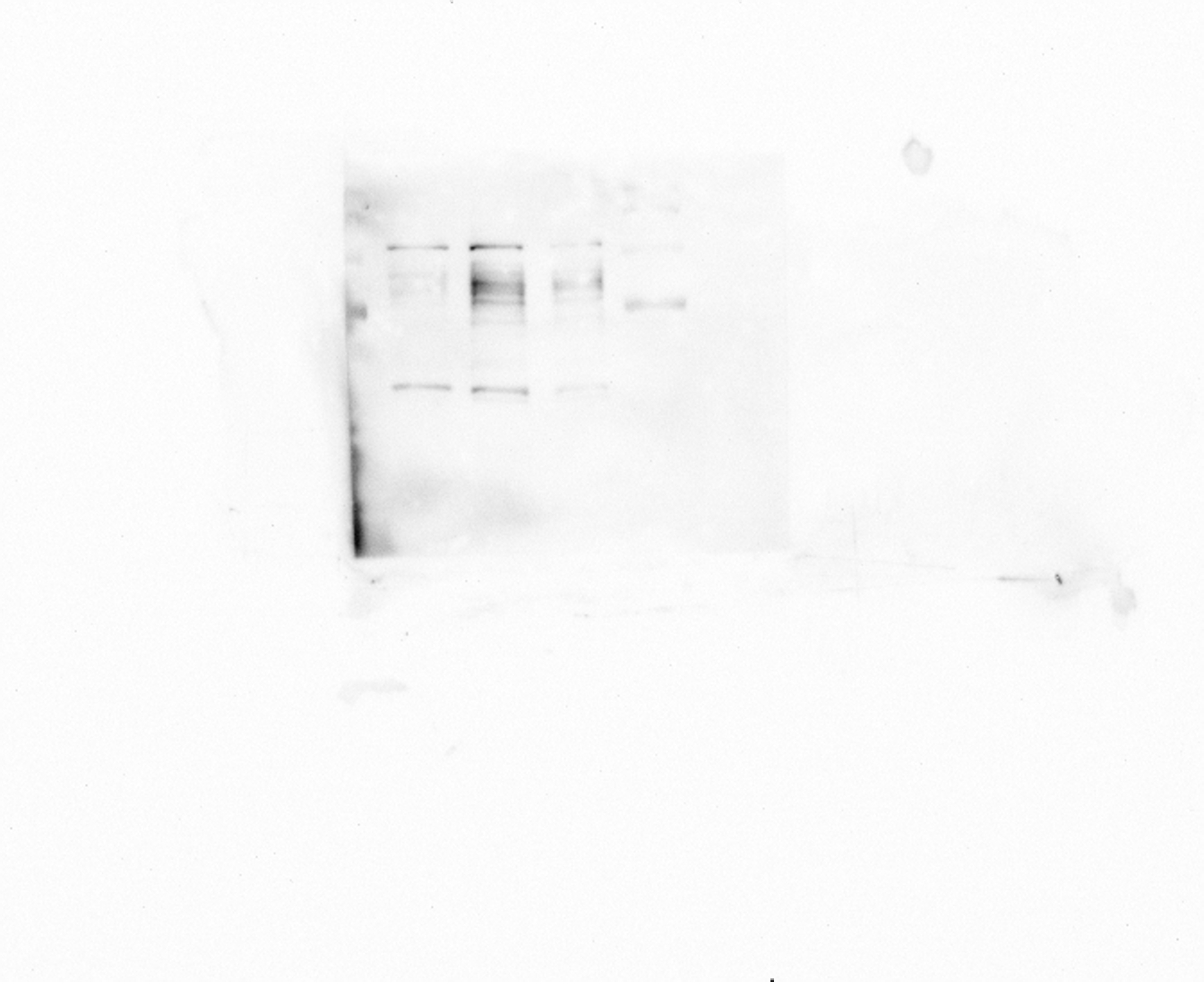

Supplement: Supplementary file 1 [file cancers-16-03028-s001.zip › File S1/For Figure 4/J-231-OE-ASCT2.Tif]

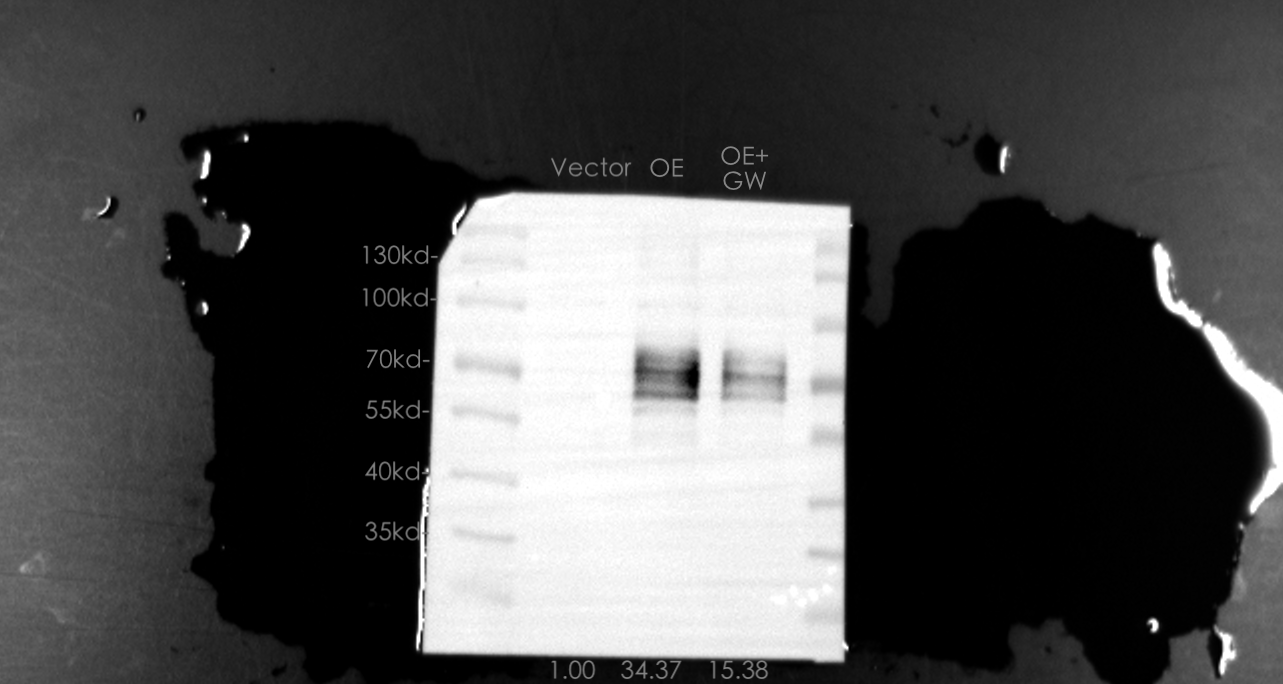

Supplement: Supplementary file 1 [file cancers-16-03028-s001.zip › File S1/For Figure 4/J-549-OE-ASCT2-M.Tif]

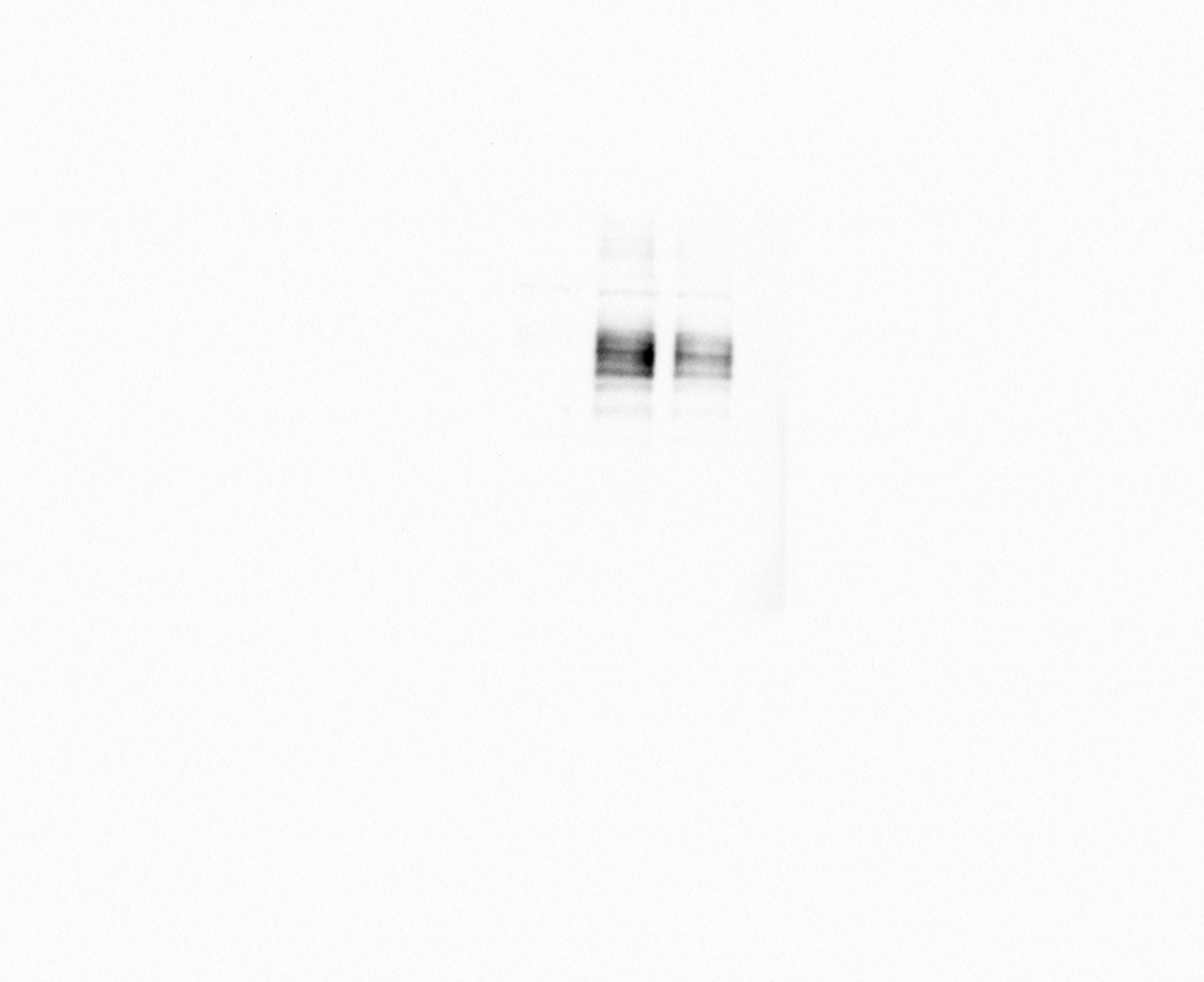

Supplement: Supplementary file 1 [file cancers-16-03028-s001.zip › File S1/For Figure 4/J-549-OE-ASCT2.Tif]

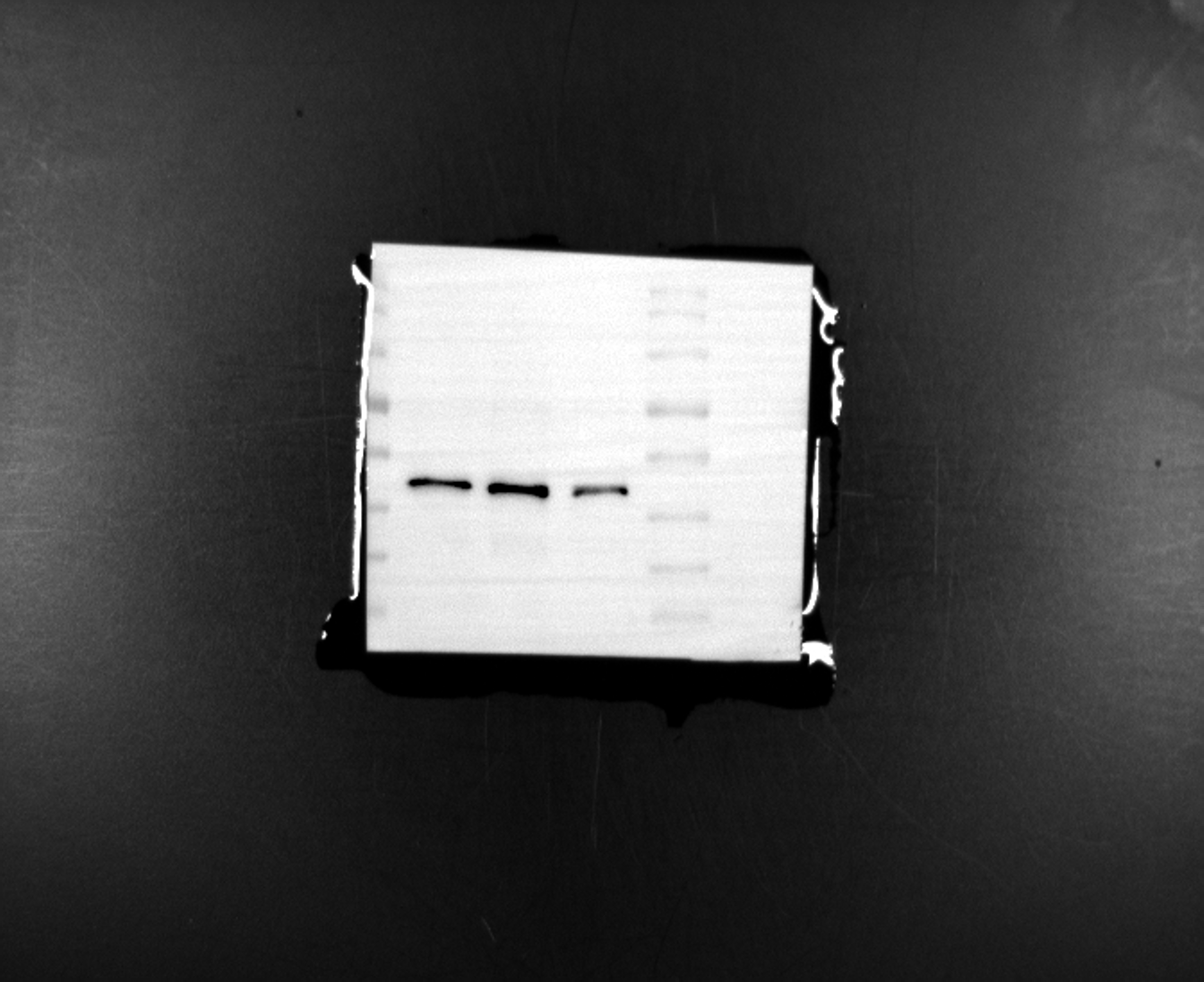

Supplement: Supplementary file 1 [file cancers-16-03028-s001.zip › File S1/For Figure 4/s/231-OE-ACTIN-M.Tif]

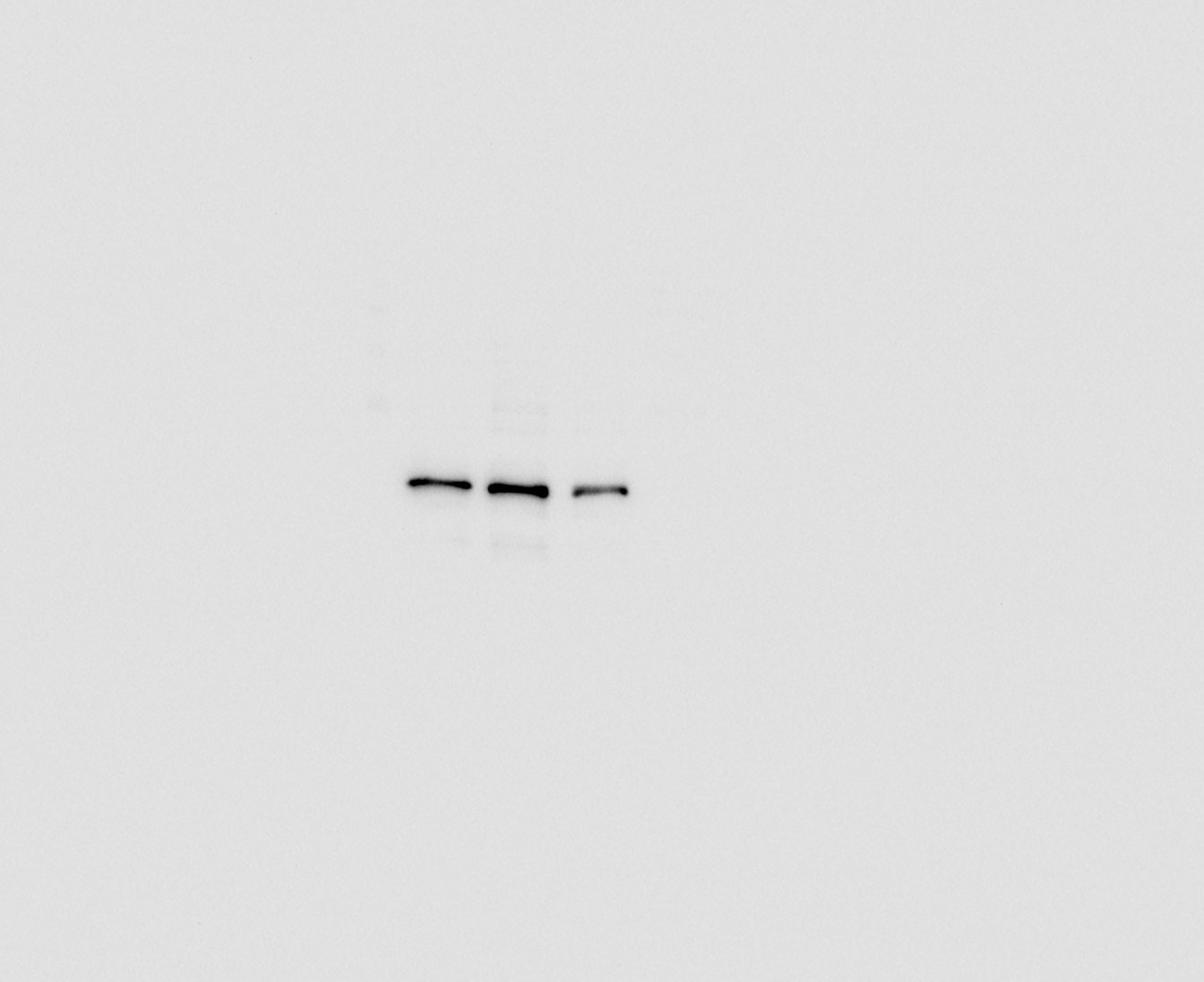

Supplement: Supplementary file 1 [file cancers-16-03028-s001.zip › File S1/For Figure 4/s/231-OE-ACTIN.Tif]

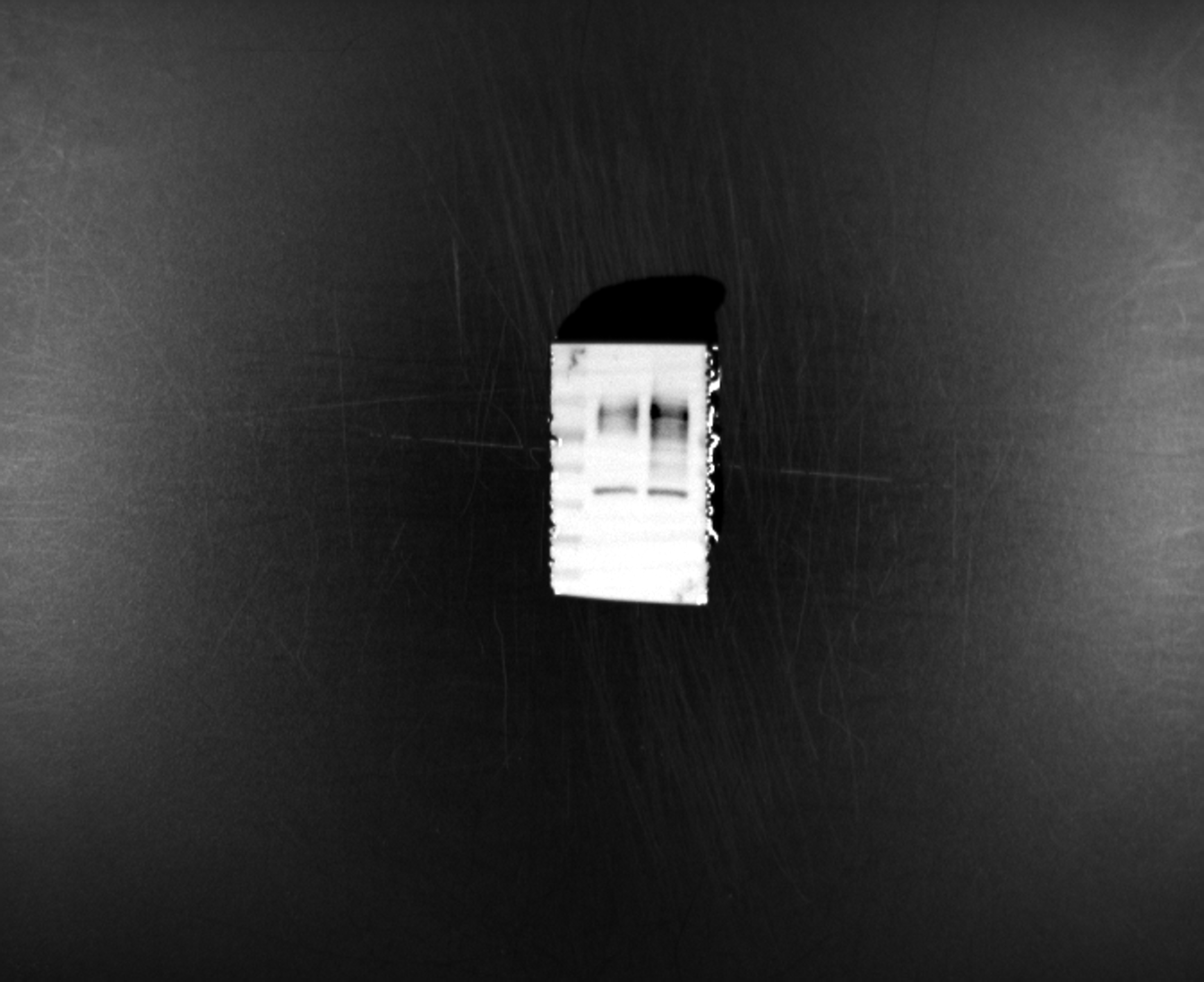

Supplement: Supplementary file 1 [file cancers-16-03028-s001.zip › File S1/For Figure 4/s/231-OE-ASCT2-M.Tif]

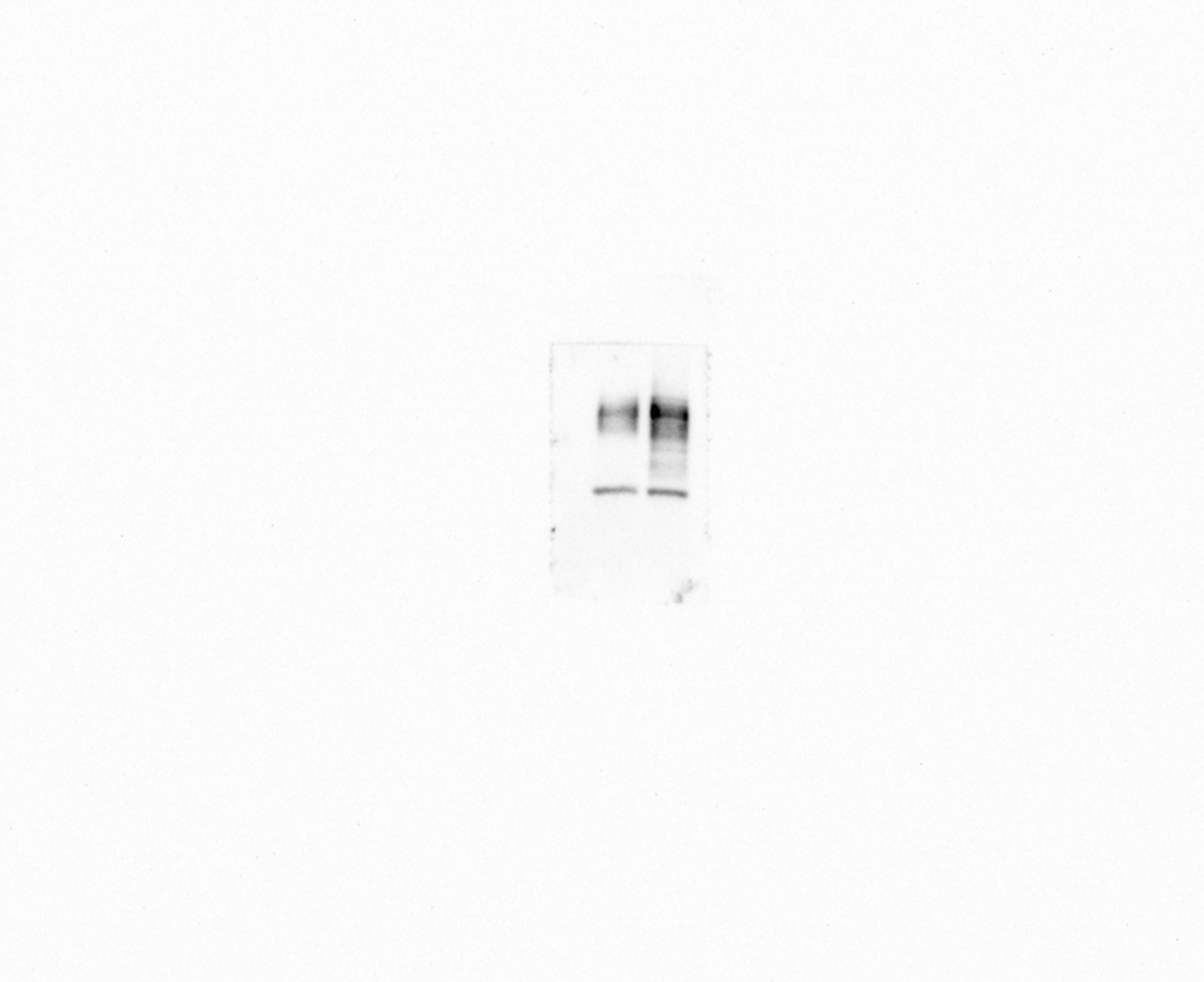

Supplement: Supplementary file 1 [file cancers-16-03028-s001.zip › File S1/For Figure 4/s/231-OE-ASCT2.Tif]

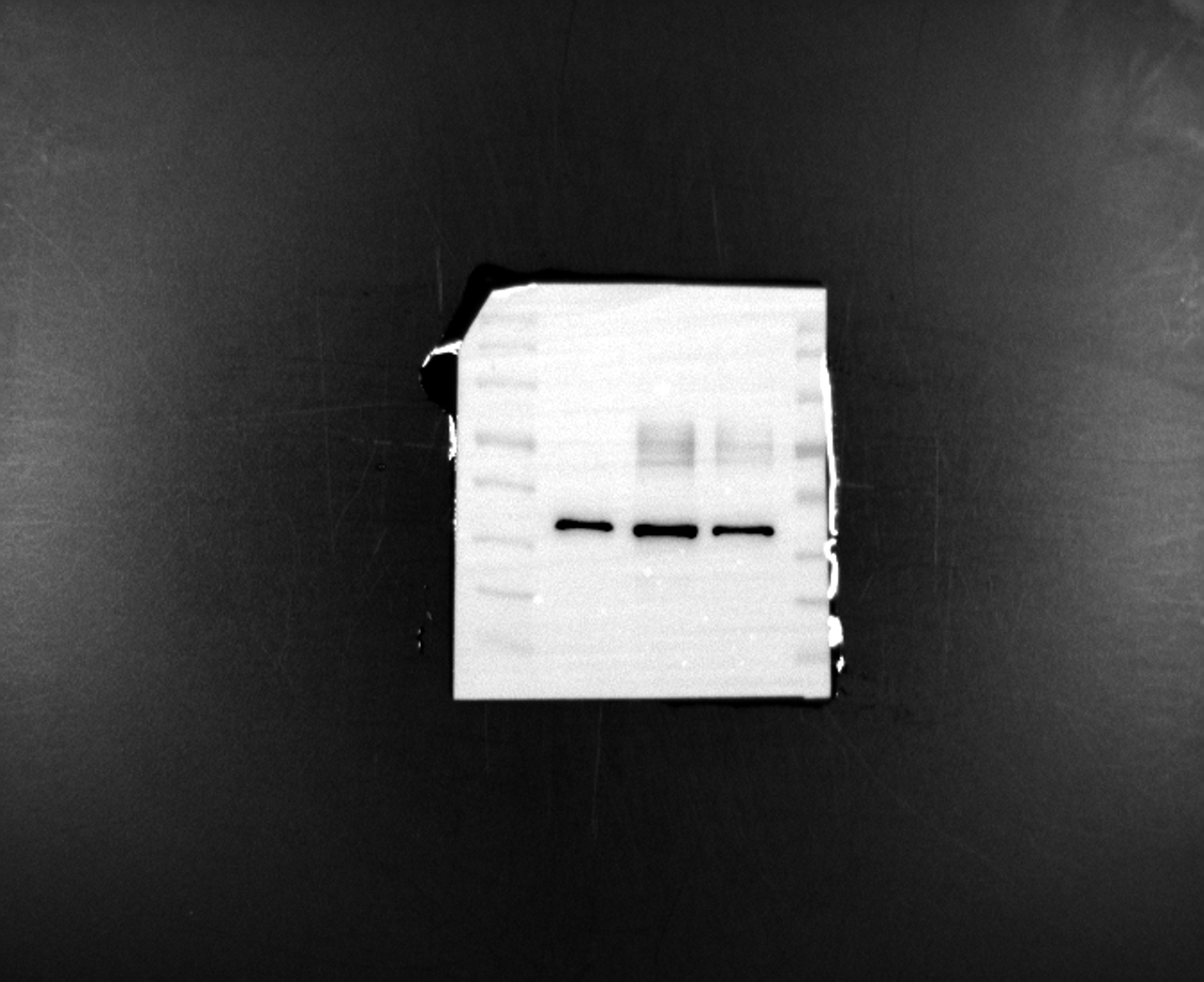

Supplement: Supplementary file 1 [file cancers-16-03028-s001.zip › File S1/For Figure 4/s/549-OE-ACTIN-M.Tif]

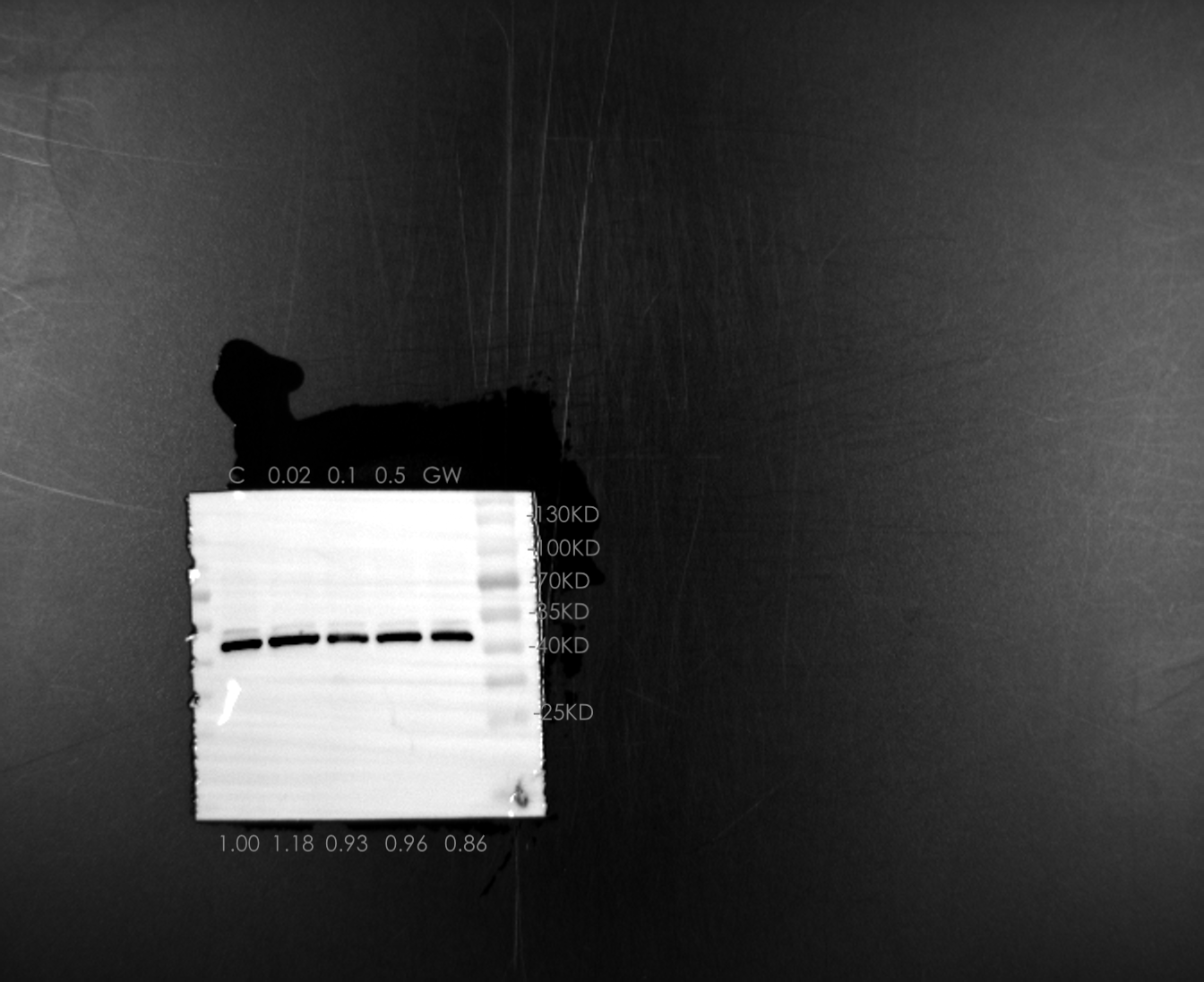

Supplement: Supplementary file 1 [file cancers-16-03028-s001.zip › File S1/For Figure 5/231-C118P-ACTIN-M.Tif]

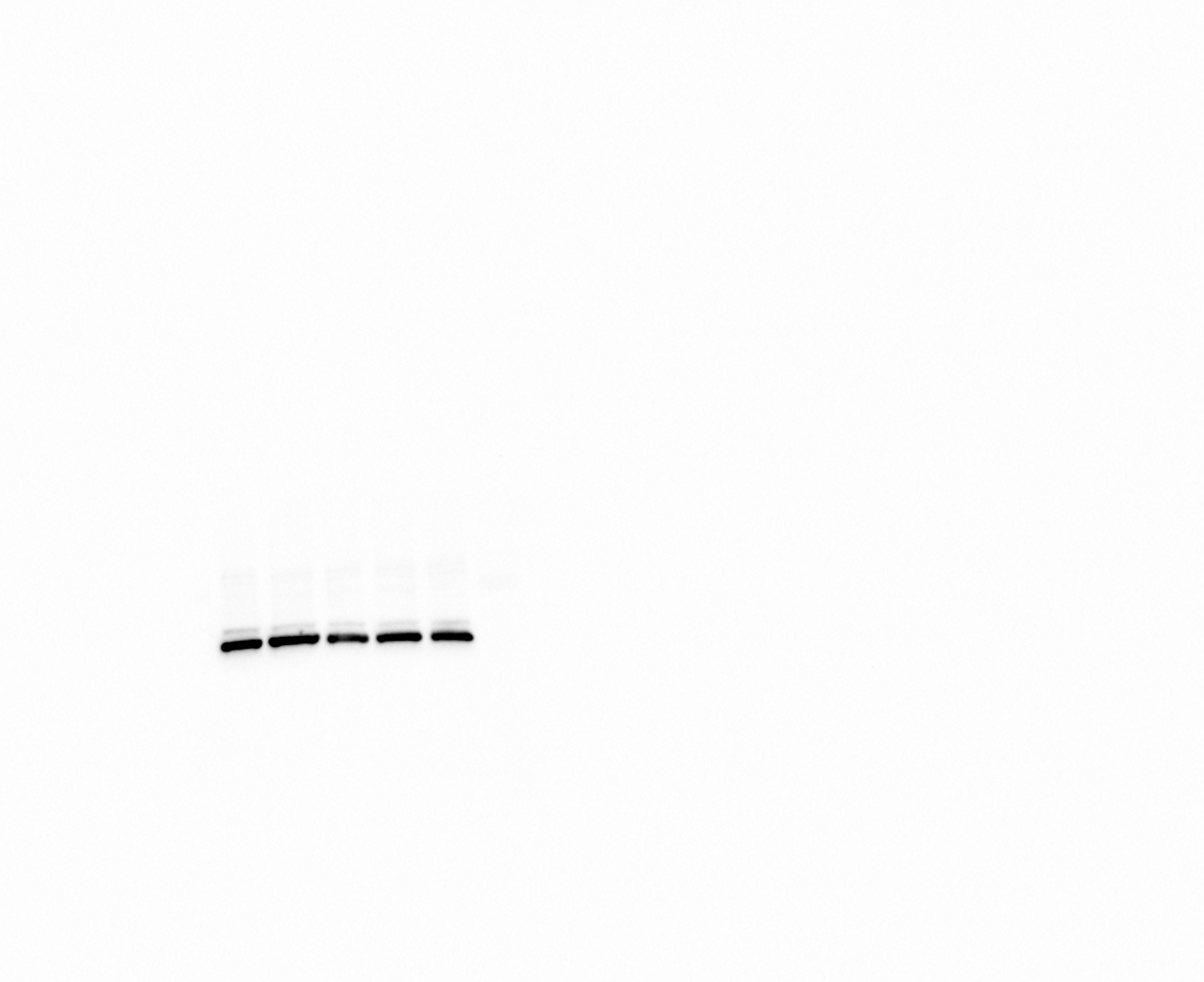

Supplement: Supplementary file 1 [file cancers-16-03028-s001.zip › File S1/For Figure 5/231-C118P-ACTIN.Tif]

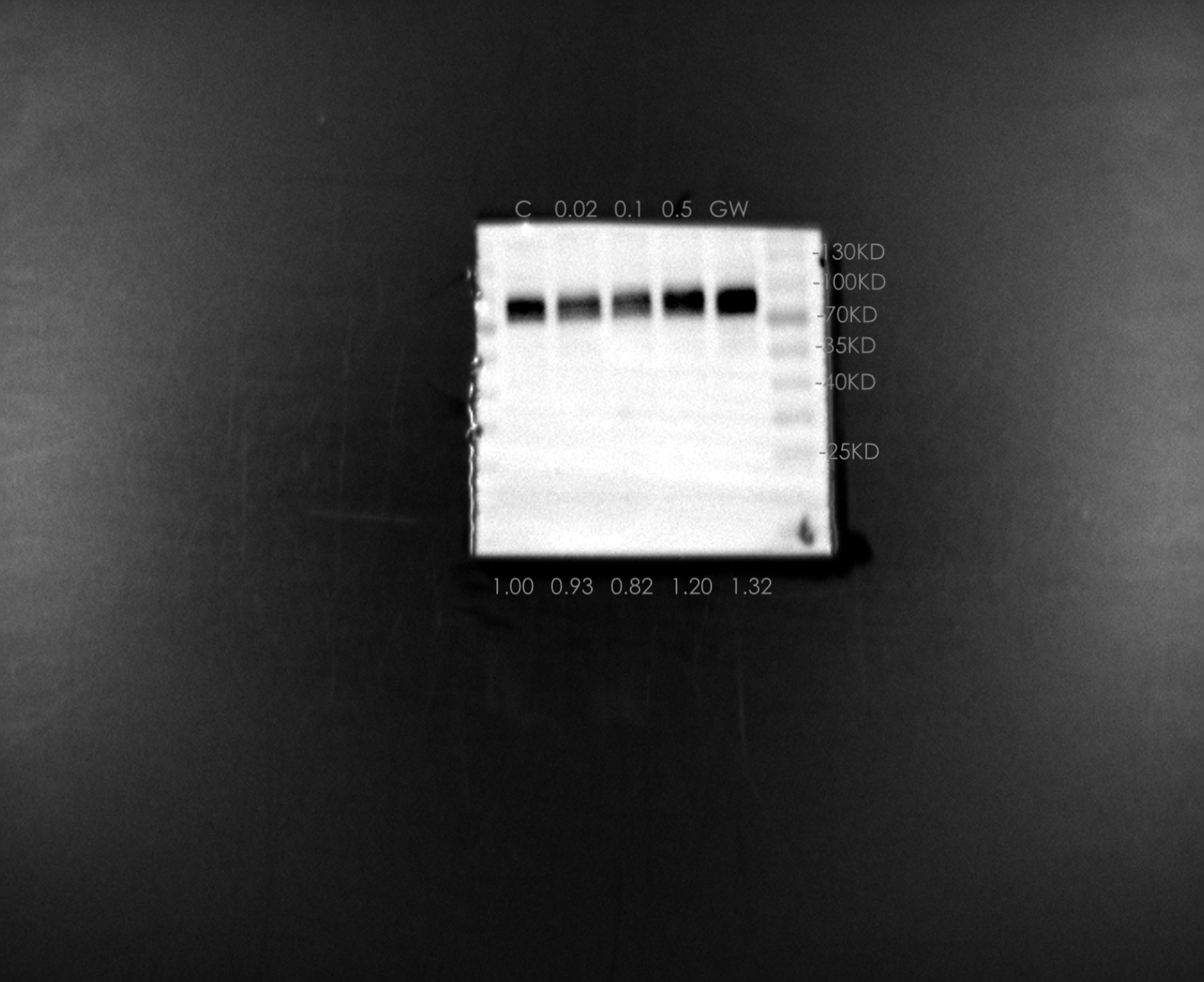

Supplement: Supplementary file 1 [file cancers-16-03028-s001.zip › File S1/For Figure 5/231-C118P-ASCT2-M.Tif]

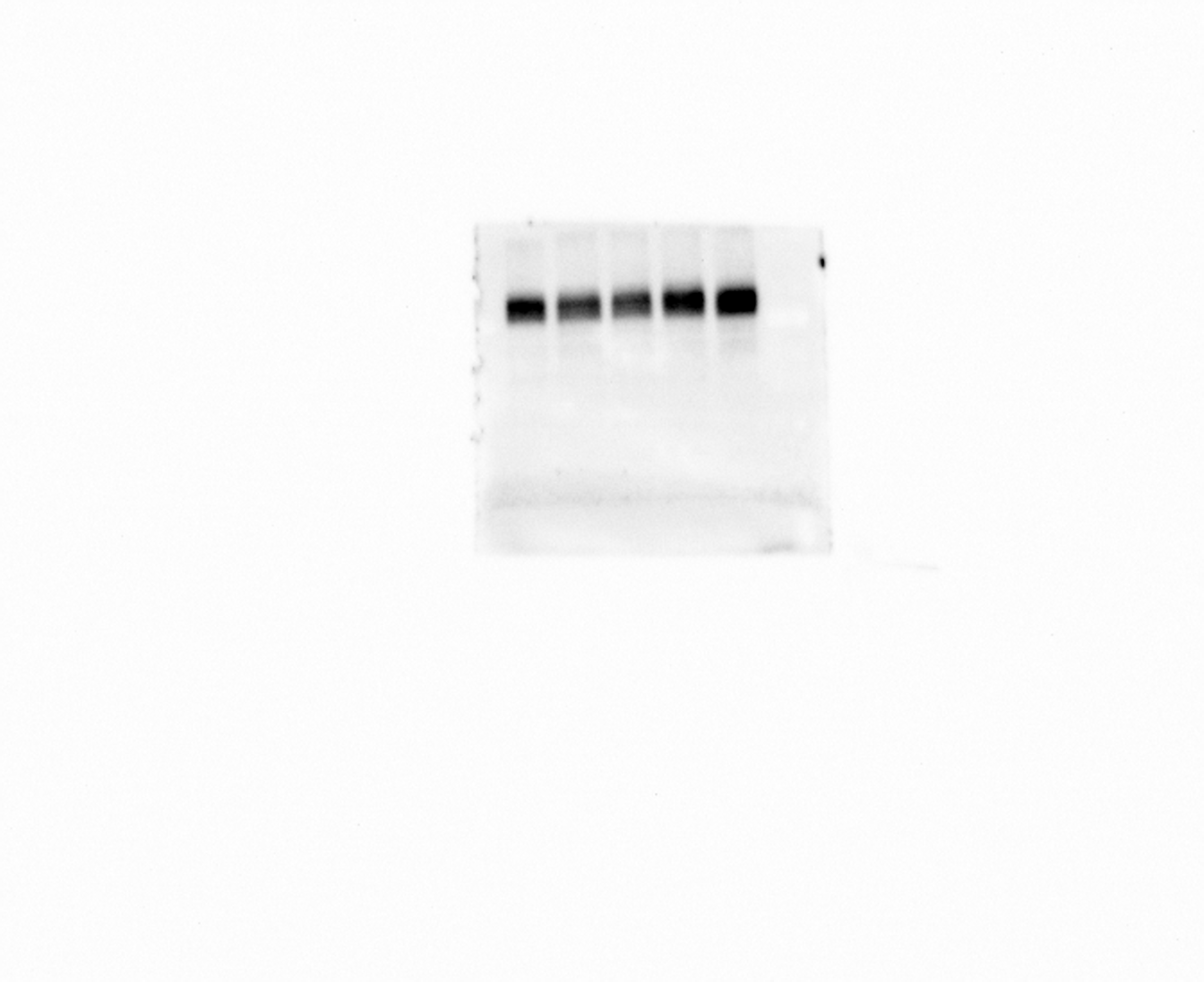

Supplement: Supplementary file 1 [file cancers-16-03028-s001.zip › File S1/For Figure 5/231-C118P-ASCT2.Tif]

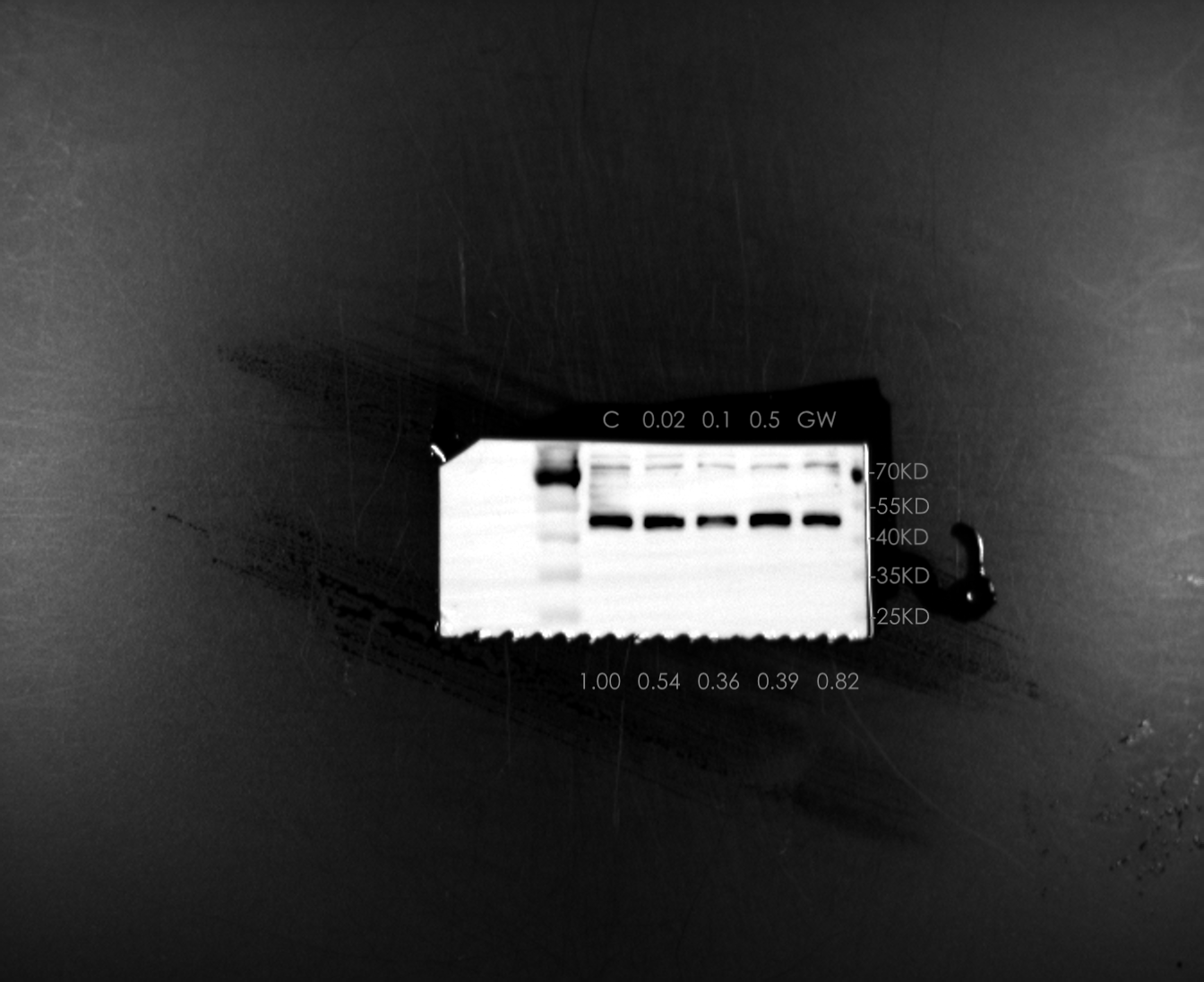

Supplement: Supplementary file 1 [file cancers-16-03028-s001.zip › File S1/For Figure 5/231-C118P-CPT1B-M.Tif]

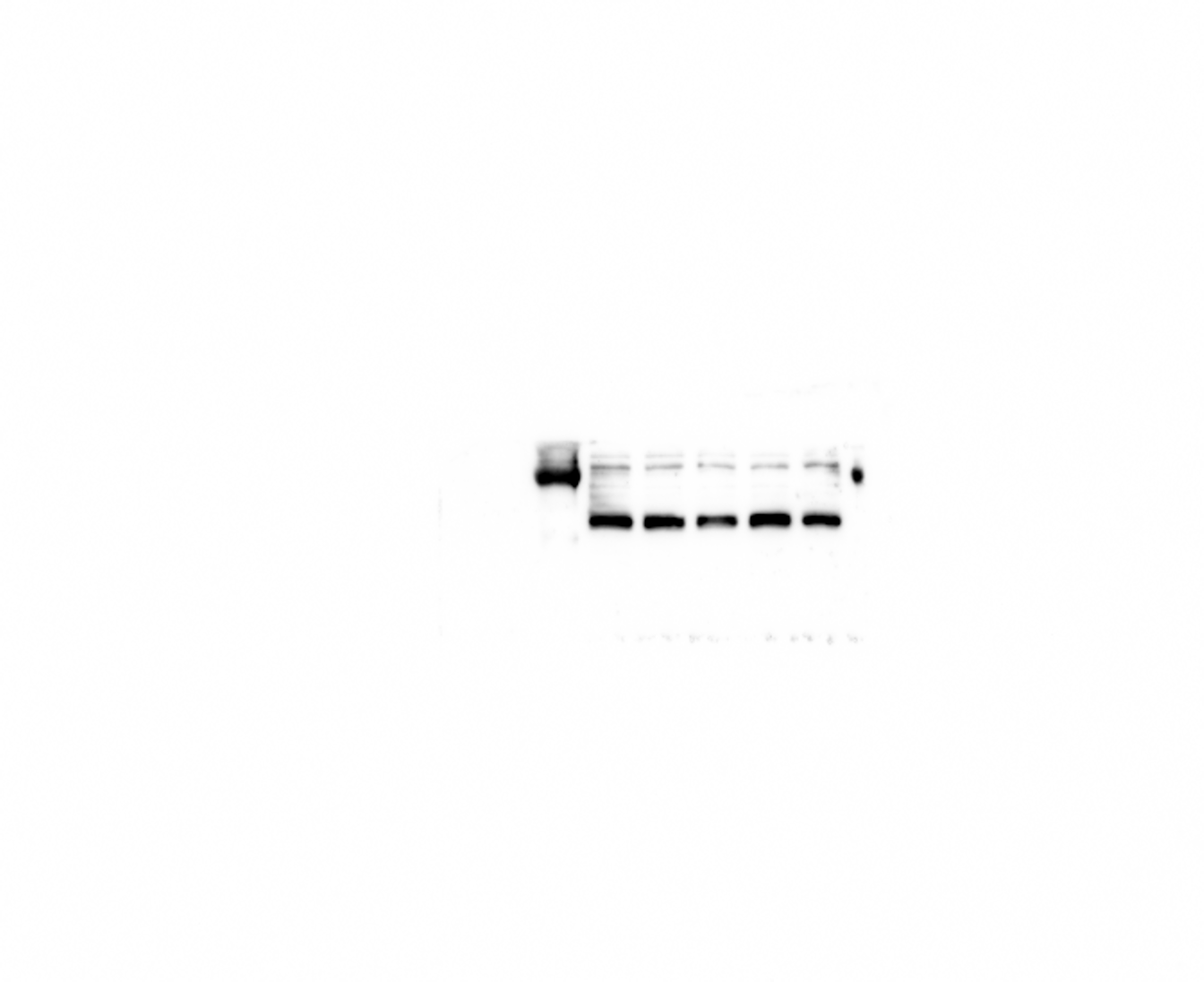

Supplement: Supplementary file 1 [file cancers-16-03028-s001.zip › File S1/For Figure 5/231-C118P-CPT1B.Tif]

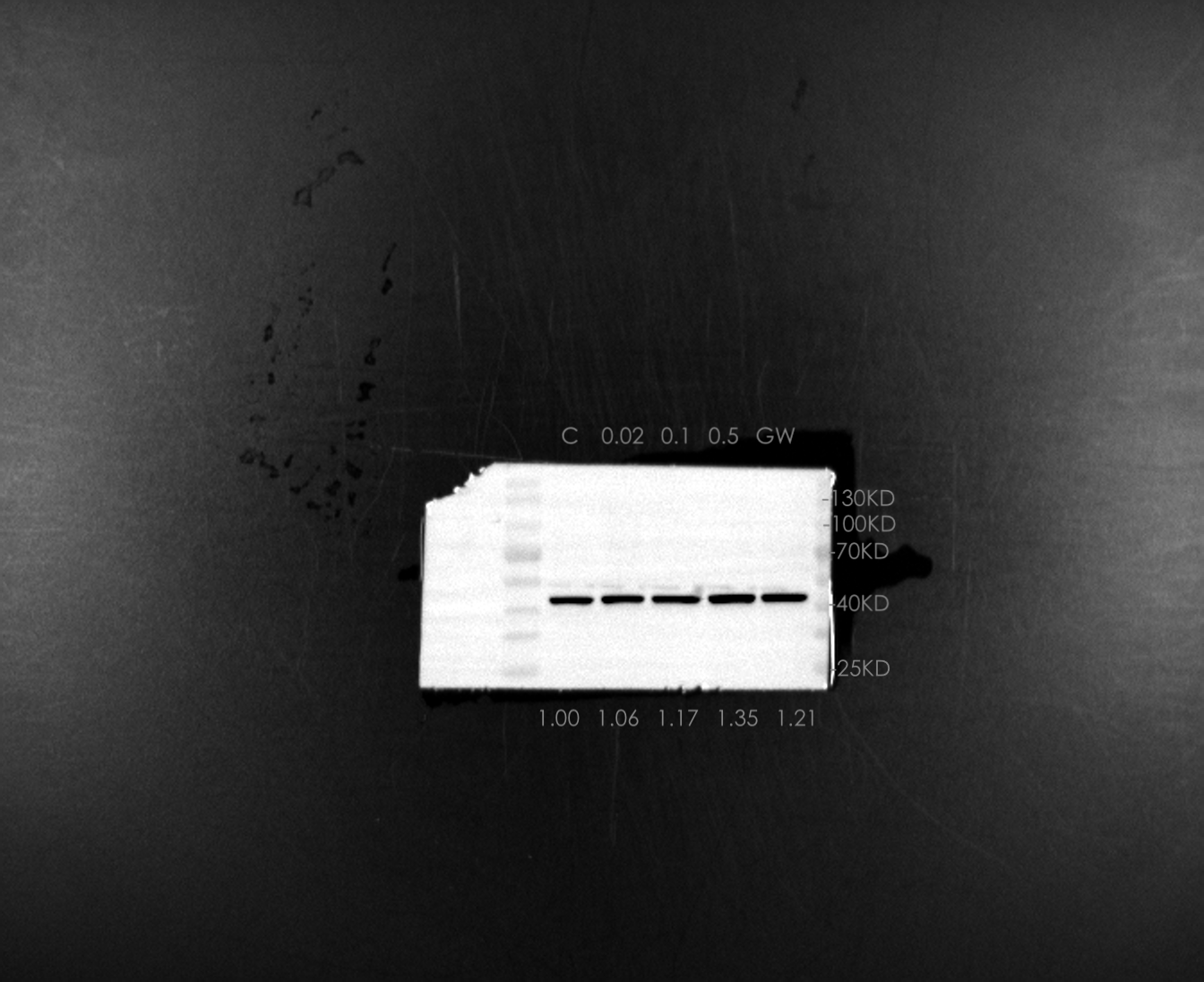

Supplement: Supplementary file 1 [file cancers-16-03028-s001.zip › File S1/For Figure 5/549-C118P-ACTIN-M.Tif]

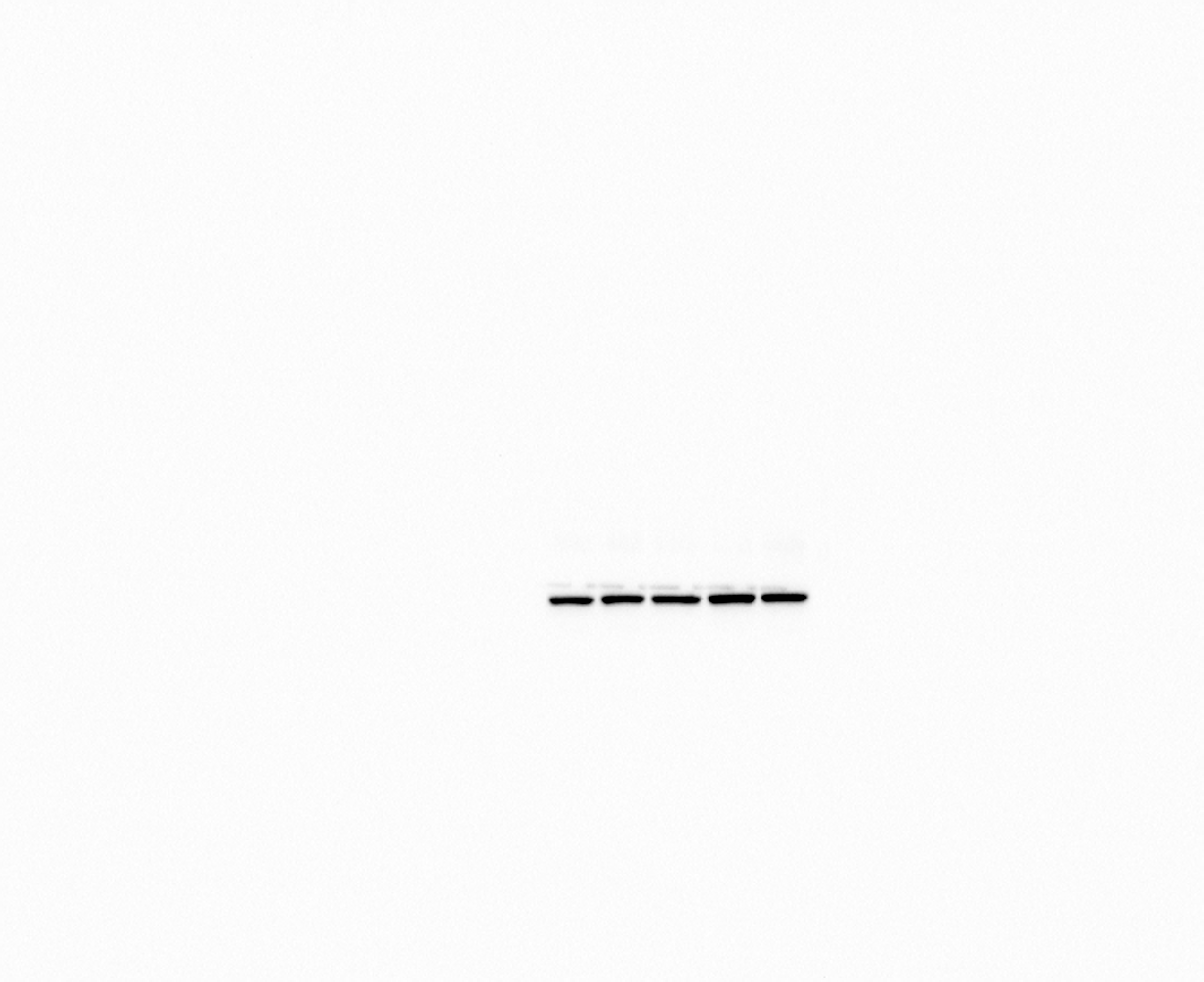

Supplement: Supplementary file 1 [file cancers-16-03028-s001.zip › File S1/For Figure 5/549-C118P-ACTIN.Tif]

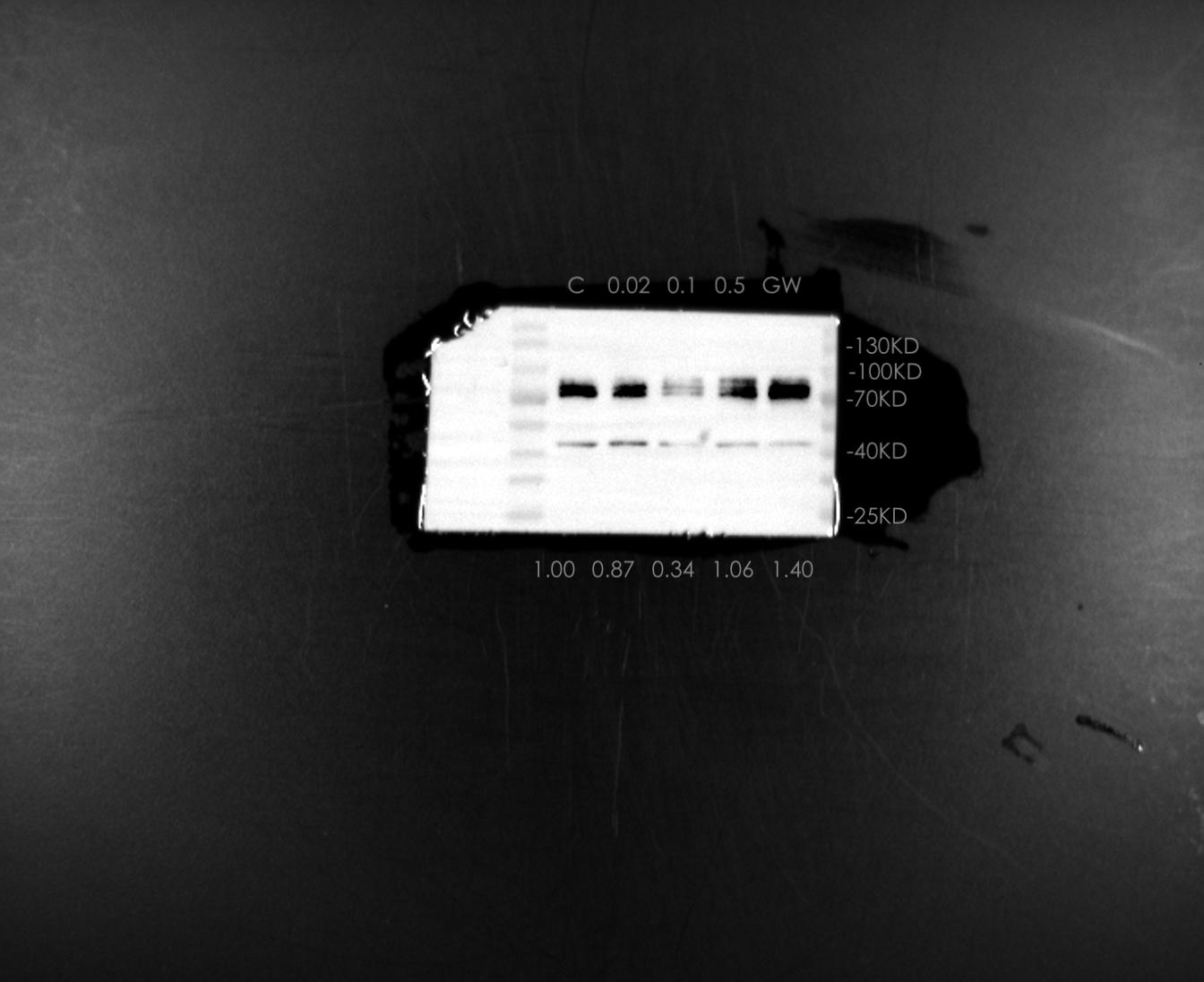

Supplement: Supplementary file 1 [file cancers-16-03028-s001.zip › File S1/For Figure 5/549-C118P-ASCT2-M.Tif]

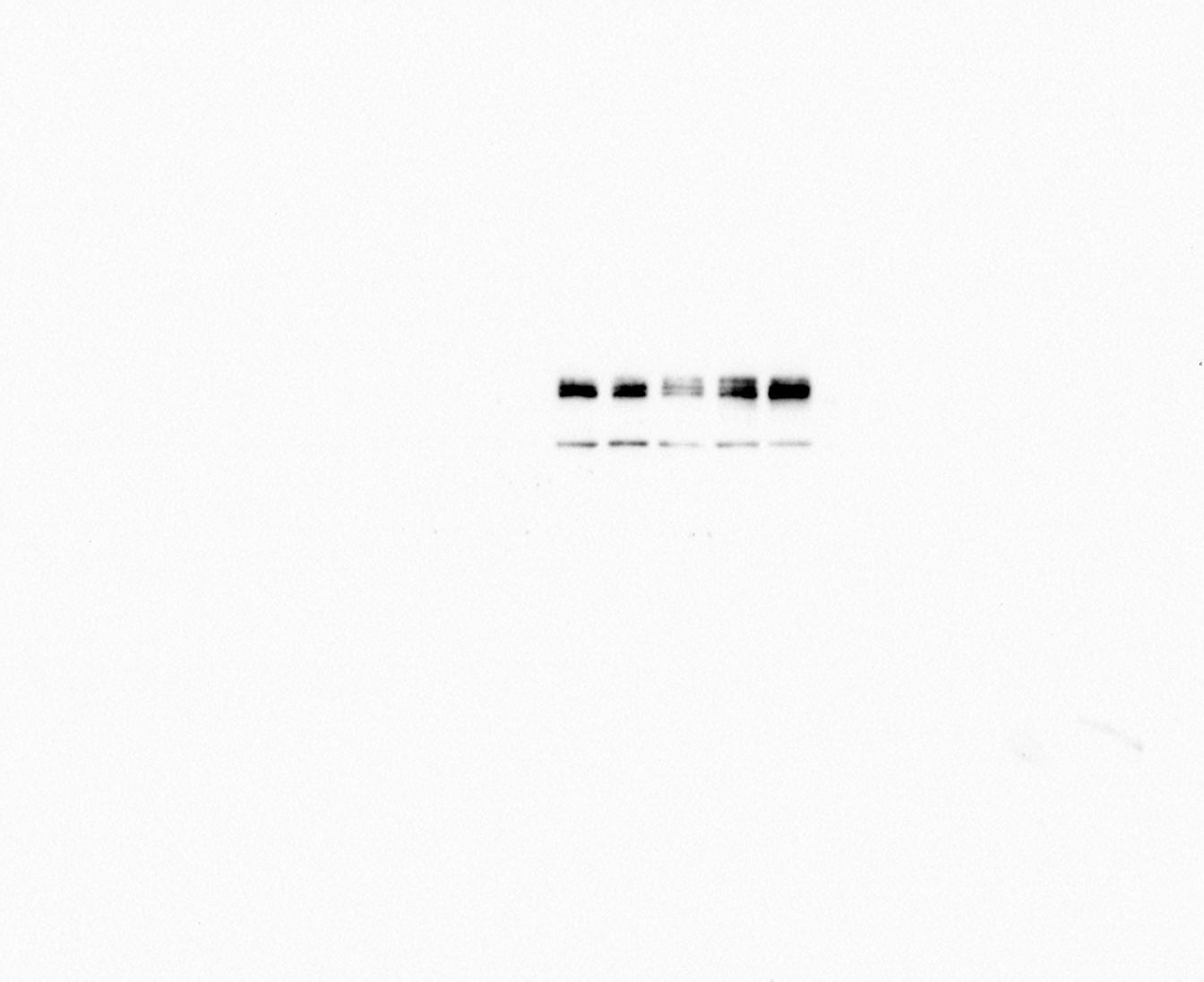

Supplement: Supplementary file 1 [file cancers-16-03028-s001.zip › File S1/For Figure 5/549-C118P-ASCT2.Tif]

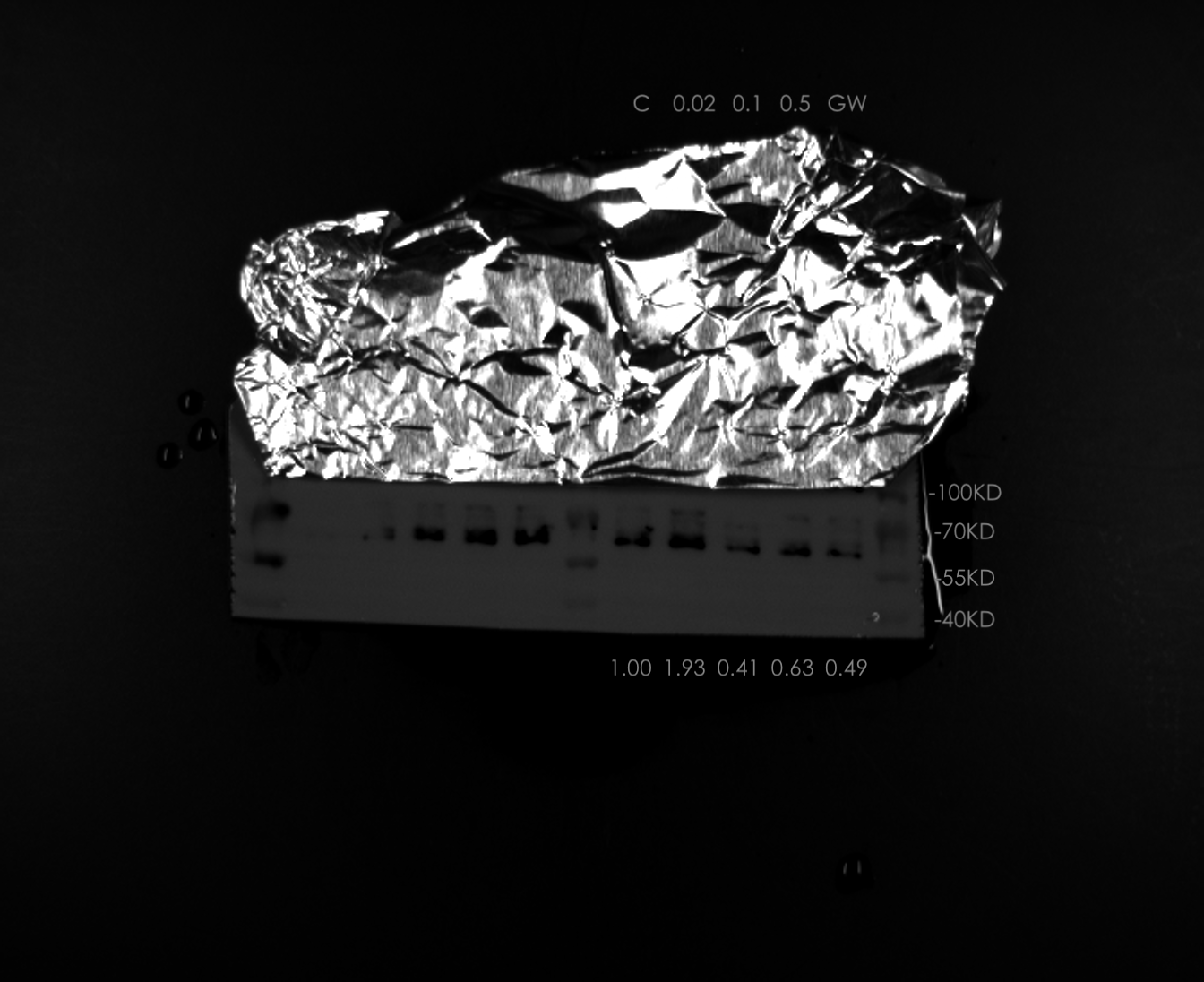

Supplement: Supplementary file 1 [file cancers-16-03028-s001.zip › File S1/For Figure 5/549-C118P-CPT1B-3M-M.Tif]

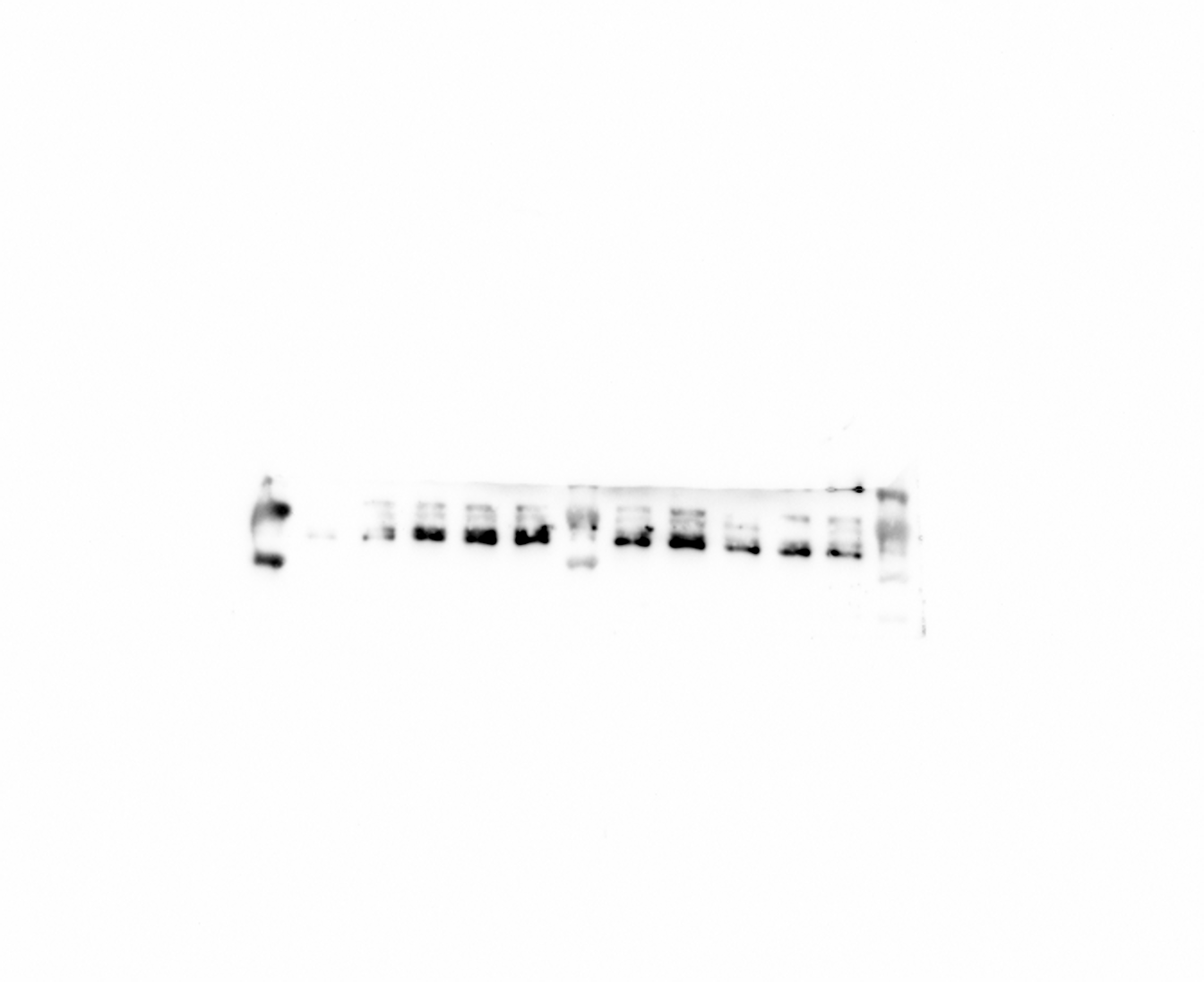

Supplement: Supplementary file 1 [file cancers-16-03028-s001.zip › File S1/For Figure 5/549-C118P-CPTB-3M.Tif]

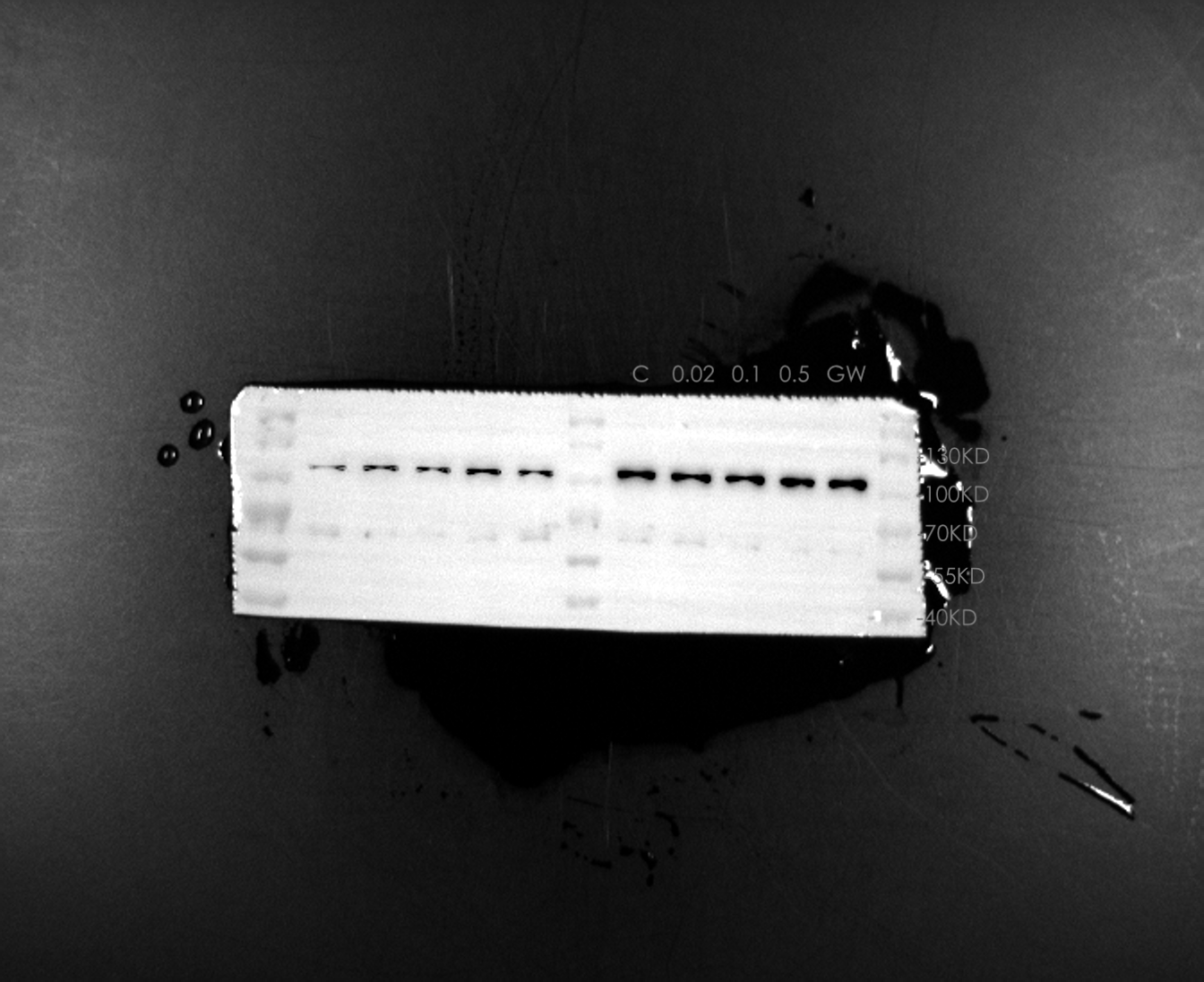

Supplement: Supplementary file 1 [file cancers-16-03028-s001.zip › File S1/For Figure 5/549-C118P-NOT-CPT1B-M.Tif]

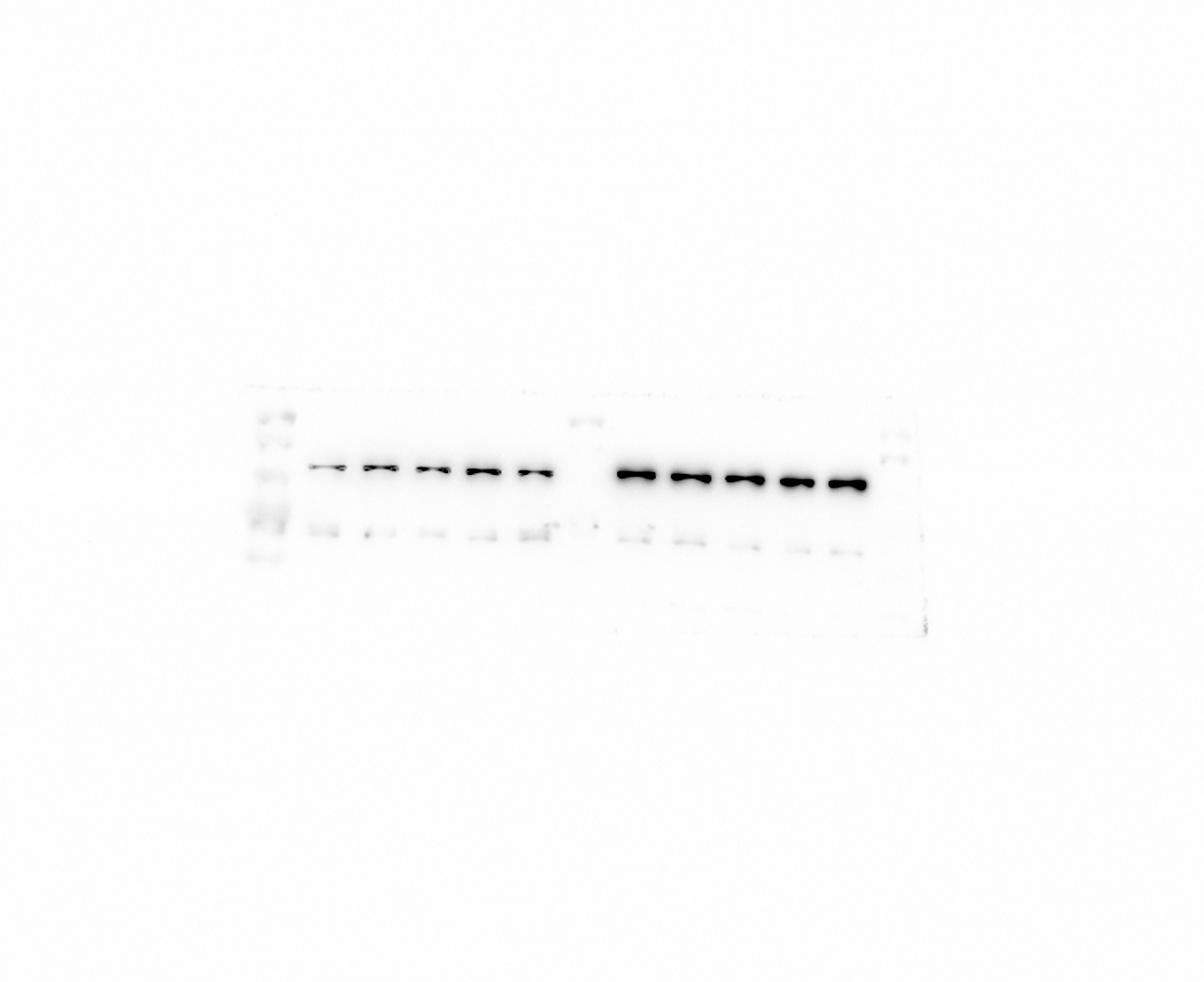

Supplement: Supplementary file 1 [file cancers-16-03028-s001.zip › File S1/For Figure 5/549-C118P-NOT-CPT1B.Tif]

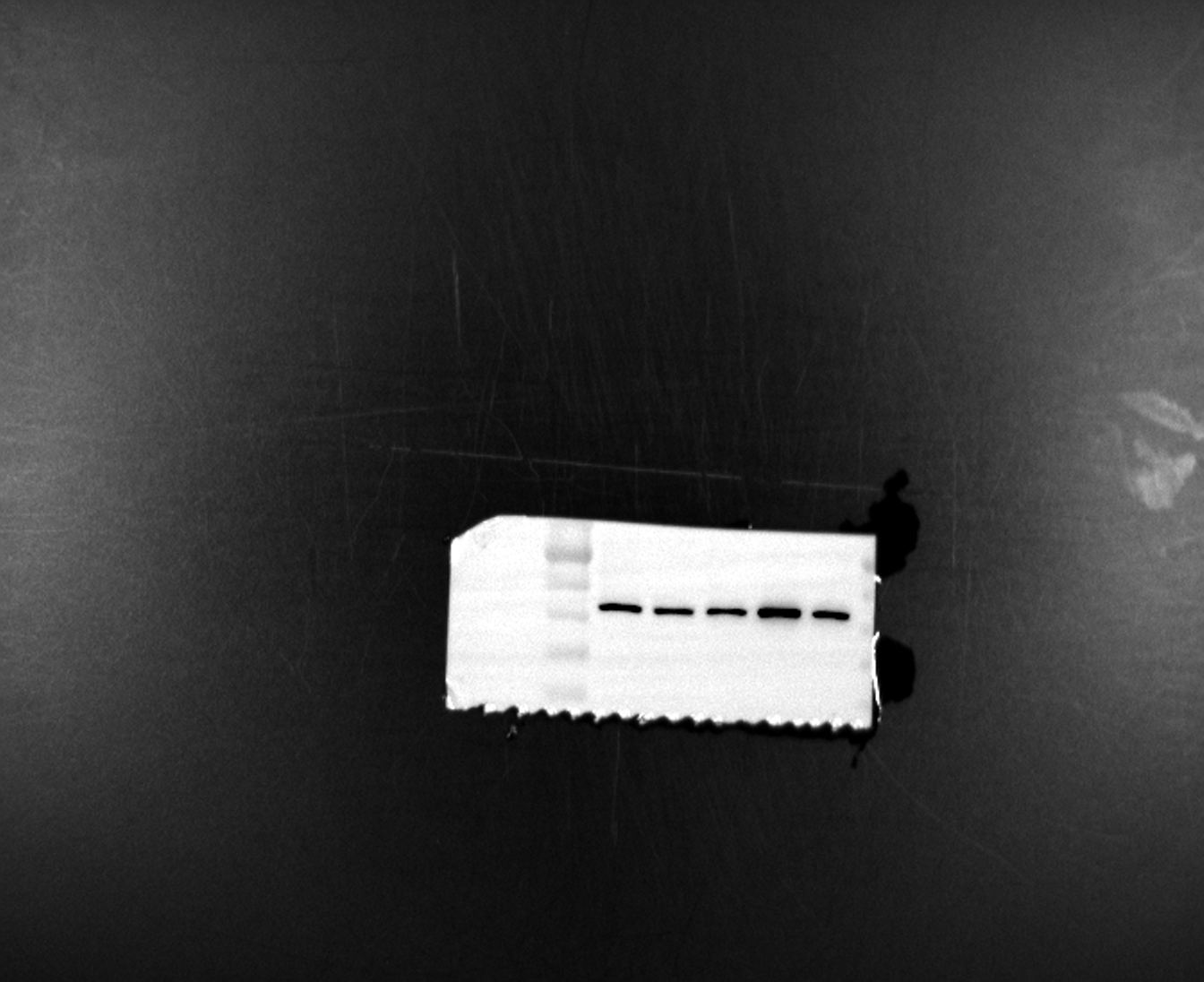

Supplement: Supplementary file 1 [file cancers-16-03028-s001.zip › File S1/For Figure 5/S/231-C118P-CPT1B-ACTIN-M.Tif]

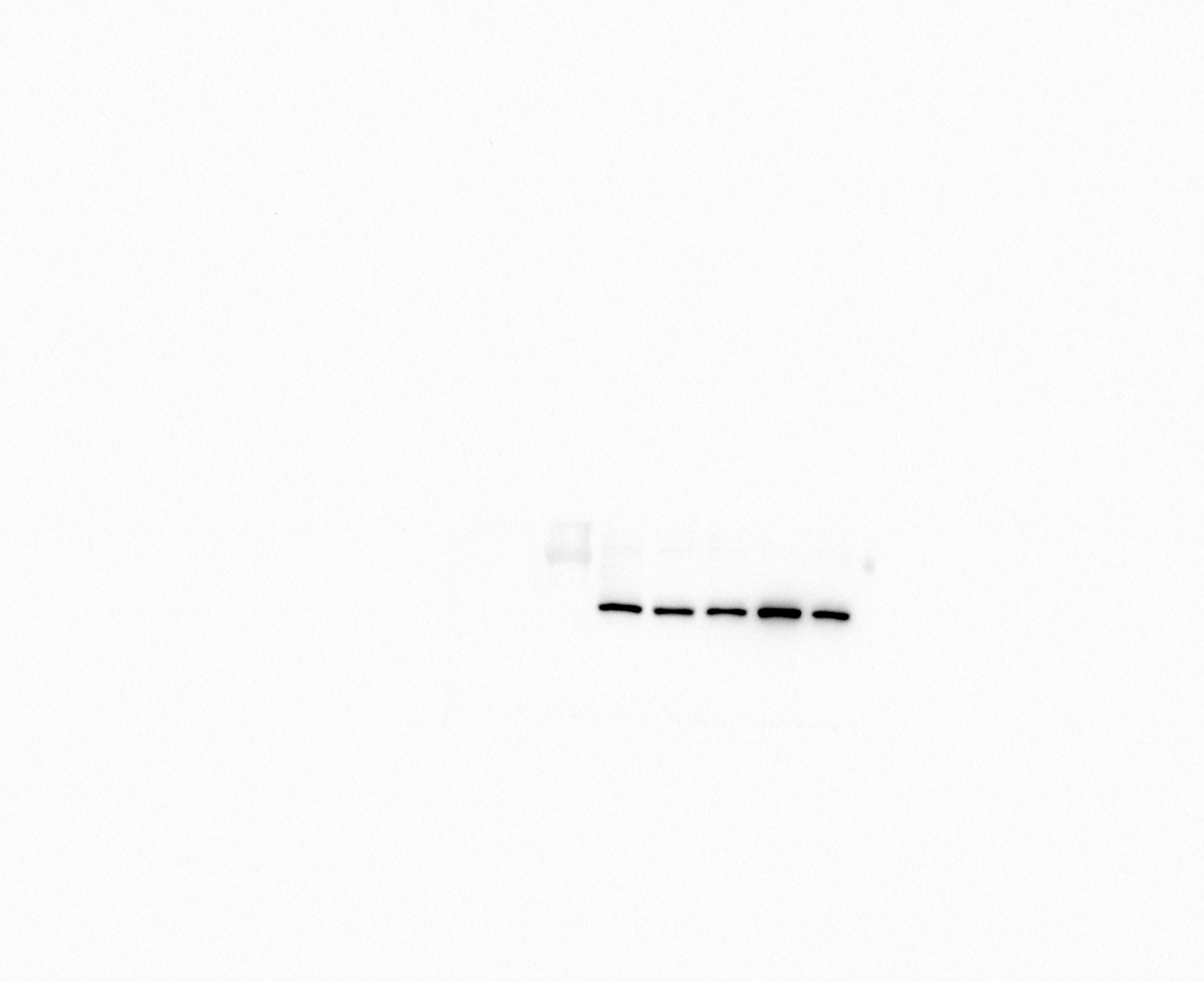

Supplement: Supplementary file 1 [file cancers-16-03028-s001.zip › File S1/For Figure 5/S/231-C118P-CPT1B-ACTIN.Tif]

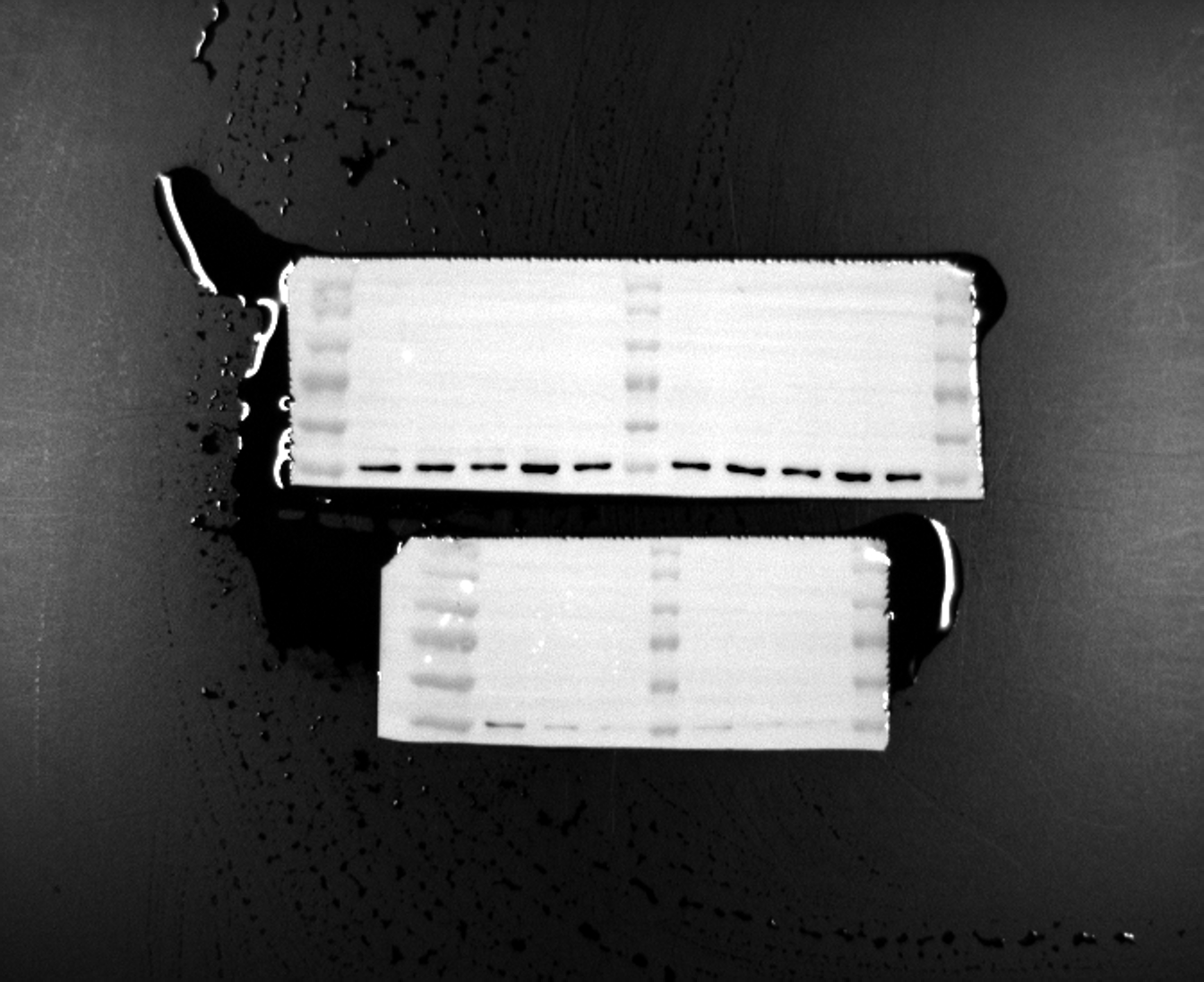

Supplement: Supplementary file 1 [file cancers-16-03028-s001.zip › File S1/For Figure 5/S/549-C118P-CPT1B-ACTIN-M.Tif]

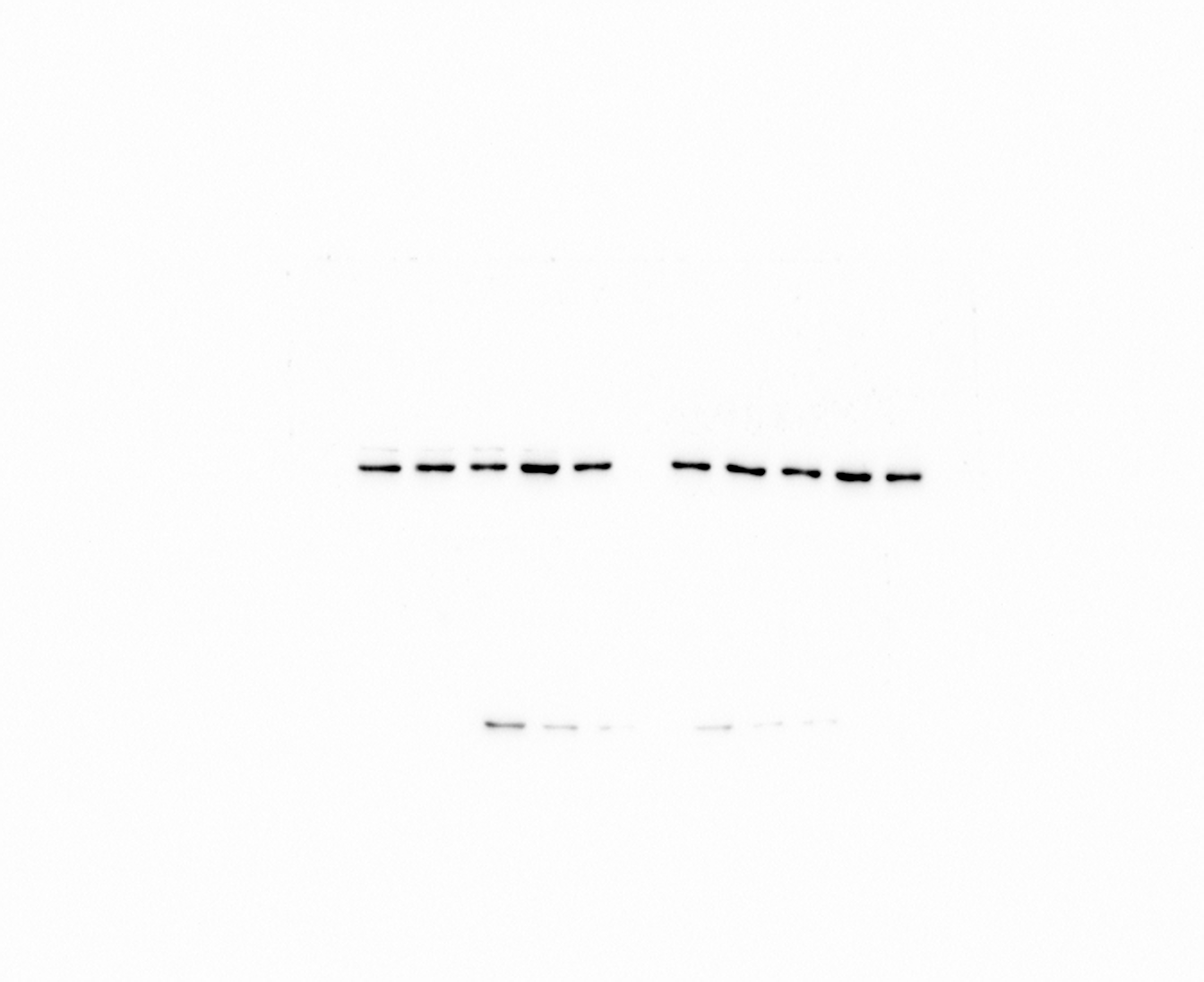

Supplement: Supplementary file 1 [file cancers-16-03028-s001.zip › File S1/For Figure 5/S/549-C118P-CPT1B-ACTIN.Tif]

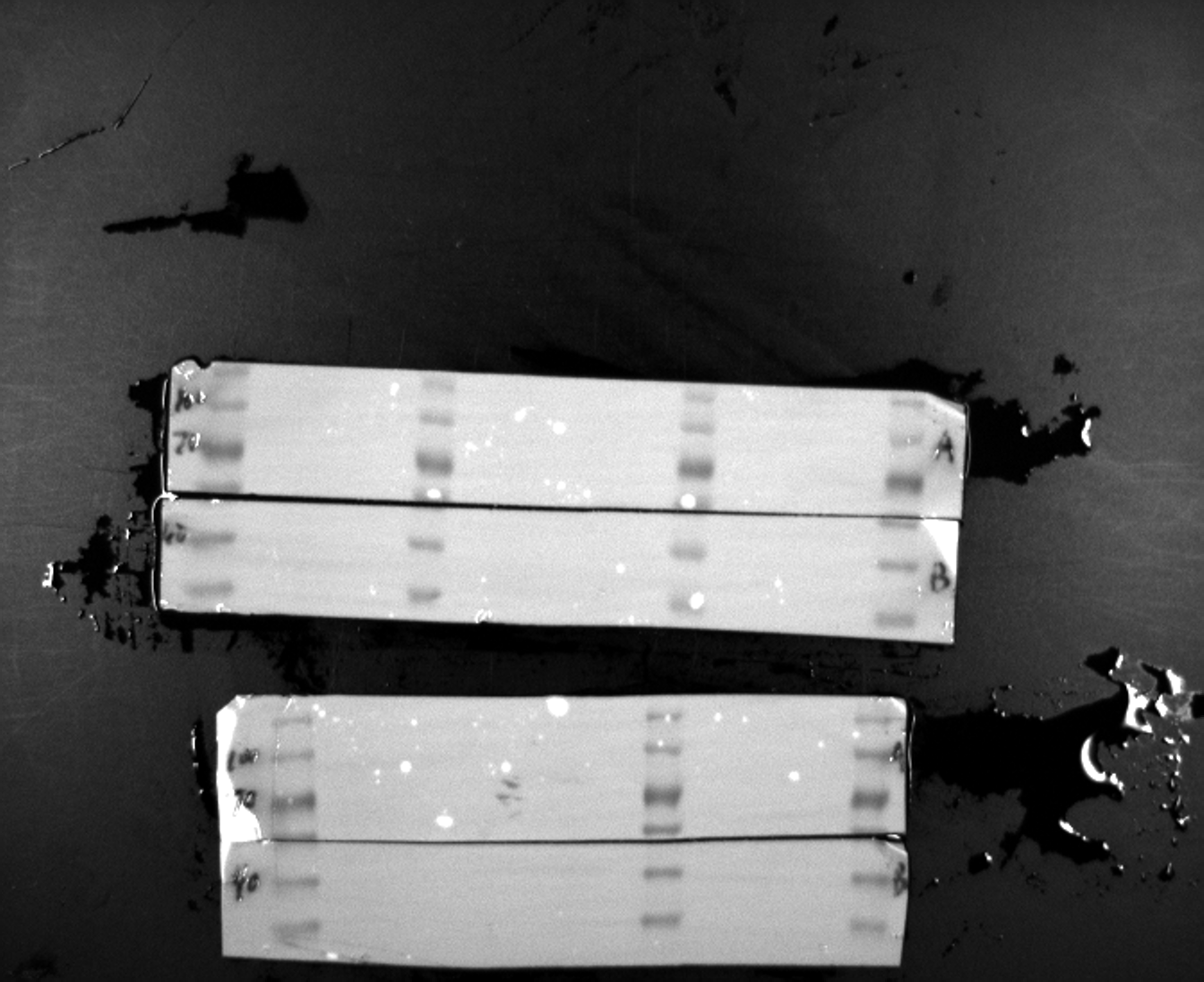

Supplement: Supplementary file 1 [file cancers-16-03028-s001.zip › File S1/Supplement/231/231-A.Tif]

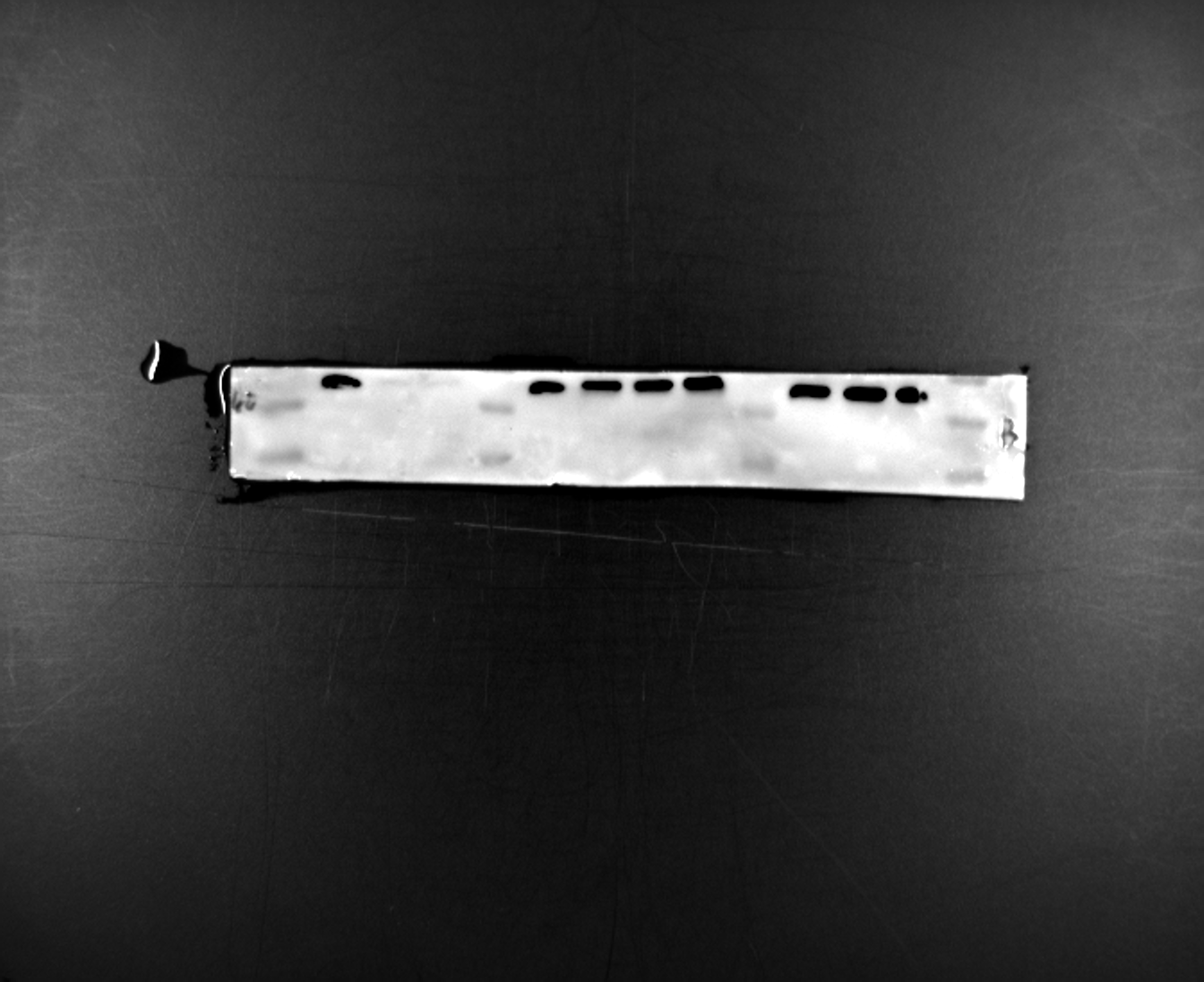

Supplement: Supplementary file 1 [file cancers-16-03028-s001.zip › File S1/Supplement/231/231-ACTIN-10S.Tif]

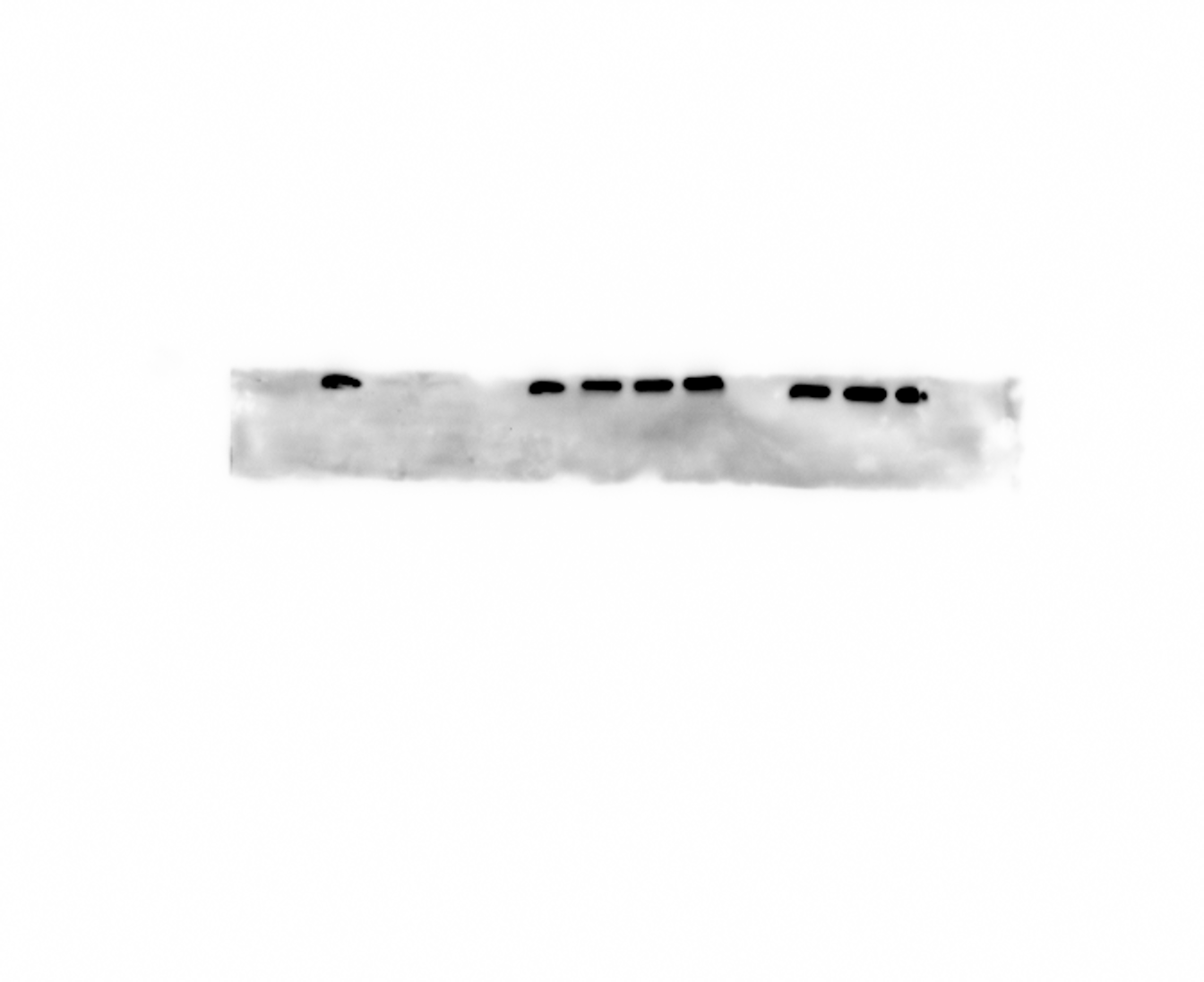

Supplement: Supplementary file 1 [file cancers-16-03028-s001.zip › File S1/Supplement/231/231-ACTIN.Tif]

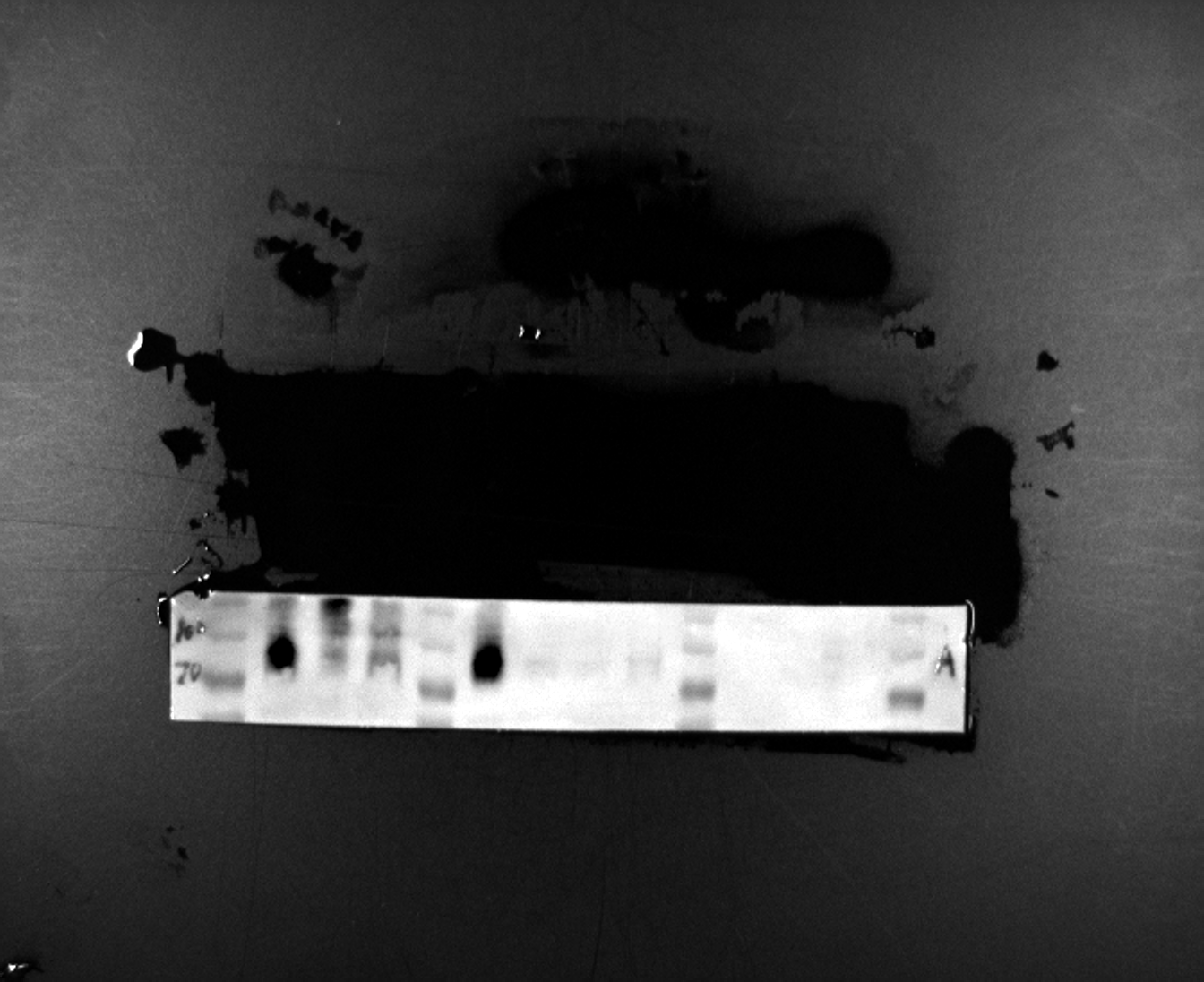

Supplement: Supplementary file 1 [file cancers-16-03028-s001.zip › File S1/Supplement/231/231-ASCT2-30S-M.Tif]

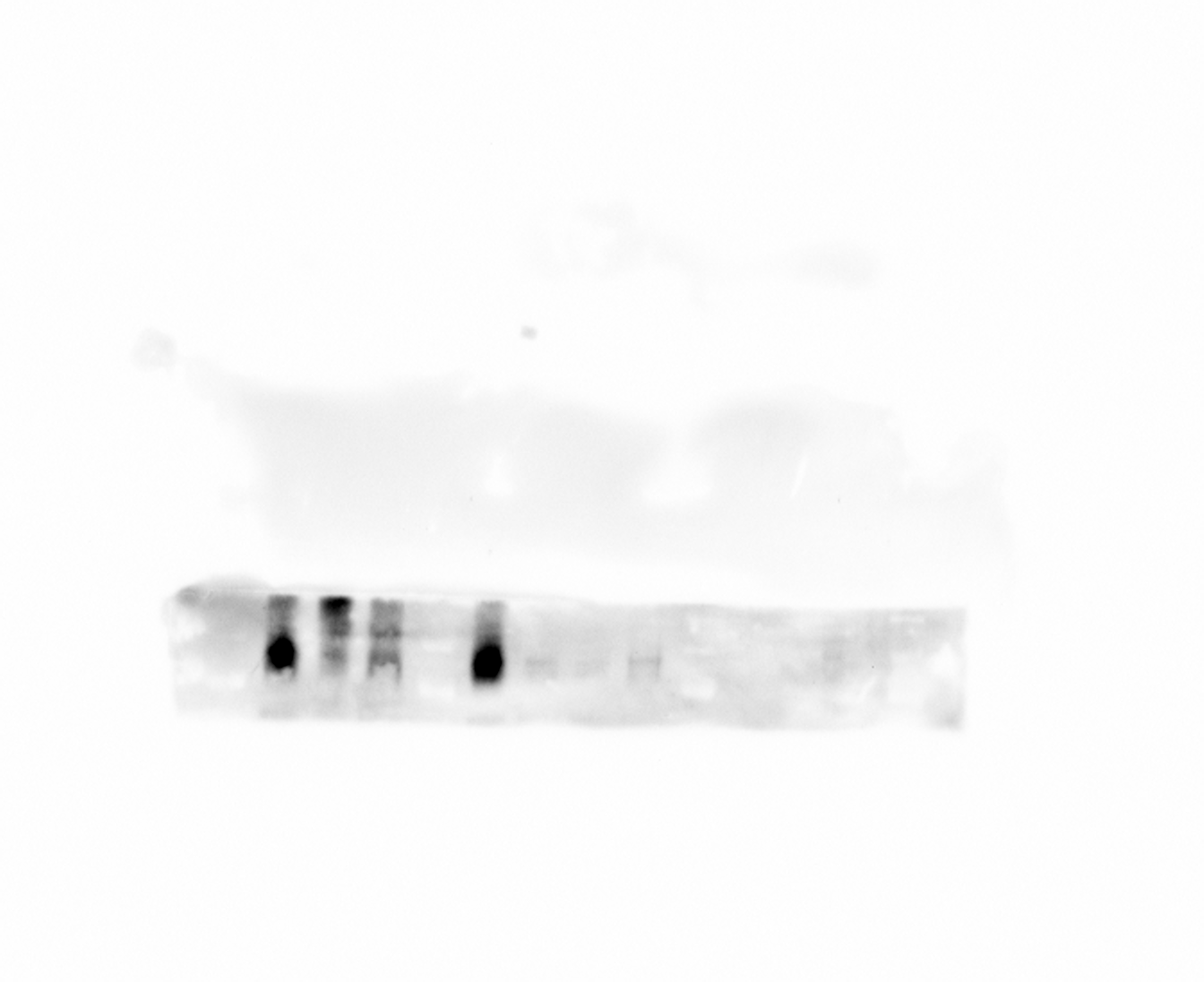

Supplement: Supplementary file 1 [file cancers-16-03028-s001.zip › File S1/Supplement/231/231-ASCT2-30S.Tif]

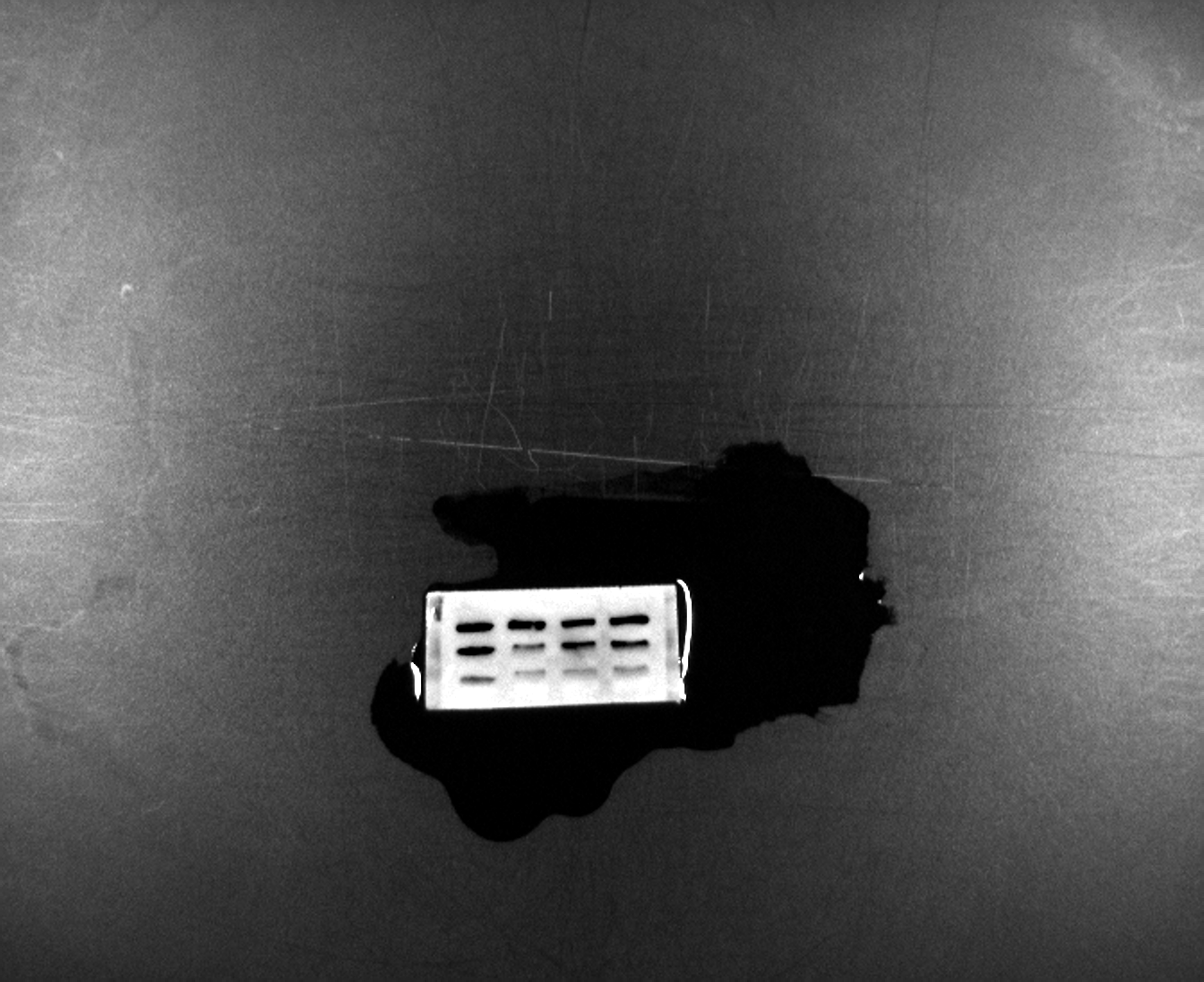

Supplement: Supplementary file 1 [file cancers-16-03028-s001.zip › File S1/Supplement/231/231-SI-CPT1B-10S-M.Tif]

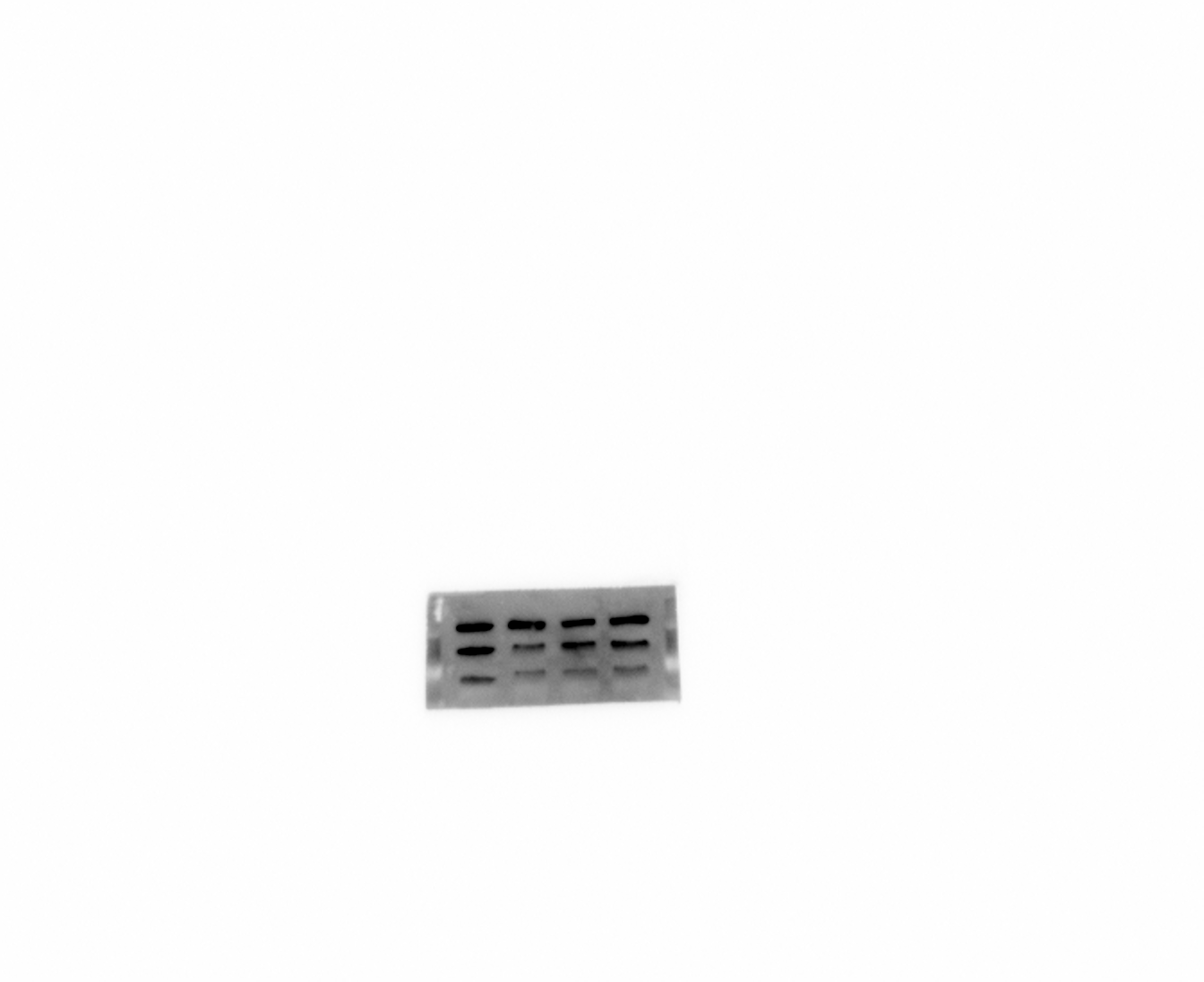

Supplement: Supplementary file 1 [file cancers-16-03028-s001.zip › File S1/Supplement/231/231-SI-CPT1B-10S.Tif]

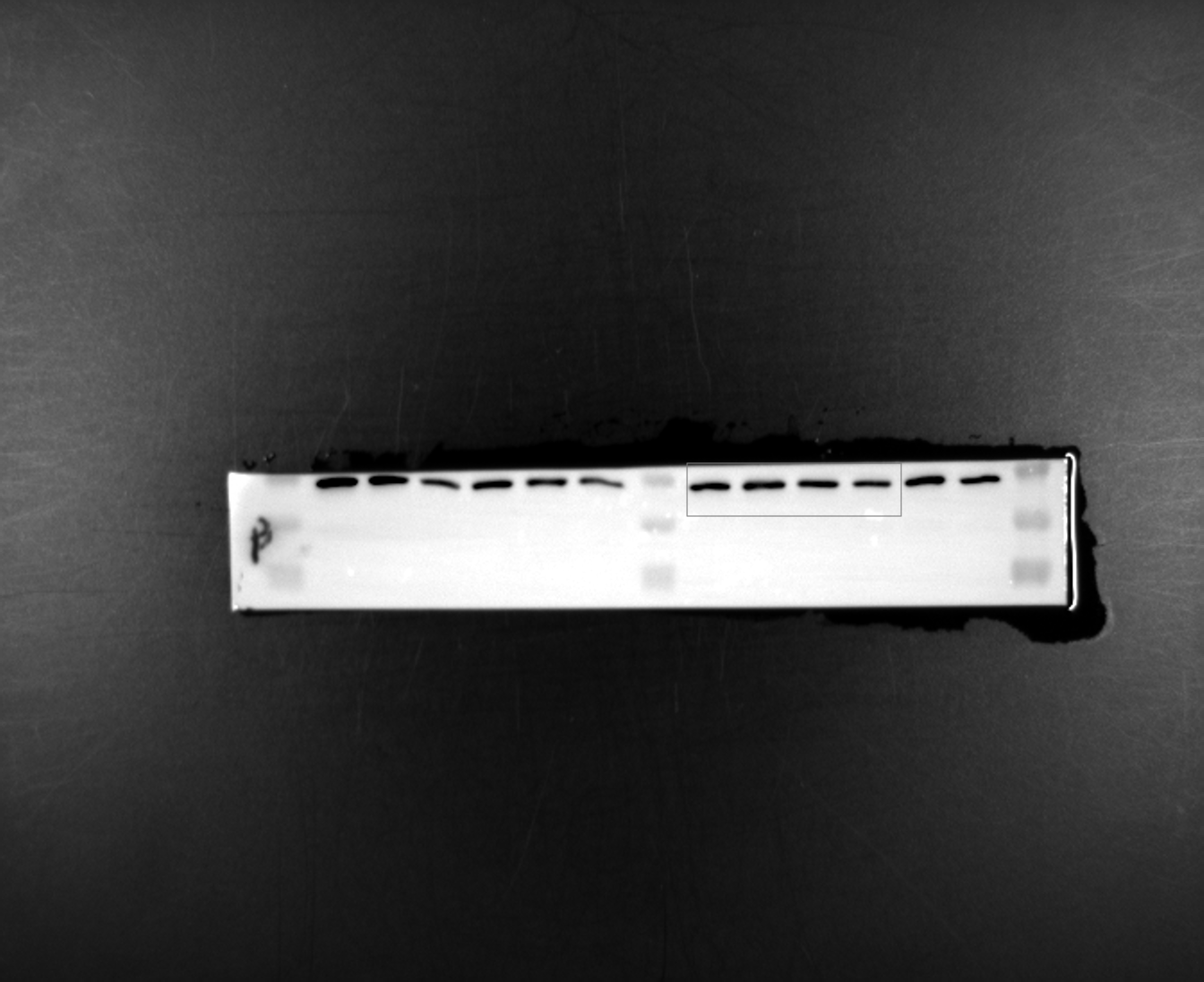

Supplement: Supplementary file 1 [file cancers-16-03028-s001.zip › File S1/Supplement/549/549-ACTIN-10S-M.Tif]

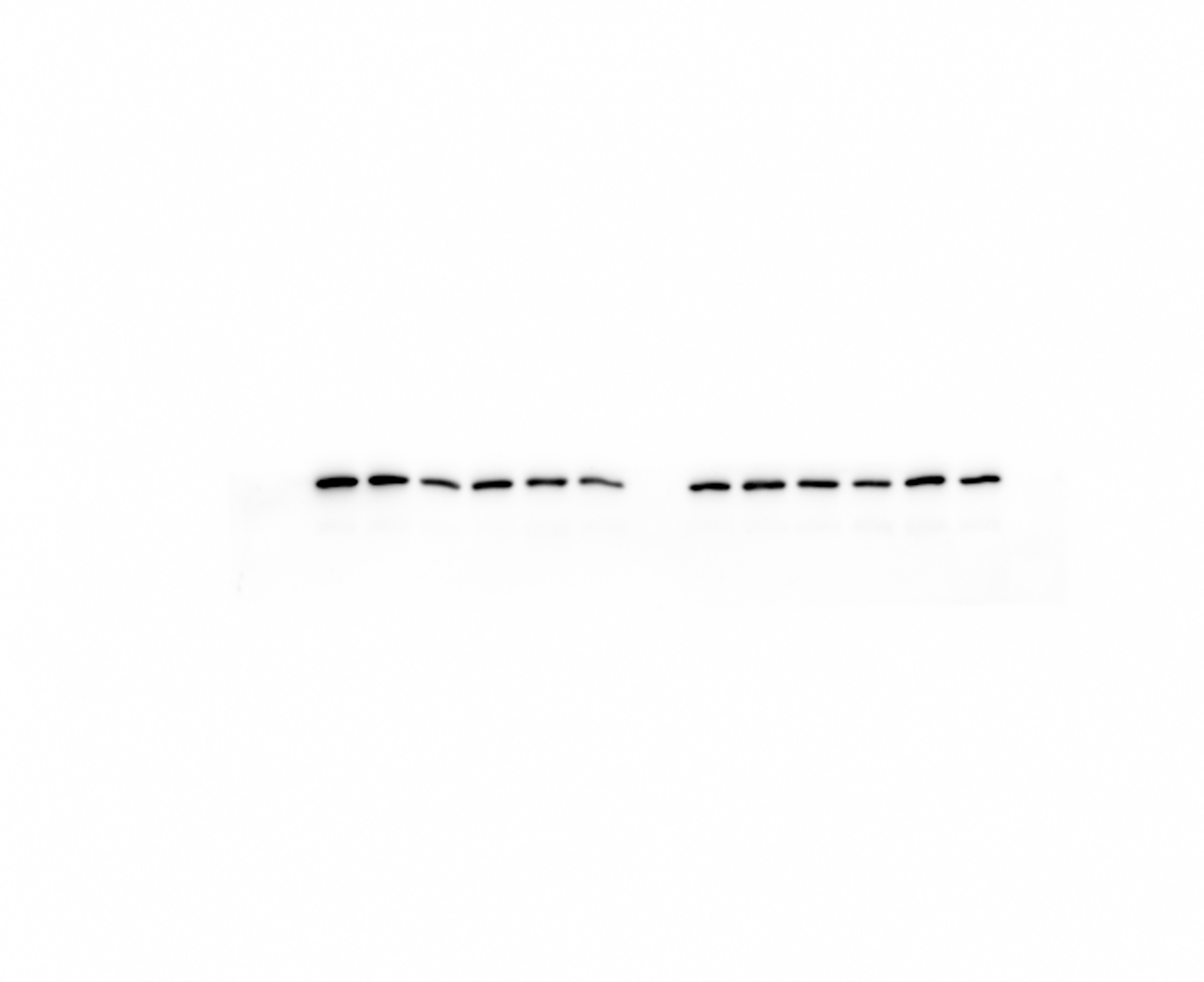

Supplement: Supplementary file 1 [file cancers-16-03028-s001.zip › File S1/Supplement/549/549-ACTIN-10S.Tif]

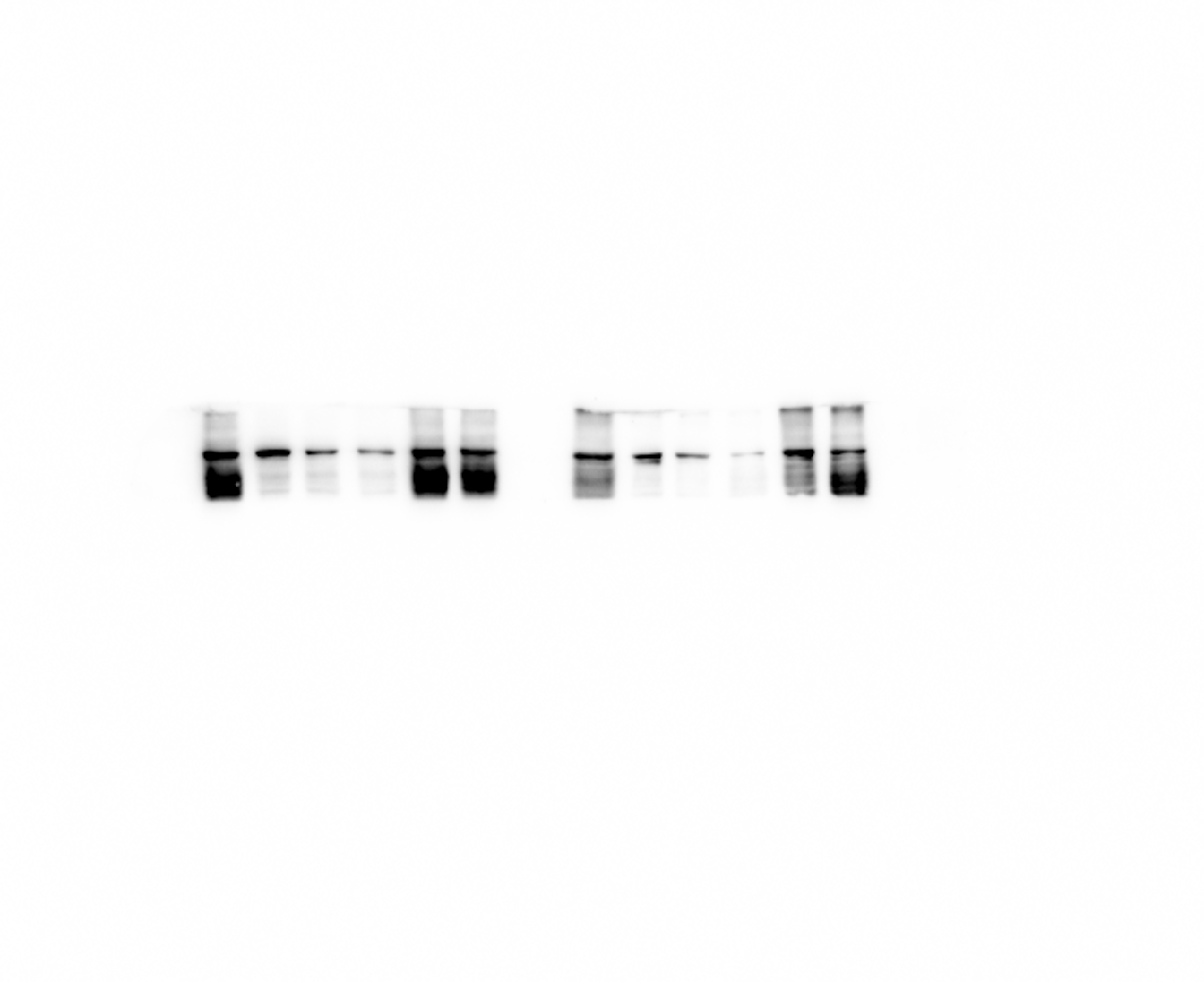

Supplement: Supplementary file 1 [file cancers-16-03028-s001.zip › File S1/Supplement/549/549-ASCT2-10S.Tif]

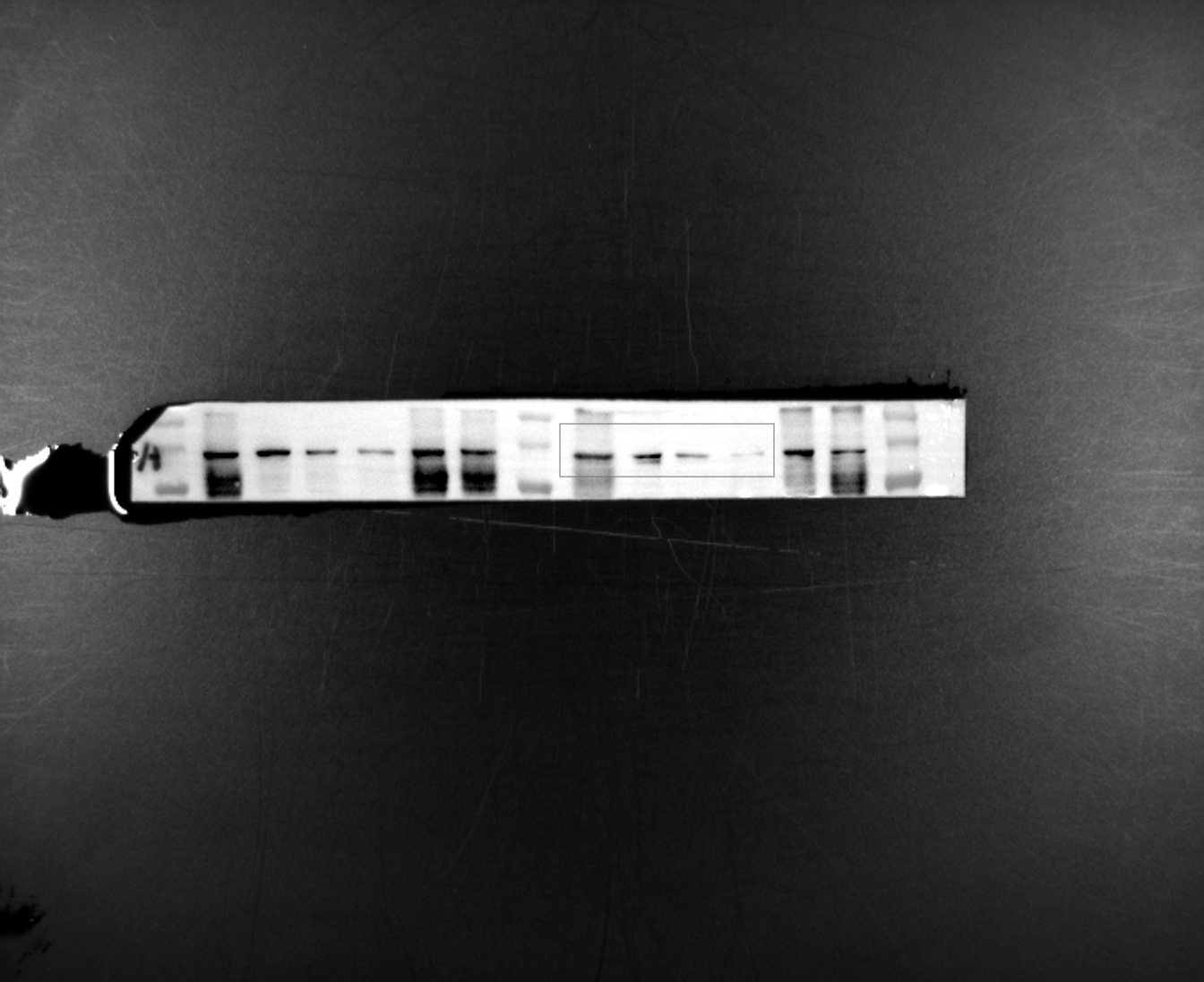

Supplement: Supplementary file 1 [file cancers-16-03028-s001.zip › File S1/Supplement/549/549-ASCT2-1S-M.Tif]

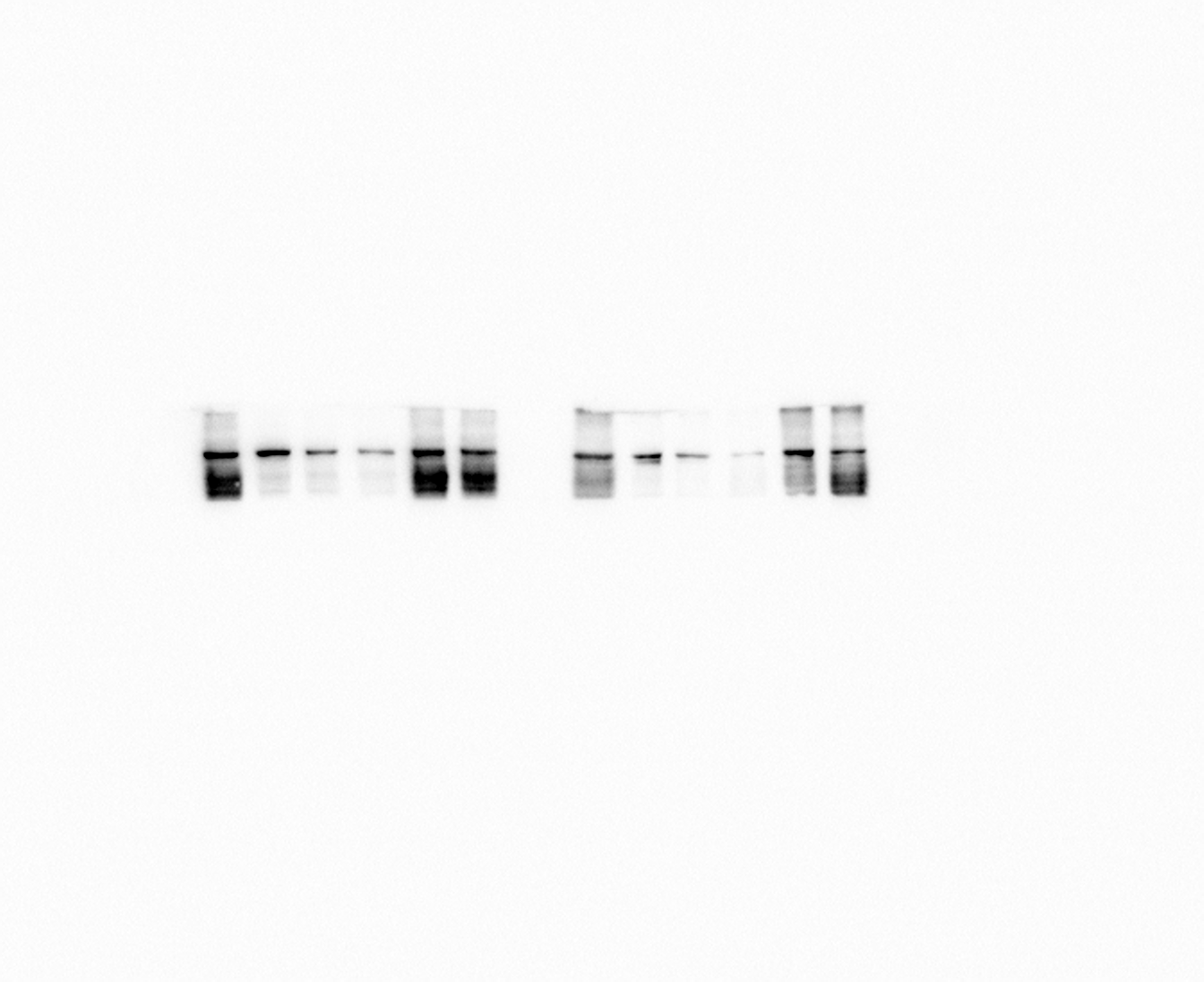

Supplement: Supplementary file 1 [file cancers-16-03028-s001.zip › File S1/Supplement/549/549-ASCT2-1S.Tif]

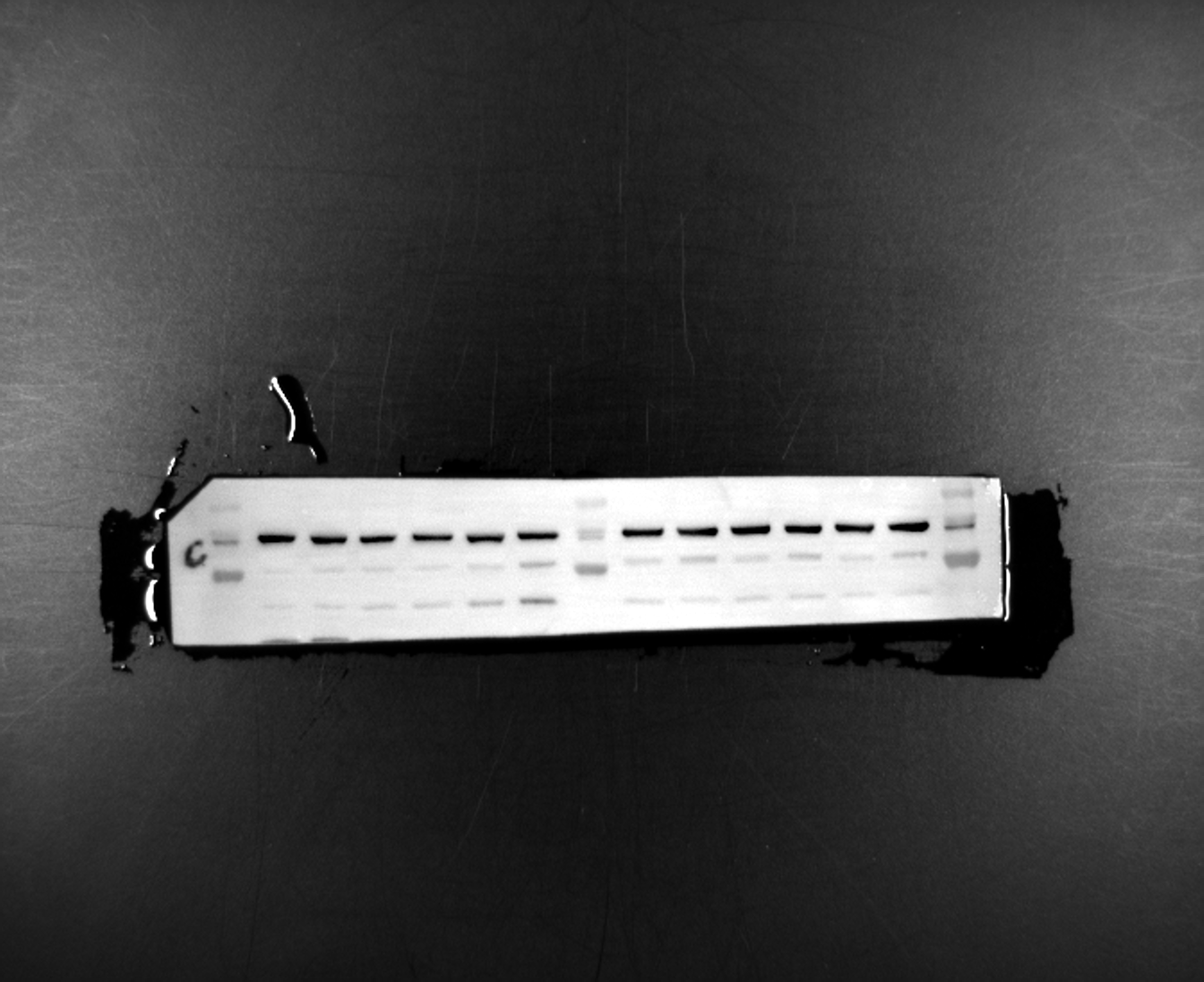

Supplement: Supplementary file 1 [file cancers-16-03028-s001.zip › File S1/Supplement/549/549-CPT1B-0.5S-M.Tif]

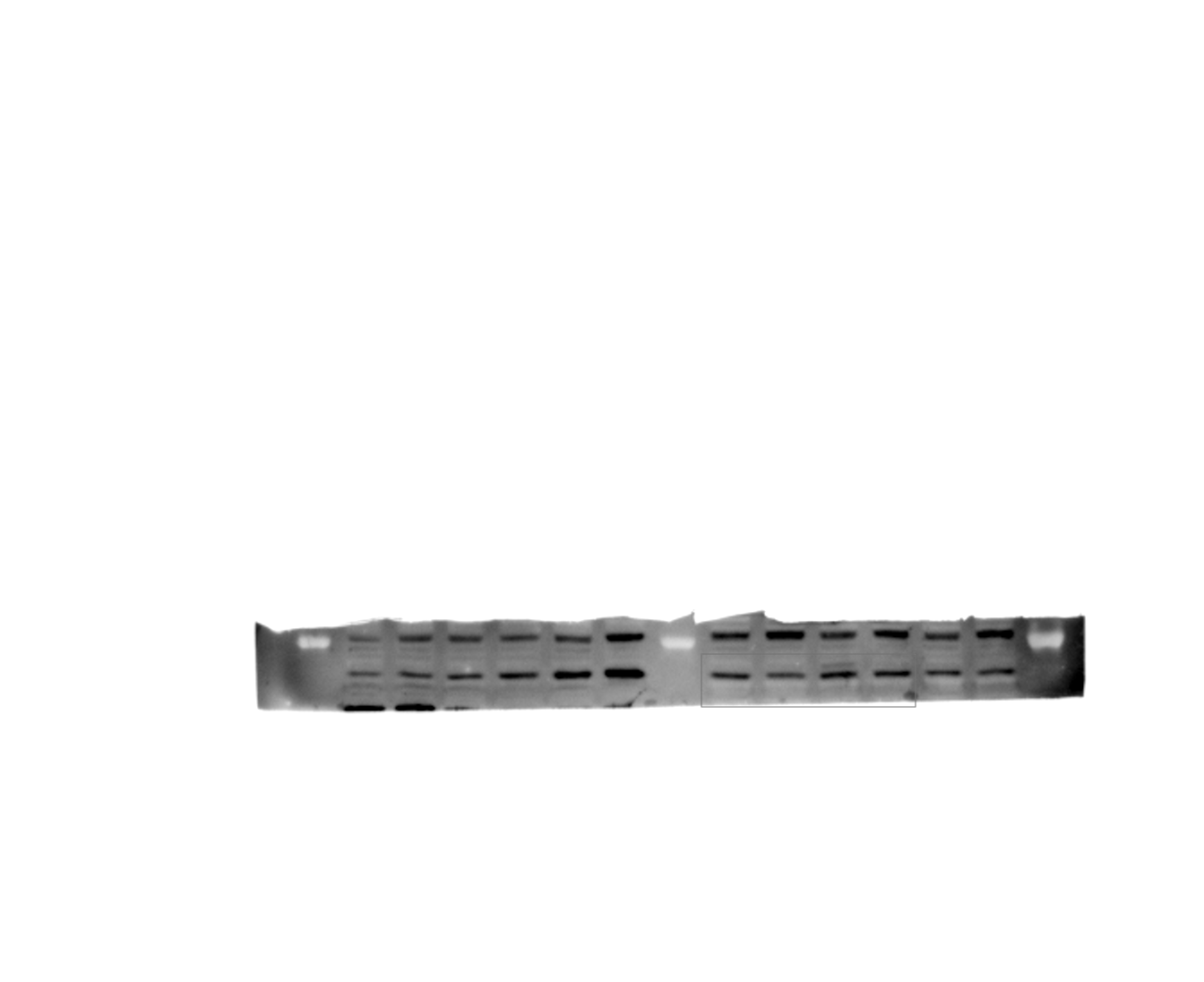

Supplement: Supplementary file 1 [file cancers-16-03028-s001.zip › File S1/Supplement/549/549-CPT1B-10S-.Tif]

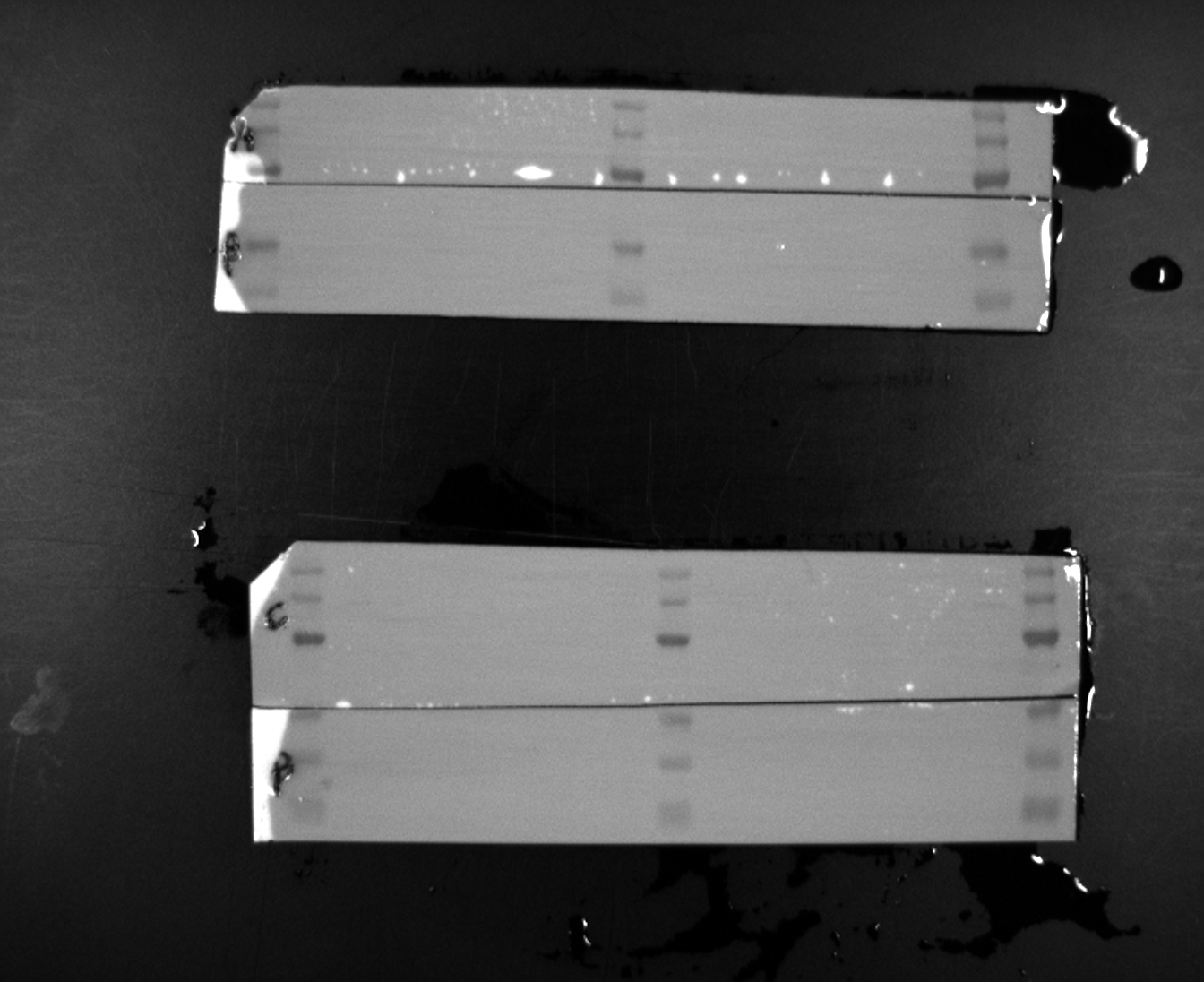

Supplement: Supplementary file 1 [file cancers-16-03028-s001.zip › File S1/Supplement/549/549-Z.Tif]

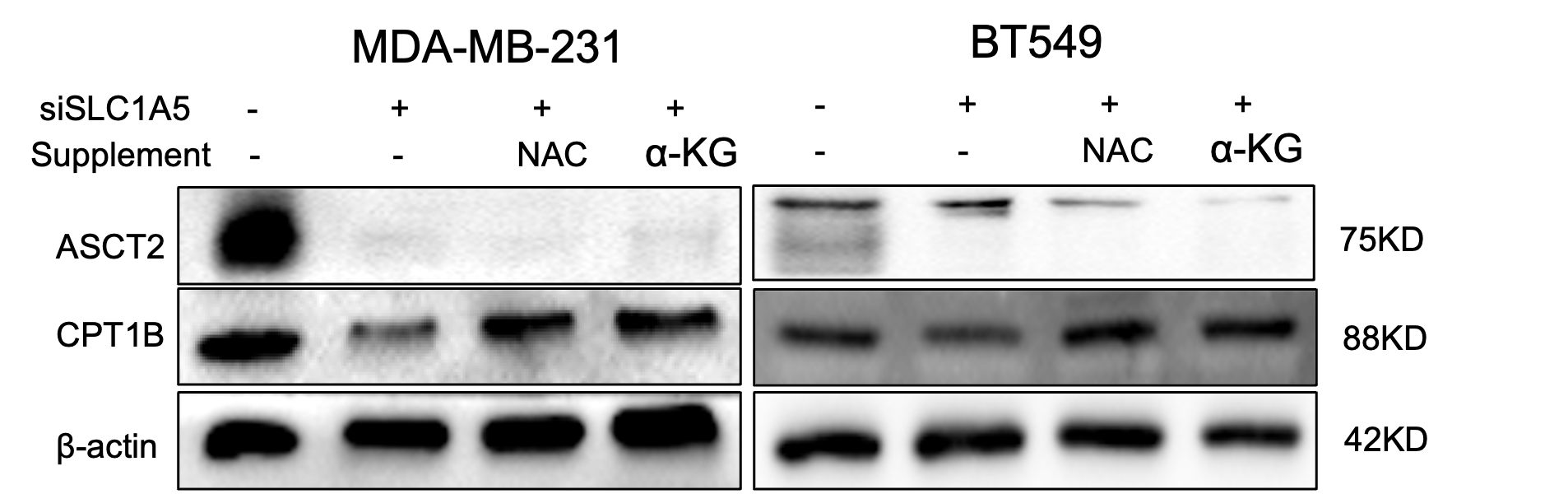

Supplement: Supplementary file 1 [file cancers-16-03028-s001.zip › File S1/Supplement/sum.png]
